# Bioinformatic Interrogation of 5p-arm and 3p-arm Specific miRNA Expression Using TCGA Datasets

**DOI:** 10.3390/jcm4091798

**Published:** 2015-09-15

**Authors:** Wei-Ting Kuo, Ming-Wei Su, Yungling Leo Lee, Chien-Hsiun Chen, Chew-Wun Wu, Wen-Liang Fang, Kuo-Hung Huang, Wen-chang Lin

**Affiliations:** 1 Institute of Biomedical Sciences, Academia Sinica, Taipei 115, Taiwan; E-Mails: raxkuo@hotmail.com (W.-T.K.); a8802137@gmail.com (M.-W.S.); leolee@ntu.edu.tw (Y.L.L.); chchen@ibms.sinica.edu.tw (C.-H.C.); 2 Institute of Biotechnology in Medicine, National Yang-Ming University, Taipei 112, Taiwan; 3 Institute of Epidemiology and Preventive Medicine, National Taiwan University, Taipei 100, Taiwan; 4 Department of Surgery, Veterans General Hospital and National Yang-Ming University, Taipei 112, Taiwan; E-Mails: chewwunwu@gmail.com (C.-W.W.); wlfang@vghtpe.gov.tw (W.-L.F.); khhuang@vghtpe.gov.tw (K.-H.H.)

**Keywords:** microRNA, 5p-arm, 3p-arm, bioinformatics, TCGA, biomarker

## Abstract

MicroRNAs (miRNAs) play important roles in cellular functions and developmental processes. They are also implicated in oncogenesis mechanisms and could serve as potential cancer biomarkers. Using high-throughput miRNA sequencing information, expression of both the 5p-arm and 3p-arm mature miRNAs were demonstrated and generated from the single miRNA hairpin precursor. However, current miRNA annotations lack comprehensive 5p-arm/3p-arm feature annotations. Among known human mature miRNAs, only half of them are annotated with arm features. This generated ambiguous results in many miRNA-Sequencing (miRNA-Seq) studies. In this report, we have interrogated the TCGA (the Cancer Genome Atlas) miRNA expression datasets with an improved, fully annotated human 5p-arm and 3p-arm miRNA reference list. By utilizing this comprehensive miRNA arm-feature annotations, enhanced determinations and clear annotations were achieved for the miRNA isoforms (isomiRs) recognized from the sequencing reads. In the gastric cancer (STAD) dataset, as an example, 32 5p-arm/3p-arm specific miRNAs were found to be down-regulated and 24 5p-arm/3p-arm specific miRNAs were found to be up-regulated. We have further extended miRNA biomarker discoveries to additional TCGA miRNA-Seq datasets and provided extensive expression information on 5p-arm/3p-arm miRNAs across multiple cancer types. Our results identified several miRNAs that could be potential common biomarkers for human cancers.

## 1. Introduction

Cancer is one of the most devastating human diseases [1] and there are devoted efforts to improve cancer treatments. With only limited successes in new anti-cancer drug discovery for clinical usages, it is generally recognized that early diagnosis and surgical resection are the most effective therapeutic procedures for curing human cancers. However, early discovery of cancer is not feasible for most cancer types due to the lack of useful and convenient non-invasive screening biomarkers. The current clinical serum based protein biomarkers for cancers are often unsatisfactory and lack specificity [2]. Therefore, there are substantial research efforts in many countries to identify better biomarkers for early cancer diagnosis and detection. 

MicroRNAs (miRNAs) have become the emerging potential cancer biomarkers in recent years [3,4,5,6]. They are small RNA molecules, which are derived from endogenous non-protein-coding gene transcripts [7,8]. Extensive studies have implicated that miRNAs could play significant roles in tumorigenesis mechanisms and cancer malignant progression [9,10,11,12]. Intriguingly, miRNAs can be released from cancer cells into body fluids via secreting exosomes particles [6,13]. Therefore, circulating miRNAs could be utilized as novel liquid biopsy biomarkers [14,15,16,17,18,19,20]. In the miRNA biogenesis processes, the primary miRNA transcripts are transcribed and cleaved by the Drosha enzyme before being exported to the cytoplasm. They are further processed by the Dicer enzyme to generate the mature miRNA duplex [21,22,23]. Subsequently, one arm of the mature miRNA duplex is preferentially selected to form the ultimate RNA-induced silencing complex and the other arm of the duplex (miR-star) is often degraded [24]. However, with the increasing depth of Next Generation Sequencing (NGS) data, scientists have observed that both arms (strands) of the miRNA duplex could be utilized by the RNA-induced silencing complex (RISC) [25,26]. Therefore, 5p-arm and 3p-arm feature assignments would be essential to clearly distinguish the expressed miRNAs from the same pre-miRNAs during analysis. Thus, missing or incomplete arm feature annotations on human miRNAs might generate ambiguous miRNA data interpretations.

In previous report, we have established a comprehensive arm feature annotation list on almost all known human miRNAs in order to better understand the intrinsic properties of 5p-arm and 3p-arm miRNAs [27]. In this report, we have utilized such an annotated 5p-arm and 3p-arm miRNA list to further analyze the TCGA (the Cancer Genome Atlas) miRNA-Seq dataset for the interrogation of miRNAs as useful cancer biomarkers. The Cancer Genome Atlas is a comprehensive and coordinated effort to accelerate the understanding of the molecular basis of cancer through the application of various genome analysis technologies, including miRNA-Seq [28]. With the large collection of miRNA NGS data, our results demonstrated that the arm-specific miRNA expression profile would be beneficial for thorough analysis of dys-regulated miRNAs in human cancers.

## 2. Experimental Section

### 2.1. Arm Feature Assignment of Human Mature miRNAs

We downloaded all known miRNA information from miRBase release 20 (miRNA.dat, hairpin.fa and mature.fa), which contained 24,521 miRNA precursors and 30,424 mature miRNA sequences [29,30]. There were 206 species reported and we classified them using the species prefix for subsequent analysis. Among all reported miRNAs, there were only 15,398 miRNAs annotated with arm features. We assigned the arm features according to each individual species. For the human genome, there are 2578 mature miRNAs reported. To generate 5p-arm feature/3p-arm feature annotations, we adapted a mapping and classification strategy similar to those used by Zhou *et al.* [26]. In brief, the arm features of all mature miRNAs were annotated by mapping them back to the hairpin precursor sequences using the bowtie program as described [27]. The hairpin precursors were first divided into 5p-arm strand regions (37.5% of the hairpin length), loop regions (25% of the hairpin length) and 3p-arm strand regions (37.5% of the hairpin length) from their 5′-end starting positions. Assignment of the arm features was performed according to the bowtie mapping results (5p-arm or 3p-arm), and we discarded the miRNA records mapped to the loop regions. For human miRNAs, we have assigned 1297 miRNAs with 5p-arm and 1279 miRNAs with 3p-arm. Only two miRNAs were mapped to the loop region; therefore, they could not be assigned to the 5p-arm or 3p-arm. The complete human miRNA 5p-arm and 3p-arm annotation list is provided as the supplementary table. Python scripts were developed to process all data and analysis results using the Linux server (running Scientific Linux 6). 

### 2.2. miRNA-Seq Datasets from TCGA

We obtained the level three miRNA-Seq data from TCGA website excluding cancer types with low numbers of tissue samples [28]. The final 13 TCGA cancer type datasets retrieved included: bladder urothelial carcinoma (BLCA), breast invasive carcinoma (BRCA), head and neck squamous cell carcinoma (HNSC), kidney chromophobe carcinoma (KICH), kidney renal clear cell carcinoma (KIRC), kidney renal papillary cell carcinoma (KIRP), liver hepatocellular carcinoma (LIHC), lung adenocarcinoma (LUAD), lung squamous cell carcinoma (LUSC), prostate adenocarcinoma (PRAD), Thyroid carcinoma (THCA), Uterine corpus endometrial carcinoma (UCEC), and stomach adenocarcinoma (STAD). In summary, we obtained 3972 tumor samples and 578 normal (adjacent tumor) samples. All datasets were processed and calculated for rpm (reads per million). We also excluded libraries with less than one million reads in subsequent miRNA expression analysis. 

### 2.3. Bioinformatic Analysis with Comprehensive Arm-Feature-Annotated miRNA

In order to measure the 5p-arm and 3p-arm miRNA expression, we used the top three expression miRNA isoforms (isomiRs) from 5p-arm and 3p-arm miRNA regions to represent the expression level of 5p-arm miRNAs and 3p-arm miRNAs. For each 5p-arm miRNA or 3p-arm miRNA, we filtered out the lowly expressed miRNAs (rpm less than one). We selected only miRNAs expressed in over 50% of the TCGA libraries for comparison analysis. In the case of specifically interrogating miRNA precursor loci expression, we then combined the 5p-arm and 3p-arm expression reads and calculated their rpm values. Analysis of variance (ANOVA) was performed to identify differentially expressed miRNAs or arm-specific miRNAs using Partek Genomic Suite software (St. Louis, MO, USA).

## 3. Results and Discussion

Due to the significance of miRNAs in cancer development and progression, there are many studies interrogating the roles of miRNAs in human cancers [4,5,12,31], including the TCGA project [28]. However, detailed analysis on the specific expression of miRNA arms across different cancer types is lacking. In order to comprehensively interrogate the expression of miRNA arms, we have obtained the TCGA miRNA-Seq data in 13 different cancer types with the total of 3972 cancer tissue samples and 578 normal (adjacent tumor) tissues. As in the available TCGA miRNA-Seq dataset, we observed that opposite arm miRNAs were often neglected since TCGA pipeline used the standard miRBase annotations. Therefore, our analysis pipeline here would be helpful to assign the expression values of all possible 5p-arm and 3p-arm miRNAs in the TCGA datasets.

### 3.1. Arm Features and isomiR Quantifications

**Figure 1:**
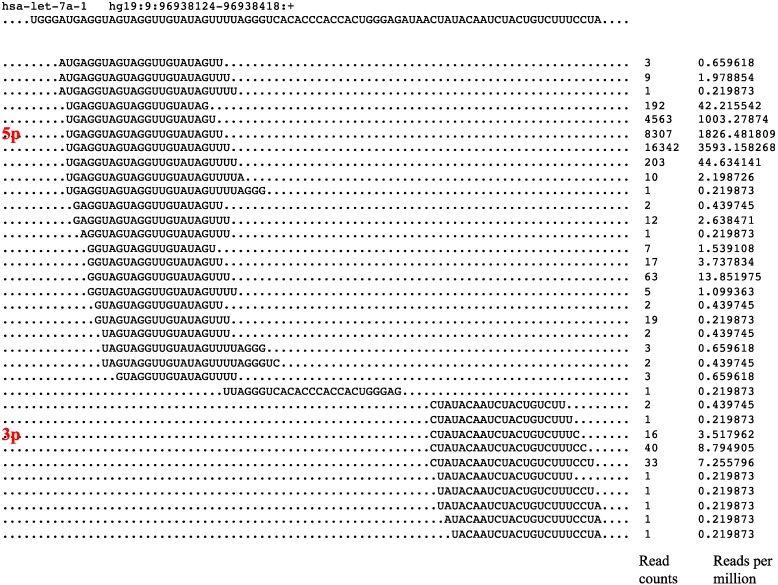
Next Generation Sequencing (NGS) reads alignments of hsa-let-7a-1 miRNA isoforms (isomiRs). A small RNA NGS library from a gastric cancer cell line (AGS cells) was prepared and sequenced with Illumina Solexa platform. The NGS reads were aligned to the hsa-let-7a-1 miRNA genomic locus using Bowtie mapping program following adaptor trimming. We allowed no mismatch at the mapping procedure using standard Bowtie parameter. We trimmed the last 3′ end mismatch one by one until the mapping perfect-match reads were at least 18 nucleotides in length [25]. Here, the hsa-let-7a-1 miRNA precursor sequences and genomic coordinates are displayed on the top section. The NGS reads are aligned and their sequences, read counts and rpm (reads per million) values are displayed. The miRBase annotated hsa-let-7a-1-5p (MIMAT0000062) and hsa-let-7a-1-3p (MIMAT0004481) are marked with 5p and 3p in red, respectively.

In a previous report [27], we analyzed the reported 30,424 mature miRNAs in miRBase, and there were only 15,398 miRNAs annotated with arm features. To resolve this incomplete annotation limitation, we have mapped all un-assigned mature miRNA sequences to their respective precursor miRNA sequences. For human miRNAs, there are 2578 reported mature miRNAs, and we have assigned 1297 miRNAs with 5p-arm and 1279 miRNAs with 3p-arm. Only two miRNAs were mapped to the loop region; therefore, they could not be assigned to the 5p-arm or 3p-arm. Following the arm feature annotation, additional issues to be clarified are the determination and quantification of isomiRs [32]. Typically, reported mature miRNA expressions are only annotated by matching with the known mature miRNA sequences reported (as a defined length and nucleotide sequences). However, it is often observed that length and sequence variants of the reported miRNAs could be readily seen from the NGS data (Figure 1, hsa-let-7a-1 as an example). These miRNA isoforms or variants were named isomiRs. 

As reported previously, there are many mature miRNA isoforms of the same pre-miRNA gene loci that existed following the NGS reads mapping procedures [25]. This isomiR phenomenon existed in all TCGA miRNA-Seq datasets. It has been reported that isomiRs could also associate with RISC and be involved in the target mRNA silencing [26]. This would generate issues in quantification of miRNA expression, since we should not ignore the existence of isomiRs. One can certainly use the miRBase annotated mature miRNA as the only standard for quantification, but this would miss some of the un-annotated opposite arm miRNAs. Besides, in some miRNA loci, we observed that the most abundant isomiR is not necessary the one annotated by miRBase [25]. In Figure 1, as an example, the miRBase reported that hsa-let-7a-1-5p (ugagguaguagguuguauaguu; MIMAT0000062; labeled with 5p) and hsa-let-7a-1-3p (cuauacaaucuacugucuuuc; MIMAT0004481; labeled with 3p) are not the most abundantly expressed isomiRs in this gastric cancer NGS dataset. Therefore, we believe that it is not practical to use just the miRBase annotated mature miRNA sequence as the sole standard expression reference for NGS data quantification. Another way of tabulating is to include all isomiRs mapped to the pre-miRNA locus to cover all the length and sequence isomiR variants in some reported analysis pipelines. However, this would include both the 5p-arm and 3p-arm mature miRNAs expressions into the same miRNA loci. This might be acceptable and utilized in earlier miRNA expression studies with only single mature miRNA arm expected and annotated. Nevertheless, this is not satisfactory with current understanding of NGS datasets with both arms of miRNAs recognized. Therefore, with the comprehensive 5p-arm and 3p-arm miRNA list, we suggested that it is better to carefully quantify the expression of 5p-arm and 3p-arm miRNAs separately. 

**Figure 2:**
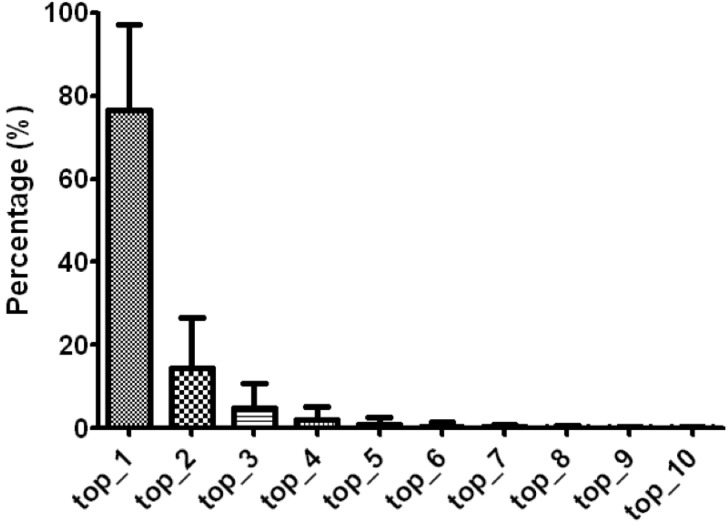
Expression level distribution of miRNA isoform (isomiR) reads. The read counts of each isomiR were tabulated and ranked by their expression percentage within each miRNA gene loci, *i.e.*, read counts of each isomiR divided by the total read counts of all isomiRs in the miRNA gene loci. The top ten expressed isomiRs percentages are displayed.

After tabulating and ranking the expression value of each isomiRs in different miRNA arms (read counts as well as rpm value), we noted a significant pattern on the uppermost expressed isomiRs. We tabulated the expression of all isomiRs in the 5p-arm and 3p-arm miRNAs separately, and calculated the distribution percentage of each isomiR. In Figure 2, we observed that the most abundantly expressed isomiR represented around 80% of the total expression level of all isomiRs. This observation is similar in the 5p-arm miRNA group as well as in 3p-arm miRNA group, respectively. Again, the topmost expressed isomiR is not necessarilyy the one annotated by the miRBase. In addition, the highest three expressed isomiRs could cover nearly 95% of the total expression amounts. Thus, we propose using read counts or rpm values of the uppermost three isomiR expression as the expression level of the 5p-arm and 3p-arm miRNAs. This procedure would be particularly beneficial in determining the un-annotated opposite arm miRNA expression level for those miRNAs with only one single arm miRNA reported by miRBase, since there are no official defined mature miRNA sequences and lengths. 

### 3.2. TCGA miRNA-Seq Analysis: STAD Gastric Cancer Dataset

We then applied this analysis pipeline for the obtained 3972 TCGA datasets. Using the gastric cancer (STAD) data as an example, there were 261 cancer samples and 38 normal samples obtained from TCGA. We used the level 3 expression data and convert the read counts and rpm values from the TCGA STAD dataset. As described earlier, TCGA initial analysis used only the miRBase annotation information; therefore, the arm feature was not well annotated. In addition, many opposite arm (lagging strand) miRNAs were not annotated at all. Following our analysis pipeline and assignment of all 5p-arm and 3p-arm miRNA expression values using the top three expressed isomiRs, we obtained a comprehensive 5p-arm and 3p-arm miRNA expression profile in STAD gastric cancers. 

**Figure 3:**
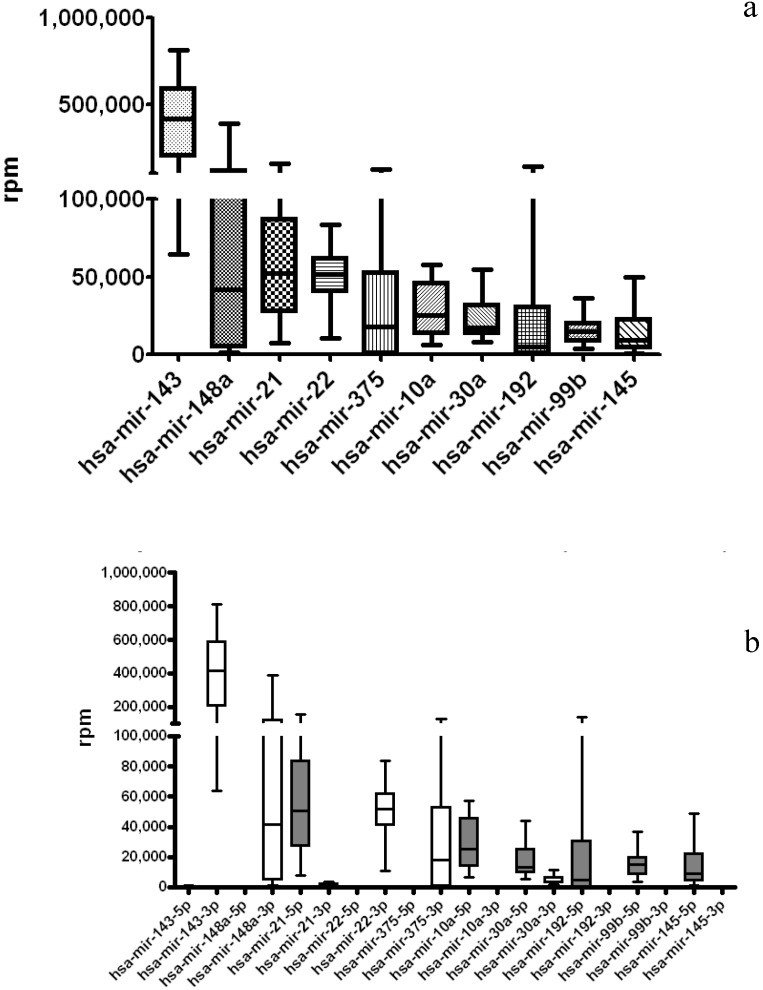
Distribution of the top ten expressed miRNAs from the TCGA (the Cancer Genome Atlas) normal gastric tissue dataset. miRNA expression data of 38 normal samples in TCGA stomach adenocarcinoma (STAD) libraries were re-analyzed using the new arm feature list. (**a**) The rpm (reads per million) values of each miRNAs were tabulated by combining the 5p-arm and 3p-arm together to represent the miRNA loci expression; (**b**) 5p-arm and 3p-arm expression levels were tabulated separately and displayed. miR-143 is the most expressed miRNA in the TCGA clinical normal samples.

We first examined the miRNA gene loci (combined 5p-arm and 3p-arm miRNA together) expression profile in STAD normal samples. In Figure 3A, the utmost ten expressed miRNAs are miR-143, miR-148a, miR-21, miR-22, miR-375, miR-10a, miR-30a, miR-192, miR-99b and miR-145. In order to compare the expression pattern between 5p-arm and 3p-arm miRNAs in detail, we then analyzed the expression values of separated 5p-arm and 3p-arm miRNAs. When one further interrogated the preferential expression on the 5p-arm and 3p-arm miRNAs in details, preferential expression of single miRNA arm is noted in these highly expressed miRNAs (Figure 3b). Six miRNAs have higher expression levels in 5p-arm and 4 miRNAs have more expression in 3p-arm.

**Figure 4:**
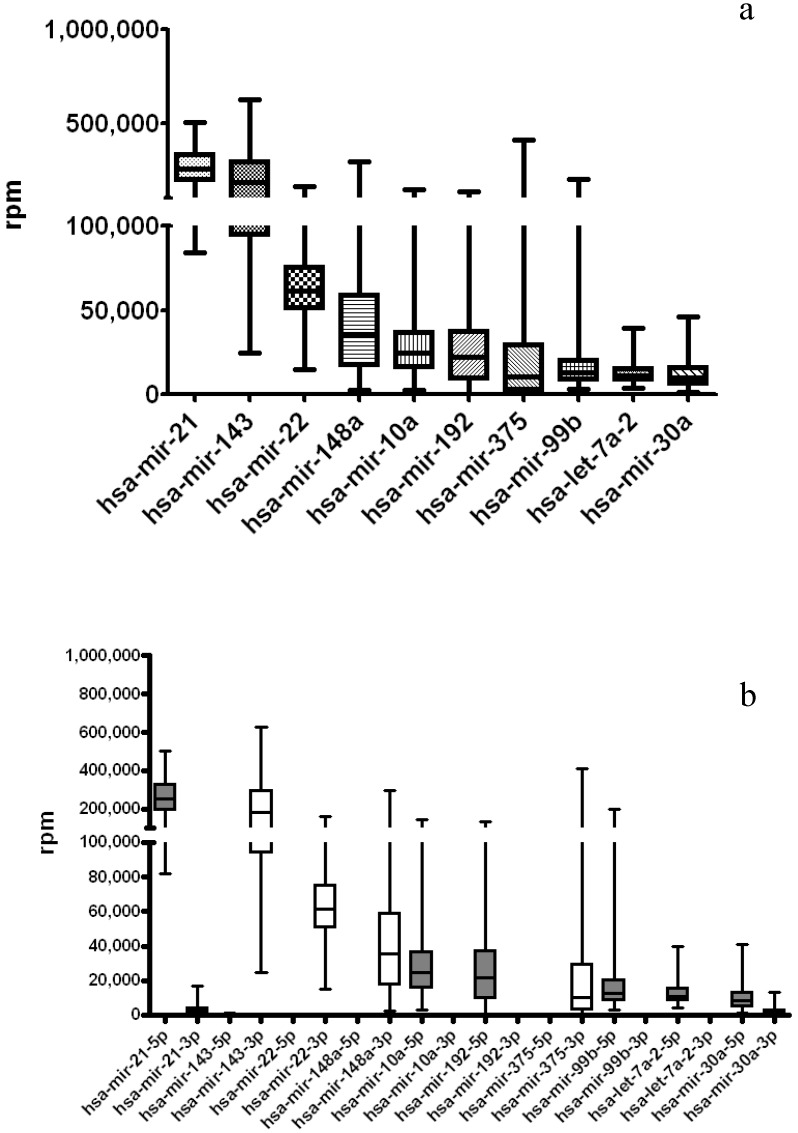
Distribution of highly expressed miRNAs in 261 gastric cancer samples from TCGA (The Cancer Genome Atlas) datasets. miRNA expression data of TCGA stomach adenocarcinoma (STAD) were retrieved and re-analyzed using the comprehensive arm feature annotated miRNA list. (**a**) The rpm values of each miRNAs were tabulated by combining the 5p-arm and 3p-arm to represent the miRNA loci expression; (**b**) 5p-arm and 3p-arm expression levels were tabulated separately and displayed. miR-21 is the most expressed miRNAs in the TCGA clinical cancer samples.

Similar miRNA expression patterns were observed in the STAD cancer samples (Figure 4). The highly expressed miRNA genes (combined 5p-arm and 3p-arm together) in the STAD cancer group are: miR-21, miR-143, miR-22, miR-148a, miR-10a, miR-192, miR-375, miR-99b, let-7a-2 and miR-30a. The 5p-arm miRNA and 3p-arm miRNA expression dominance pattern is also similar in the STAD cancer samples (Figure 4b) following the examination on separated 5p-arm and 3p-arm expression levels. Finally, we observe most of highly expressed miRNAs in both the normal and cancer STAD groups, including miR-143, miR-21, miR-22, miR-148a, miR-10a, miR-192, miR-375, miR-99b and miR-30a. However, it is significant to observe the increase of expression rpm numbers of miR-21, specifically miR-21-5p. This implied the significant role of miR-21-5p in gastric cancer oncogenesis as previously reported [18,33]. 

**Figure 5:**
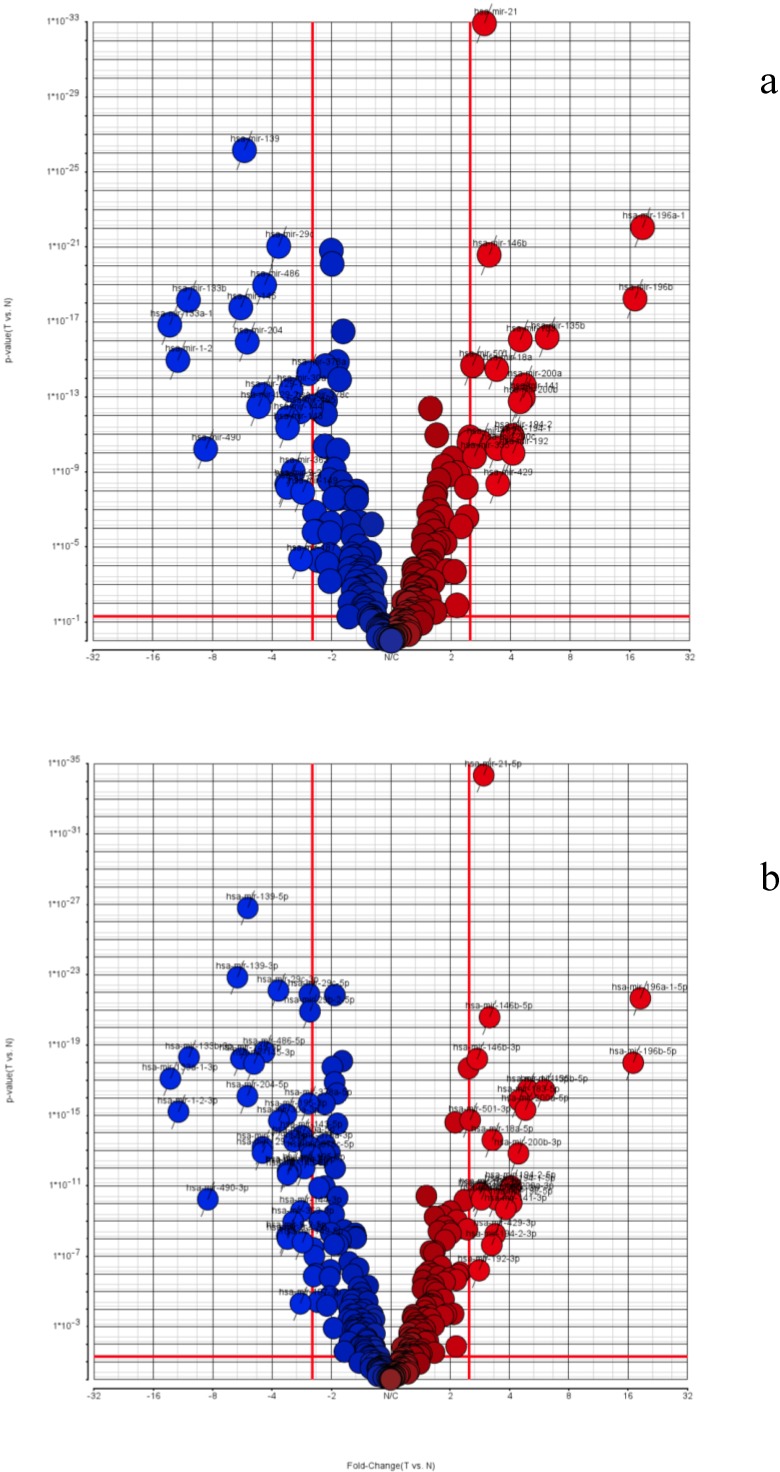
Volcano plot of combined miRNA expression and separate 5p-arm/3p-arm miRNA expression in TCGA (The Cancer Genome Atlas) gastric cancer tissues. miRNA expression data of TCGA stomach adenocarcinoma (STAD) were retrieved and re-analyzed using the comprehensive arm feature annotated miRNA list. The miRNA expression information of gastric cancer tissues is illustrated here by calculating the mean expression level from TCGA samples. Following analysis of variance (ANOVA) in the Partek software package, the volcano plot is displayed for selecting differentially expressed miRNA genes. (**a**) Precursor miRNAs gene loci expression by combining the 5p-arm and 3p-arm miRNAs together for analysis; (**b**) 5p-arm and 3p-arm miRNAs tabulated separately for their expression and analyzed.

Finally, we used ANOVA analysis to identify the significantly expressed miRNAs in the TCGA STAD dataset. We filtered out the miRNAs with low expression (rpm less than one) and selected only miRNAs expressed in over 50% of the TCGA STAD sample libraries. The subsequent normalization and ANOVA analysis were performed by the Partek software package. In Figure 5a, with the selection criteria of fold-change value > 2.5 and *p*-value < 0.05, we interrogated the miRNA gene loci expression by the combined 5p-arm and 3p-arm expression values. We identified 22 down-regulated miRNAs and 18 up-regulated miRNAs from close to 300 clinical STAD samples (Table 1; overall miRNA precursor). Among these 40 miRNAs, 23 miRNAs have been reported to have significant association with human gastric cancers in a previous review paper [3]. With our new analysis pipeline, we could achieve better resolutions and coverage on the arm-specific isomiR expressions. We further interrogated the separate expression levels of 5p-arm miRNAs and 3p-arm miRNAs. In the 5p-arm and 3p-arm separated analysis group, there are 32 miRNAs down-regulated and 24 miRNAs up-regulated (Figure 5b and Table 1; separate 5p-arm and 3p-arm miRNAs). More arm-specific miRNAs were identified here in the arm separate group as expected (56 arm-specific miRNAs *vs*. 40 miRNA genes). In the up-regulated miRNAs, we found the five miRNAs have both 5p-arm and 3p-arm expression significantly increased (miR-146b, miR-200a, miR-141 miR-192 and miR-194). In the down-regulated group, there are seven miRNA pairs (miR-139, miR-29c, miR-145, miR-378, miR-30a, miR-143 and miR-144) with both the 5p-arm and 3p-arm identified as significantly dys-regulated miRNAs. Many of the miRNAs were also reported by a recent systems biology paper from TCGA in 2014 [34]. 

We further compare the expression pattern between 5p-arm and 3p-arm miRNAs in more detail. We selected only miRNAs expressing both 5p-arm and 3p-arm miRNAs for analysis, and there are 196 miRNAs identified from the TCGA STAD library with both the 5p-arm and 3p-arm expressed. It is noted there is a significant expression level difference between the guide strand miRNAs and passenger strand miRNAs (miRNA*) as researchers have previously noted [25,35,36,37]. If one investigates the 5p-arm/3p-arm expression ratios of these single arm dominant miRNAs across over 4000 TCGA samples examined here, there is no significant difference between 5p-arm expression dominance and 3p-arm expression dominance miRNA populations. Similar findings on consistency of isomiR expression profiles in different cell types were also reported by Guo *et al.* [38]. Therefore, the arm-switching events were not detected among the large numbers of TCGA data examined here. This also illustrated the advantages of our arm-feature annotation efforts to provide clearer and better miRNA analysis results systematically in large numbers of samples. The arm-switching phenomenon is mentioned previously by observing that the arm from which the dominant mature miRNA is processed can switch in different tissues or developmental periods [39]. It is believed that arm selection is governed by the asymmetrical stability of hairpins and that the determinant sequences critical to arm dominance is outside the mature miRNA duplex. Just recently, in addition to the secondary hairpin structure, it has been reported that certain primary sequence motifs are also required in hairpin recognition and processing, including the downstream SRp20-binding motif, the basal UG motif in the stem, and the apical stem GUG motif [40]. 

**Table 1:** Dys-regulated miRNAs in TCGA (the Cancer Genome Atlas) STAD gastric cancer tissues.

TCGA STAD (Stomach Adenocarcinoma)			
Overall miRNA Precursors		Separate 5p-arm and 3p-arm miRNAs	
Down regulated	Up regulated	Down regulated	Up regulated
hsa-miR-139	hsa-miR-21	hsa-miR-139-5p	hsa-miR-21-5p
hsa-miR-29c	hsa-miR-196a-1	hsa-miR-139-3p	hsa-miR-196a-1-5p
hsa-miR-486	hsa-miR-146b	hsa-miR-29c-3p	hsa-miR-146b-5p
hsa-miR-133b	hsa-miR-196b	hsa-miR-29c-5p	hsa-miR-146b-3p
hsa-miR-145	hsa-miR-135b	hsa-miR-29b-2-5p	hsa-miR-196b-5p
hsa-miR-133a-1	hsa-miR-183	hsa-miR-486-5p	hsa-miR-141-5p
hsa-miR-204	hsa-miR-501	hsa-miR-133b-3p	hsa-miR-135b-5p
hsa-miR-1-2	hsa-miR-18a	hsa-miR-145-5p	hsa-miR-183-5p
hsa-miR-378a	hsa-miR-200a	hsa-miR-145-3p	hsa-miR-200a-5p
hsa-miR-30a	hsa-miR-141	hsa-miR-133a-1-3p	hsa-miR-501-3p
hsa-miR-129-1	hsa-miR-200b	hsa-miR-204-5p	hsa-miR-18a-5p
hsa-miR-129-2	hsa-miR-194-2	hsa-miR-378a-5p	hsa-miR-200b-3p
hsa-miR-378c	hsa-miR-194-1	hsa-miR-1-2-3p	hsa-miR-194-2-5p
hsa-miR-195	hsa-miR-182	hsa-miR-195-3p	hsa-miR-194-1-5p
hsa-miR-144	hsa-miR-200c	hsa-miR-30a-3p	hsa-miR-335-3p
hsa-miR-143	hsa-miR-192	hsa-miR-143-5p	hsa-miR-182-5p
hsa-miR-490	hsa-miR-335	hsa-miR-30a-5p	hsa-miR-200a-3p
hsa-miR-363	hsa-miR-429	hsa-miR-378a-3p	hsa-miR-200c-3p
hsa-miR-9-2		hsa-miR-129-1-5p	hsa-miR-708-5p
hsa-miR-9-1		hsa-miR-129-2-5p	hsa-miR-192-5p
hsa-miR-149		hsa-miR-378c-5p	hsa-miR-141-3p
hsa-miR-187		hsa-miR-195-5p	hsa-miR-429-3p
		hsa-miR-30c-2-3p	hsa-miR-194-2-3p
		hsa-miR-144-5p	hsa-miR-192-3p
		hsa-miR-143-3p	
		hsa-miR-490-3p	
		hsa-miR-144-3p	
		hsa-miR-363-3p	
		hsa-miR-9-2-5p	
		hsa-miR-9-1-5p	
		hsa-miR-149-5p	
		hsa-miR-187-3p	

### 3.3. miRNA-Seq Analysis on Additional 12 TCGA Cancer Types

**Figure 6:**
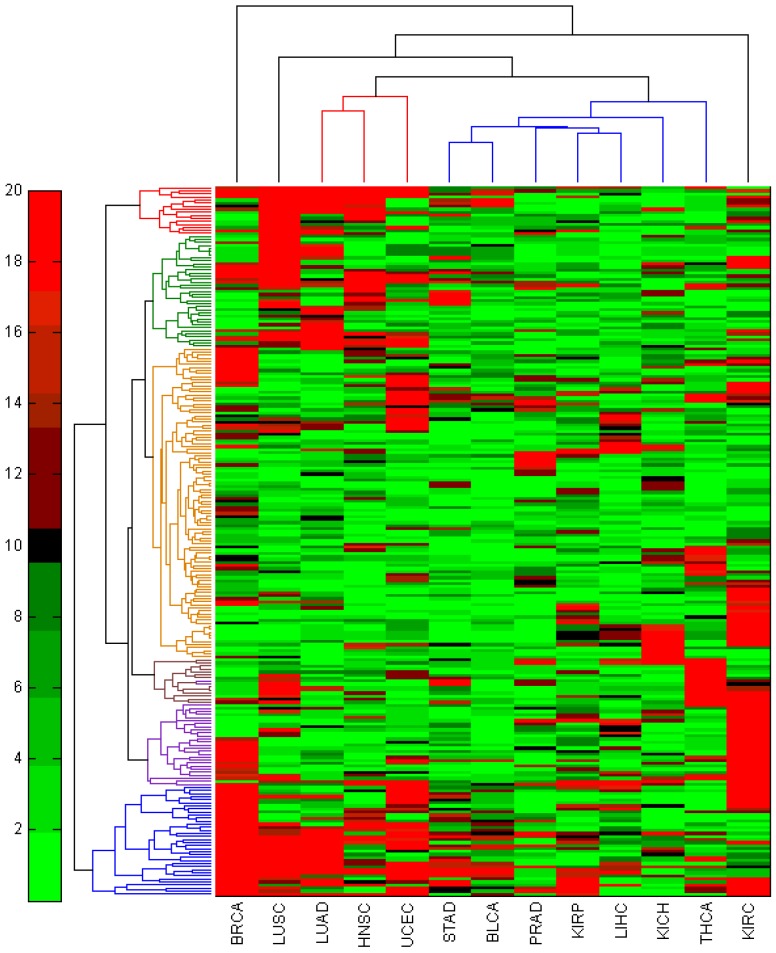
Hierarchical clustering analysis on significantly dys-regulated miRNAs in 13 TCGA (the Cancer Genome Atlas) cancer types. 233 arm-specific miRNAs were expressed in all 13 cancer types and further selected for hierarchical clustering analysis to reveal their significance in each cancer type.

By using a comprehensive 5p-arm and 3p-arm miRNA reference list, our analysis pipeline could provide clear and comprehensive miRNA expression profiles. Since there are no systematic examinations on arm specific miRNAs, we are interested in interrogating the arm-specific miRNAs dys-regulated in different cancers. We further applied this analysis pipeline to interrogate other miRNA-Seq datasets from TCGA, especially on the 5p-arm and 3p-arm annotated miRNAs. The same criteria (fold-change value > 2.5 and *p*-value < 0.05) were applied to identify significantly dys-regulated arm-specific miRNAs (Supplementary Figures S1a to S1l). It is interesting to note that the numbers of dys-regulated miRNAs varied between different cancer types, which might be related to the diverse sample size and library qualities, since we do filter out low-expression miRNAs. Many of the miRNAs identified in each cancer type were reported in the literatures [3]. Here, we would like to inquire if any of the dys-regulated could be utilized as potential cancer biomarkers for most cancer types, which would be beneficial to serve as routine cancer screening biomarkers. We first explored arm-specific miRNAs found in all cancer types analyzed (233 miRNAs) and then examined the significance distribution of each 5p-arm and 3p-arm miRNA using hierarchical clustering (Figure 6). 

Among these 233 miRNAs, there are several miRNAs seems to be important in the basic oncogenesis processes and found to be significant in multiple cancer types. They could be utilized as general cancer biomarkers [5,33,41,42,43,44,45]. Some of the up-regulated arm specific miRNAs include miR-182-5p, miR-183-5p, miR-21-5p, miR-141-5p, miR-1307-5p, miR-130b-3p, miR-196b-5p, miR-210-3p, miR-21-3p and miR-141-3p. The down-regulated miRNAs include miR-139-5p, miR-139-3p, miR-145-5p, miR-145-3p, miR-486-5p, and miR-1-2-3p. In some cases, we observed the few miRNAs have contradictory associations in different cancer types, which indicate multiple biological functions for some of the miRNAs, such as miR-141-5p and miR-141-3p, miR-486-5p. There are few miRNA 5p-arm and 3p-arm pairs identified in our study: miR-21-5p and miR-21-3p; miR-141-5p and miR-141-3p; miR-139-5p and miR-139-3p; miR-145-5p and miR-145-3p. The 5p-arm mature miRNA is totally different from the 3p-arm mature miRNA in terms of sequences and target spectrum, not to mention expression level. miR-139-5p has been known to be a tumor suppressor miRNA by inhibiting the metastasis pathway [46]; however, miR-139-3p has not been well studied and is often neglected, since much literature has only used miR-139. There are few studies suggested that both arms of mature miRNAs (miR-582-5p and miR-582-3p) from a single pri-miRNA locus were cooperatively involved in the modulation of critical cellular pathways in human cancer cells [47]. More studies should be conducted to carefully interrogate 5p-arm and 3p-arm miRNA functions. Therefore, our study provides better annotations and improved understanding of arm-specific miRNAs in human cancers.

## 4. Conclusions

In conclusion, with comprehensive 5p-arm and 3p-arm feature annotations, we could achieve more comprehensive and in-depth investigation on dys-regulated miRNAs in human cancers. In earlier reports, while certain miRNAs were reported with the correct arm assignment annotations, the arm annotation information was often lacking in other reports. This would generate confusion in the interpretation of data, since one would not know which arm was being referred to. Using miR-30a as an example, in some papers miR-30a-3p is a signature biomarker for breast cancer recurrence [4], and another study mentioned miR-30a-5p as a tumor-suppressive miRNA in colon cancer [48]. Nonetheless, we could encounter literature lacking clear descriptions of the miR-30a 5p-arm or miR-30a 3p-arm, which would create uncertainty and bafflement for people interested in their studies. Thus, it is beneficial to have complete, comprehensive 5p-arm/3p-arm assignment for all human miRNAs. This is especially important for NGS miRNA analysis, since more miRNA reads from both 5p-arm and 3p-arm could be observed with the increasing depth of sequencing. By utilizing the comprehensive miRNA arm-feature annotations, we could improve the miRNA expression pipeline with better and well-defined annotated miRNA expression information and provided extended expression information on opposite arms of the miRNA hairpin precursors. With the systematical interrogation of multiple cancer miRNA-Seq datasets from TCGA, we were able to apply our analysis pipeline to discover arm-specific miRNAs important in the oncogenesis processes and useful as common cancer biomarkers for different cancer types.

## Supplementary Files

Supplementary File 1
