# Supplementary material for: Bioinformatic Interrogation of 5p-arm and 3p-arm Specific miRNA Expression Using TCGA Datasets
Source: J Clin Med. 2015 Sep 15;4(9):1798–814. doi: 10.3390/jcm4091798 (PMC4600160; doi:10.3390/jcm4091798)
Supplement: Supplementary File 1 [file jcm-04-01798-s001.pdf]

Supplementary Materials

Table S1. Comprehensive annotation of arm features in human miRNAs.

Order	Precursor	Precursor_accession_number	miRNA_5p	miRNA_5p_accession_number	miRNA_3p	miRNA_3p_accession_number
1_	hsa-let-7a-1	MI0000060	hsa-let-7a-5p	MIMAT0000062	hsa-let-7a-3p	MIMAT0004481
2_	hsa-let-7a-2	MI0000061	hsa-let-7a-5p	MIMAT0000062	hsa-let-7a-2-3p	MIMAT0010195
3_	hsa-let-7a-3	MI0000062	hsa-let-7a-5p	MIMAT0000062	hsa-let-7a-3p	MIMAT0004481
4_	hsa-let-7b	MI0000063	hsa-let-7b-5p	MIMAT0000063	hsa-let-7b-3p	MIMAT0004482
5_	hsa-let-7c	MI0000064	hsa-let-7c-5p	MIMAT0000064	hsa-let-7c-3p	MIMAT0026472
6_	hsa-let-7d	MI0000065	hsa-let-7d-5p	MIMAT0000065	hsa-let-7d-3p	MIMAT0004484
7_	hsa-let-7e	MI0000066	hsa-let-7e-5p	MIMAT0000066	hsa-let-7e-3p	MIMAT0004485
8_	hsa-let-7f-1	MI0000067	hsa-let-7f-5p	MIMAT0000067	hsa-let-7f-1-3p	MIMAT0004486
9_	hsa-let-7f-2	MI0000068	hsa-let-7f-5p	MIMAT0000067	hsa-let-7f-2-3p	MIMAT0004487
10_	hsa-let-7g	MI0000433	hsa-let-7g-5p	MIMAT0000414	hsa-let-7g-3p	MIMAT0004584
11_	hsa-let-7i	MI0000434	hsa-let-7i-5p	MIMAT0000415	hsa-let-7i-3p	MIMAT0004585
12_	hsa-mir-1-1	MI0000651	na	na	hsa-miR-1-3p	MIMAT0000416
13_	hsa-mir-1-2	MI0000437	na	na	hsa-miR-1-3p	MIMAT0000416
14_	hsa-mir-100	MI0000102	hsa-miR-100-5p	MIMAT0000098	hsa-miR-100-3p	MIMAT0004512
15_	hsa-mir-101-1	MI0000103	hsa-miR-101-5p	MIMAT0004513	hsa-miR-101-3p	MIMAT0000099
16_	hsa-mir-101-2	MI0000739	na	na	hsa-miR-101-3p	MIMAT0000099
17_	hsa-mir-103a-1	MI0000109	na	na	hsa-miR-103a-3p	MIMAT0000101
18_	hsa-mir-103a-2	MI0000108	hsa-miR-103a-2-5p	MIMAT0009196	hsa-miR-103a-3p	MIMAT0000101
19_	hsa-mir-103b-1	MI0007261	hsa-miR-103b-5p	MIMAT0007402	na	na
20_	hsa-mir-103b-2	MI0007262	hsa-miR-103b-5p	MIMAT0007402	na	na
21_	hsa-mir-105-1	MI0000111	hsa-miR-105-5p	MIMAT0000102	hsa-miR-105-3p	MIMAT0004516
22_	hsa-mir-105-2	MI0000112	hsa-miR-105-5p	MIMAT0000102	hsa-miR-105-3p	MIMAT0004516
23_	hsa-mir-106a	MI0000113	hsa-miR-106a-5p	MIMAT0000103	hsa-miR-106a-3p	MIMAT0004517
24_	hsa-mir-106b	MI0000734	hsa-miR-106b-5p	MIMAT0000680	hsa-miR-106b-3p	MIMAT0004672
25_	hsa-mir-107	MI0000114	na	na	hsa-miR-107-3p	MIMAT0000104
26_	hsa-mir-10a	MI0000266	hsa-miR-10a-5p	MIMAT0000253	hsa-miR-10a-3p	MIMAT0004555
27_	hsa-mir-10b	MI0000267	hsa-miR-10b-5p	MIMAT0000254	hsa-miR-10b-3p	MIMAT0004556
28_	hsa-mir-1178	MI0006271	hsa-miR-1178-5p	MIMAT0022940	hsa-miR-1178-3p	MIMAT0005823

29_	hsa-mir-1179	MI0006272	hsa-miR-1179-5p	MIMAT0005824	na	na
30_	hsa-mir-1180	MI0006273	hsa-miR-1180-5p	MIMAT0026735	hsa-miR-1180-3p	MIMAT0005825
31_	hsa-mir-1181	MI0006274	hsa-miR-1181-5p	MIMAT0005826	na	na
32_	hsa-mir-1182	MI0006275	na	na	hsa-miR-1182-3p	MIMAT0005827
33_	hsa-mir-1183	MI0006276	na	na	hsa-miR-1183-3p	MIMAT0005828
34_	hsa-mir-1184-1	MI0006277	na	na	hsa-miR-1184-3p	MIMAT0005829
35_	hsa-mir-1184-2	MI0015971	na	na	hsa-miR-1184-3p	MIMAT0005829
36_	hsa-mir-1184-3	MI0015972	na	na	hsa-miR-1184-3p	MIMAT0005829
37_	hsa-mir-1185-1	MI0003844	hsa-miR-1185-5p	MIMAT0005798	hsa-miR-1185-1-3p	MIMAT0022838
38_	hsa-mir-1185-2	MI0003821	hsa-miR-1185-5p	MIMAT0005798	hsa-miR-1185-2-3p	MIMAT0022713
39_	hsa-mir-1193	MI0014205	hsa-miR-1193-5p	MIMAT0015049	na	na
40_	hsa-mir-1197	MI0006656	na	na	hsa-miR-1197-3p	MIMAT0005955
41_	hsa-mir-1199	MI0020340	hsa-miR-1199-5p	MIMAT0031119	hsa-miR-1199-3p	MIMAT0031120
42_	hsa-mir-1200	MI0006332	hsa-miR-1200-5p	MIMAT0005863	na	na
43_	hsa-mir-1202	MI0006334	hsa-miR-1202-5p	MIMAT0005865	na	na
44_	hsa-mir-1203	MI0006335	hsa-miR-1203-5p	MIMAT0005866	na	na
45_	hsa-mir-1204	MI0006337	hsa-miR-1204-5p	MIMAT0005868	na	na
46_	hsa-mir-1205	MI0006338	hsa-miR-1205-5p	MIMAT0005869	na	na
47_	hsa-mir-1206	MI0006339	hsa-miR-1206-5p	MIMAT0005870	na	na
48_	hsa-mir-1207	MI0006340	hsa-miR-1207-5p	MIMAT0005871	hsa-miR-1207-3p	MIMAT0005872
49_	hsa-mir-1208	MI0006341	hsa-miR-1208-5p	MIMAT0005873	na	na
50_	hsa-mir-122	MI0000442	hsa-miR-122-5p	MIMAT0000421	hsa-miR-122-3p	MIMAT0004590
51_	hsa-mir-1224	MI0003764	hsa-miR-1224-5p	MIMAT0005458	hsa-miR-1224-3p	MIMAT0005459
52_	hsa-mir-1225	MI0006311	hsa-miR-1225-5p	MIMAT0005572	hsa-miR-1225-3p	MIMAT0005573
53_	hsa-mir-1226	MI0006313	hsa-miR-1226-5p	MIMAT0005576	hsa-miR-1226-3p	MIMAT0005577
54_	hsa-mir-1227	MI0006316	hsa-miR-1227-5p	MIMAT0022941	hsa-miR-1227-3p	MIMAT0005580
55_	hsa-mir-1228	MI0006318	hsa-miR-1228-5p	MIMAT0005582	hsa-miR-1228-3p	MIMAT0005583
56_	hsa-mir-1229	MI0006319	hsa-miR-1229-5p	MIMAT0022942	hsa-miR-1229-3p	MIMAT0005584
57_	hsa-mir-1231	MI0006321	hsa-miR-1231-5p	MIMAT0005586	na	na
58_	hsa-mir-1233-1	MI0006323	hsa-miR-1233-5p	MIMAT0022943	hsa-miR-1233-3p	MIMAT0005588

59_	hsa-mir-1233-2	MI0015973	hsa-miR-1233-5p	MIMAT0022943	hsa-miR-1233-3p	MIMAT0005588
60_	hsa-mir-1234	MI0006324	na	na	hsa-miR-1234-3p	MIMAT0005589
61_	hsa-mir-1236	MI0006326	hsa-miR-1236-5p	MIMAT0022945	hsa-miR-1236-3p	MIMAT0005591
62_	hsa-mir-1237	MI0006327	hsa-miR-1237-5p	MIMAT0022946	hsa-miR-1237-3p	MIMAT0005592
63_	hsa-mir-1238	MI0006328	hsa-miR-1238-5p	MIMAT0022947	hsa-miR-1238-3p	MIMAT0005593
64_	hsa-mir-124-1	MI0000443	hsa-miR-124-5p	MIMAT0004591	hsa-miR-124-3p	MIMAT0000422
65_	hsa-mir-124-2	MI0000444	hsa-miR-124-5p	MIMAT0004591	hsa-miR-124-3p	MIMAT0000422
66_	hsa-mir-124-3	MI0000445	hsa-miR-124-5p	MIMAT0004591	hsa-miR-124-3p	MIMAT0000422
67_	hsa-mir-1243	MI0006373	hsa-miR-1243-5p	MIMAT0005894	na	na
68_	hsa-mir-1244-1	MI0006379	na	na	hsa-miR-1244-3p	MIMAT0005896
69_	hsa-mir-1244-2	MI0015974	na	na	hsa-miR-1244-3p	MIMAT0005896
70_	hsa-mir-1244-3	MI0015975	na	na	hsa-miR-1244-3p	MIMAT0005896
71_	hsa-mir-1245a	MI0006380	na	na	hsa-miR-1245a-3p	MIMAT0005897
72_	hsa-mir-1245b	MI0017431	hsa-miR-1245b-5p	MIMAT0019950	hsa-miR-1245b-3p	MIMAT0019951
73_	hsa-mir-1246	MI0006381	hsa-miR-1246-5p	MIMAT0005898	na	na
74_	hsa-mir-1247	MI0006382	hsa-miR-1247-5p	MIMAT0005899	hsa-miR-1247-3p	MIMAT0022721
75_	hsa-mir-1248	MI0006383	hsa-miR-1248-5p	MIMAT0005900	na	na
76_	hsa-mir-1249	MI0006384	na	na	hsa-miR-1249-3p	MIMAT0005901
77_	hsa-mir-1250	MI0006385	hsa-miR-1250-5p	MIMAT0005902	hsa-miR-1250-3p	MIMAT0026740
78_	hsa-mir-1251	MI0006386	hsa-miR-1251-5p	MIMAT0005903	hsa-miR-1251-3p	MIMAT0026741
79_	hsa-mir-1252	MI0006434	hsa-miR-1252-5p	MIMAT0005944	hsa-miR-1252-3p	MIMAT0026744
80_	hsa-mir-1253	MI0006387	hsa-miR-1253-5p	MIMAT0005904	na	na
81_	hsa-mir-1254-1	MI0006388	hsa-miR-1254-5p	MIMAT0005905	na	na
82_	hsa-mir-1254-2	MI0016747	hsa-miR-1254-5p	MIMAT0005905	na	na
83_	hsa-mir-1255a	MI0006389	hsa-miR-1255a-5p	MIMAT0005906	na	na
84_	hsa-mir-1255b-1	MI0006435	hsa-miR-1255b-5p	MIMAT0005945	na	na
85_	hsa-mir-1255b-2	MI0006436	hsa-miR-1255b-5p	MIMAT0005945	hsa-miR-1255b-2-3p	MIMAT0022725
86_	hsa-mir-1256	MI0006390	hsa-miR-1256-5p	MIMAT0005907	na	na
87_	hsa-mir-1257	MI0006391	hsa-miR-1257-5p	MIMAT0005908	na	na

88_	hsa-mir-1258	MI0006392	na	na	hsa-miR-1258-3p	MIMAT0005909
89_	hsa-mir-125a	MI0000469	hsa-miR-125a-5p	MIMAT0000443	hsa-miR-125a-3p	MIMAT0004602
90_	hsa-mir-125b-1	MI0000446	hsa-miR-125b-5p	MIMAT0000423	hsa-miR-125b-1-3p	MIMAT0004592
91_	hsa-mir-125b-2	MI0000470	hsa-miR-125b-5p	MIMAT0000423	hsa-miR-125b-2-3p	MIMAT0004603
92_	hsa-mir-126	MI0000471	hsa-miR-126-5p	MIMAT0000444	hsa-miR-126-3p	MIMAT0000445
93_	hsa-mir-1260a	MI0006394	hsa-miR-1260a-5p	MIMAT0005911	na	na
94_	hsa-mir-1260b	MI0014197	hsa-miR-1260b-5p	MIMAT0015041	na	na
95_	hsa-mir-1261	MI0006396	hsa-miR-1261-5p	MIMAT0005913	na	na
96_	hsa-mir-1262	MI0006397	hsa-miR-1262-5p	MIMAT0005914	na	na
97_	hsa-mir-1263	MI0006398	hsa-miR-1263-5p	MIMAT0005915	na	na
98_	hsa-mir-1264	MI0003758	na	na	hsa-miR-1264-3p	MIMAT0005791
99_	hsa-mir-1265	MI0006401	hsa-miR-1265-5p	MIMAT0005918	na	na
100_	hsa-mir-1266	MI0006403	hsa-miR-1266-5p	MIMAT0005920	hsa-miR-1266-3p	MIMAT0026742
101_	hsa-mir-1267	MI0006404	hsa-miR-1267-5p	MIMAT0005921	na	na
102_	hsa-mir-1268a	MI0006405	hsa-miR-1268a-5p	MIMAT0005922	na	na
103_	hsa-mir-1268b	MI0016748	hsa-miR-1268b-5p	MIMAT0018925	na	na
104_	hsa-mir-1269a	MI0006406	na	na	hsa-miR-1269a-3p	MIMAT0005923
105_	hsa-mir-1269b	MI0016888	hsa-miR-1269b-5p	MIMAT0019059	na	na
106_	hsa-mir-127	MI0000472	hsa-miR-127-5p	MIMAT0004604	hsa-miR-127-3p	MIMAT0000446
107_	hsa-mir-1270-1	MI0006407	hsa-miR-1270-5p	MIMAT0005924	na	na
108_	hsa-mir-1270-2	MI0015976	hsa-miR-1270-5p	MIMAT0005924	na	na
109_	hsa-mir-1271	MI0003814	hsa-miR-1271-5p	MIMAT0005796	hsa-miR-1271-3p	MIMAT0022712
110_	hsa-mir-1272	MI0006408	hsa-miR-1272-5p	MIMAT0005925	na	na
111_	hsa-mir-1273a	MI0006409	na	na	hsa-miR-1273a-3p	MIMAT0005926
112_	hsa-mir-1273c	MI0014171	hsa-miR-1273c-5p	MIMAT0015017	na	na
113_	hsa-mir-1273d	MI0014254	hsa-miR-1273d-5p	MIMAT0015090	na	na
114_	hsa-mir-1273e	MI0016059	hsa-miR-1273e-5p	MIMAT0018079	na	na
115_	hsa-mir-1273f	MI0018002	hsa-miR-1273f-5p	MIMAT0020601	na	na
116_	hsa-mir-1273g	MI0018003	hsa-miR-1273g-5p	MIMAT0020602	hsa-miR-1273g-3p	MIMAT0022742

117_	hsa-mir-1273h	MI0025512	hsa-miR-1273h-5p	MIMAT0030415	hsa-miR-1273h-3p	MIMAT0030416
118_	hsa-mir-1275	MI0006415	hsa-miR-1275-5p	MIMAT0005929	na	na
119_	hsa-mir-1276	MI0006416	hsa-miR-1276-5p	MIMAT0005930	na	na
120_	hsa-mir-1277	MI0006419	hsa-miR-1277-5p	MIMAT0022724	hsa-miR-1277-3p	MIMAT0005933
121_	hsa-mir-1278	MI0006425	na	na	hsa-miR-1278-3p	MIMAT0005936
122_	hsa-mir-1279	MI0006426	hsa-miR-1279-5p	MIMAT0005937	na	na
123_	hsa-mir-128-1	MI0000447	hsa-miR-128-1-5p	MIMAT0026477	hsa-miR-128-3p	MIMAT0000424
124_	hsa-mir-128-2	MI0000727	hsa-miR-128-2-5p	MIMAT0031095	hsa-miR-128-3p	MIMAT0000424
125_	hsa-mir-1281	MI0006428	na	na	hsa-miR-1281-3p	MIMAT0005939
126_	hsa-mir-1282	MI0006429	hsa-miR-1282-5p	MIMAT0005940	na	na
127_	hsa-mir-1283-1	MI0003832	hsa-miR-1283-5p	MIMAT0005799	na	na
128_	hsa-mir-1283-2	MI0006430	hsa-miR-1283-5p	MIMAT0005799	na	na
129_	hsa-mir-1284	MI0006431	hsa-miR-1284-5p	MIMAT0005941	na	na
130_	hsa-mir-1285-1	MI0006346	hsa-miR-1285-5p	MIMAT0022719	hsa-miR-1285-3p	MIMAT0005876
131_	hsa-mir-1285-2	MI0006347	na	na	hsa-miR-1285-3p	MIMAT0005876
132_	hsa-mir-1286	MI0006348	na	na	hsa-miR-1286-3p	MIMAT0005877
133_	hsa-mir-1287	MI0006349	hsa-miR-1287-5p	MIMAT0005878	hsa-miR-1287-3p	MIMAT0026738
134_	hsa-mir-1288	MI0006432	hsa-miR-1288-5p	MIMAT0026743	hsa-miR-1288-3p	MIMAT0005942
135_	hsa-mir-1289-1	MI0006350	na	na	hsa-miR-1289-3p	MIMAT0005879
136_	hsa-mir-1289-2	MI0006351	na	na	hsa-miR-1289-3p	MIMAT0005879
137_	hsa-mir-129-1	MI0000252	hsa-miR-129-5p	MIMAT0000242	hsa-miR-129-1-3p	MIMAT0004548
138_	hsa-mir-129-2	MI0000473	hsa-miR-129-5p	MIMAT0000242	hsa-miR-129-2-3p	MIMAT0004605
139_	hsa-mir-1290	MI0006352	na	na	hsa-miR-1290-3p	MIMAT0005880
140_	hsa-mir-1291	MI0006353	hsa-miR-1291-5p	MIMAT0005881	na	na
141_	hsa-mir-1292	MI0006433	hsa-miR-1292-5p	MIMAT0005943	hsa-miR-1292-3p	MIMAT0022948
142_	hsa-mir-1293	MI0006355	hsa-miR-1293-5p	MIMAT0005883	na	na
143_	hsa-mir-1294	MI0006356	hsa-miR-1294-5p	MIMAT0005884	na	na
144_	hsa-mir-1295a	MI0006357	na	na	hsa-miR-1295a-3p	MIMAT0005885
145_	hsa-mir-1295b	MI0019146	hsa-miR-1295b-5p	MIMAT0022293	hsa-miR-1295b-3p	MIMAT0022294

146_	hsa-mir-1296	MI0003780	hsa-miR-1296-5p	MIMAT0005794	hsa-miR-1296-3p	MIMAT0026637
147_	hsa-mir-1297	MI0006358	na	na	hsa-miR-1297-3p	MIMAT0005886
148_	hsa-mir-1298	MI0003938	hsa-miR-1298-5p	MIMAT0005800	hsa-miR-1298-3p	MIMAT0026641
149_	hsa-mir-1299	MI0006359	na	na	hsa-miR-1299-3p	MIMAT0005887
150_	hsa-mir-1301	MI0003815	hsa-miR-1301-5p	MIMAT0026639	hsa-miR-1301-3p	MIMAT0005797
151_	hsa-mir-1302-1	MI0006362	na	na	hsa-miR-1302-3p	MIMAT0005890
152_	hsa-mir-1302-10	MI0015979	na	na	hsa-miR-1302-3p	MIMAT0005890
153_	hsa-mir-1302-11	MI0015980	na	na	hsa-miR-1302-3p	MIMAT0005890
154_	hsa-mir-1302-2	MI0006363	na	na	hsa-miR-1302-3p	MIMAT0005890
155_	hsa-mir-1302-3	MI0006364	na	na	hsa-miR-1302-3p	MIMAT0005890
156_	hsa-mir-1302-4	MI0006365	na	na	hsa-miR-1302-3p	MIMAT0005890
157_	hsa-mir-1302-5	MI0006366	na	na	hsa-miR-1302-3p	MIMAT0005890
158_	hsa-mir-1302-6	MI0006367	na	na	hsa-miR-1302-3p	MIMAT0005890
159_	hsa-mir-1302-7	MI0006368	na	na	hsa-miR-1302-3p	MIMAT0005890
160_	hsa-mir-1302-8	MI0006369	na	na	hsa-miR-1302-3p	MIMAT0005890
161_	hsa-mir-1302-9	MI0015978	na	na	hsa-miR-1302-3p	MIMAT0005890
162_	hsa-mir-1303	MI0006370	na	na	hsa-miR-1303-3p	MIMAT0005891
163_	hsa-mir-1304	MI0006371	hsa-miR-1304-5p	MIMAT0005892	hsa-miR-1304-3p	MIMAT0022720
164_	hsa-mir-1305	MI0006372	na	na	hsa-miR-1305-3p	MIMAT0005893
165_	hsa-mir-1306	MI0006443	hsa-miR-1306-5p	MIMAT0022726	hsa-miR-1306-3p	MIMAT0005950
166_	hsa-mir-1307	MI0006444	hsa-miR-1307-5p	MIMAT0022727	hsa-miR-1307-3p	MIMAT0005951
167_	hsa-mir-130a	MI0000448	hsa-miR-130a-5p	MIMAT0004593	hsa-miR-130a-3p	MIMAT0000425
168_	hsa-mir-130b	MI0000748	hsa-miR-130b-5p	MIMAT0004680	hsa-miR-130b-3p	MIMAT0000691
169_	hsa-mir-132	MI0000449	hsa-miR-132-5p	MIMAT0004594	hsa-miR-132-3p	MIMAT0000426
170_	hsa-mir-1321	MI0006652	na	na	hsa-miR-1321-3p	MIMAT0005952
171_	hsa-mir-1322	MI0006653	na	na	hsa-miR-1322-3p	MIMAT0005953
172_	hsa-mir-1323	MI0003786	hsa-miR-1323-5p	MIMAT0005795	na	na
173_	hsa-mir-1324	MI0006657	na	na	hsa-miR-1324-3p	MIMAT0005956
174_	hsa-mir-133a-1	MI0000450	hsa-miR-133a-5p	MIMAT0026478	hsa-miR-133a-3p	MIMAT0000427

175_	hsa-mir-133a-2	MI0000451	hsa-miR-133a-5p	MIMAT0026478	hsa-miR-133a-3p	MIMAT0000427
176_	hsa-mir-133b	MI0000822	na	na	hsa-miR-133b-3p	MIMAT0000770
177_	hsa-mir-134	MI0000474	hsa-miR-134-5p	MIMAT0000447	hsa-miR-134-3p	MIMAT0026481
178_	hsa-mir-1343	MI0017320	hsa-miR-1343-5p	MIMAT0027038	hsa-miR-1343-3p	MIMAT0019776
179_	hsa-mir-135a-1	MI0000452	hsa-miR-135a-5p	MIMAT0000428	hsa-miR-135a-3p	MIMAT0004595
180_	hsa-mir-135a-2	MI0000453	hsa-miR-135a-5p	MIMAT0000428	na	na
181_	hsa-mir-135b	MI0000810	hsa-miR-135b-5p	MIMAT0000758	hsa-miR-135b-3p	MIMAT0004698
182_	hsa-mir-136	MI0000475	hsa-miR-136-5p	MIMAT0000448	hsa-miR-136-3p	MIMAT0004606
183_	hsa-mir-137	MI0000454	na	na	hsa-miR-137-3p	MIMAT0000429
184_	hsa-mir-138-1	MI0000476	hsa-miR-138-5p	MIMAT0000430	hsa-miR-138-1-3p	MIMAT0004607
185_	hsa-mir-138-2	MI0000455	hsa-miR-138-5p	MIMAT0000430	hsa-miR-138-2-3p	MIMAT0004596
186_	hsa-mir-139	MI0000261	hsa-miR-139-5p	MIMAT0000250	hsa-miR-139-3p	MIMAT0004552
187_	hsa-mir-140	MI0000456	hsa-miR-140-5p	MIMAT0000431	hsa-miR-140-3p	MIMAT0004597
188_	hsa-mir-141	MI0000457	hsa-miR-141-5p	MIMAT0004598	hsa-miR-141-3p	MIMAT0000432
189_	hsa-mir-142	MI0000458	hsa-miR-142-5p	MIMAT0000433	hsa-miR-142-3p	MIMAT0000434
190_	hsa-mir-143	MI0000459	hsa-miR-143-5p	MIMAT0004599	hsa-miR-143-3p	MIMAT0000435
191_	hsa-mir-144	MI0000460	hsa-miR-144-5p	MIMAT0004600	hsa-miR-144-3p	MIMAT0000436
192_	hsa-mir-145	MI0000461	hsa-miR-145-5p	MIMAT0000437	hsa-miR-145-3p	MIMAT0004601
193_	hsa-mir-1468	MI0003782	hsa-miR-1468-5p	MIMAT0006789	hsa-miR-1468-3p	MIMAT0026638
194_	hsa-mir-1469	MI0007074	hsa-miR-1469-5p	MIMAT0007347	na	na
195_	hsa-mir-146a	MI0000477	hsa-miR-146a-5p	MIMAT0000449	hsa-miR-146a-3p	MIMAT0004608
196_	hsa-mir-146b	MI0003129	hsa-miR-146b-5p	MIMAT0002809	hsa-miR-146b-3p	MIMAT0004766
197_	hsa-mir-1470	MI0007075	hsa-miR-1470-5p	MIMAT0007348	na	na
198_	hsa-mir-1471	MI0007076	hsa-miR-1471-5p	MIMAT0007349	na	na
199_	hsa-mir-147a	MI0000262	na	na	hsa-miR-147a-3p	MIMAT0000251
200_	hsa-mir-147b	MI0005544	na	na	hsa-miR-147b-3p	MIMAT0004928
201_	hsa-mir-148a	MI0000253	hsa-miR-148a-5p	MIMAT0004549	hsa-miR-148a-3p	MIMAT0000243
202_	hsa-mir-148b	MI0000811	hsa-miR-148b-5p	MIMAT0004699	hsa-miR-148b-3p	MIMAT0000759
203_	hsa-mir-149	MI0000478	hsa-miR-149-5p	MIMAT0000450	hsa-miR-149-3p	MIMAT0004609

204_	hsa-mir-150	MI0000479	hsa-miR-150-5p	MIMAT0000451	hsa-miR-150-3p	MIMAT0004610
205_	hsa-mir-151a	MI0000809	hsa-miR-151a-5p	MIMAT0004697	hsa-miR-151a-3p	MIMAT0000757
206_	hsa-mir-151b	MI0003772	na	na	hsa-miR-151b-3p	MIMAT0010214
207_	hsa-mir-152	MI0000462	hsa-miR-152-5p	MIMAT0026479	hsa-miR-152-3p	MIMAT0000438
208_	hsa-mir-153-1	MI0000463	na	na	hsa-miR-153-3p	MIMAT0000439
209_	hsa-mir-153-2	MI0000464	hsa-miR-153-5p	MIMAT0026480	hsa-miR-153-3p	MIMAT0000439
210_	hsa-mir-1537	MI0007258	hsa-miR-1537-5p	MIMAT0026765	hsa-miR-1537-3p	MIMAT0007399
211_	hsa-mir-1538	MI0007259	na	na	hsa-miR-1538-3p	MIMAT0007400
212_	hsa-mir-1539	MI0007260	na	na	hsa-miR-1539-3p	MIMAT0007401
213_	hsa-mir-154	MI0000480	hsa-miR-154-5p	MIMAT0000452	hsa-miR-154-3p	MIMAT0000453
214_	hsa-mir-155	MI0000681	hsa-miR-155-5p	MIMAT0000646	hsa-miR-155-3p	MIMAT0004658
215_	hsa-mir-1587	MI0016905	hsa-miR-1587-5p	MIMAT0019077	na	na
216_	hsa-mir-15a	MI0000069	hsa-miR-15a-5p	MIMAT0000068	hsa-miR-15a-3p	MIMAT0004488
217_	hsa-mir-15b	MI0000438	hsa-miR-15b-5p	MIMAT0000417	hsa-miR-15b-3p	MIMAT0004586
218_	hsa-mir-16-1	MI0000070	hsa-miR-16-5p	MIMAT0000069	hsa-miR-16-1-3p	MIMAT0004489
219_	hsa-mir-16-2	MI0000115	hsa-miR-16-5p	MIMAT0000069	hsa-miR-16-2-3p	MIMAT0004518
220_	hsa-mir-17	MI0000071	hsa-miR-17-5p	MIMAT0000070	hsa-miR-17-3p	MIMAT0000071
221_	hsa-mir-181a-1	MI0000289	hsa-miR-181a-5p	MIMAT0000256	hsa-miR-181a-3p	MIMAT0000270
222_	hsa-mir-181a-2	MI0000269	hsa-miR-181a-5p	MIMAT0000256	hsa-miR-181a-2-3p	MIMAT0004558
223_	hsa-mir-181b-1	MI0000270	hsa-miR-181b-5p	MIMAT0000257	hsa-miR-181b-3p	MIMAT0022692
224_	hsa-mir-181b-2	MI0000683	hsa-miR-181b-5p	MIMAT0000257	na	na
225_	hsa-mir-181c	MI0000271	hsa-miR-181c-5p	MIMAT0000258	hsa-miR-181c-3p	MIMAT0004559
226_	hsa-mir-181d	MI0003139	hsa-miR-181d-5p	MIMAT0002821	hsa-miR-181d-3p	MIMAT0026608
227_	hsa-mir-182	MI0000272	hsa-miR-182-5p	MIMAT0000259	hsa-miR-182-3p	MIMAT0000260
228_	hsa-mir-1825	MI0008193	na	na	hsa-miR-1825-3p	MIMAT0006765
229_	hsa-mir-1827	MI0008195	na	na	hsa-miR-1827-3p	MIMAT0006767
230_	hsa-mir-183	MI0000273	hsa-miR-183-5p	MIMAT0000261	hsa-miR-183-3p	MIMAT0004560
231_	hsa-mir-184	MI0000481	na	na	hsa-miR-184-3p	MIMAT0000454
232_	hsa-mir-185	MI0000482	hsa-miR-185-5p	MIMAT0000455	hsa-miR-185-3p	MIMAT0004611

233_	hsa-mir-186	MI0000483	hsa-miR-186-5p	MIMAT0000456	hsa-miR-186-3p	MIMAT0004612
234_	hsa-mir-187	MI0000274	hsa-miR-187-5p	MIMAT0004561	hsa-miR-187-3p	MIMAT0000262
235_	hsa-mir-188	MI0000484	hsa-miR-188-5p	MIMAT0000457	hsa-miR-188-3p	MIMAT0004613
236_	hsa-mir-18a	MI0000072	hsa-miR-18a-5p	MIMAT0000072	hsa-miR-18a-3p	MIMAT0002891
237_	hsa-mir-18b	MI0001518	hsa-miR-18b-5p	MIMAT0001412	hsa-miR-18b-3p	MIMAT0004751
238_	hsa-mir-1908	MI0008329	hsa-miR-1908-5p	MIMAT0007881	hsa-miR-1908-3p	MIMAT0026916
239_	hsa-mir-1909	MI0008330	hsa-miR-1909-5p	MIMAT0007882	hsa-miR-1909-3p	MIMAT0007883
240_	hsa-mir-190a	MI0000486	hsa-miR-190a-5p	MIMAT0000458	hsa-miR-190a-3p	MIMAT0026482
241_	hsa-mir-190b	MI0005545	hsa-miR-190b-5p	MIMAT0004929	na	na
242_	hsa-mir-191	MI0000465	hsa-miR-191-5p	MIMAT0000440	hsa-miR-191-3p	MIMAT0001618
243_	hsa-mir-1910	MI0008331	hsa-miR-1910-5p	MIMAT0007884	hsa-miR-1910-3p	MIMAT0026917
244_	hsa-mir-1911	MI0008332	hsa-miR-1911-5p	MIMAT0007885	hsa-miR-1911-3p	MIMAT0007886
245_	hsa-mir-1912	MI0008333	na	na	hsa-miR-1912-3p	MIMAT0007887
246_	hsa-mir-1913	MI0008334	na	na	hsa-miR-1913-3p	MIMAT0007888
247_	hsa-mir-1914	MI0008335	hsa-miR-1914-5p	MIMAT0007889	hsa-miR-1914-3p	MIMAT0007890
248_	hsa-mir-1915	MI0008336	hsa-miR-1915-5p	MIMAT0007891	hsa-miR-1915-3p	MIMAT0007892
249_	hsa-mir-192	MI0000234	hsa-miR-192-5p	MIMAT0000222	hsa-miR-192-3p	MIMAT0004543
250_	hsa-mir-193a	MI0000487	hsa-miR-193a-5p	MIMAT0004614	hsa-miR-193a-3p	MIMAT0000459
251_	hsa-mir-193b	MI0003137	hsa-miR-193b-5p	MIMAT0004767	hsa-miR-193b-3p	MIMAT0002819
252_	hsa-mir-194-1	MI0000488	hsa-miR-194-5p	MIMAT0000460	na	na
253_	hsa-mir-194-2	MI0000732	hsa-miR-194-5p	MIMAT0000460	hsa-miR-194-3p	MIMAT0004671
254_	hsa-mir-195	MI0000489	hsa-miR-195-5p	MIMAT0000461	hsa-miR-195-3p	MIMAT0004615
255_	hsa-mir-196a-1	MI0000238	hsa-miR-196a-5p	MIMAT0000226	na	na
256_	hsa-mir-196a-2	MI0000279	hsa-miR-196a-5p	MIMAT0000226	hsa-miR-196a-3p	MIMAT0004562
257_	hsa-mir-196b	MI0001150	hsa-miR-196b-5p	MIMAT0001080	hsa-miR-196b-3p	MIMAT0009201
258_	hsa-mir-197	MI0000239	hsa-miR-197-5p	MIMAT0022691	hsa-miR-197-3p	MIMAT0000227
259_	hsa-mir-1972-1	MI0009982	na	na	hsa-miR-1972-3p	MIMAT0009447
260_	hsa-mir-1972-2	MI0015977	na	na	hsa-miR-1972-3p	MIMAT0009447

261_	hsa-mir-1973	MI0009983	na	na	hsa-miR-1973-3p	MIMAT0009448
262_	hsa-mir-1976	MI0009986	na	na	hsa-miR-1976-3p	MIMAT0009451
263_	hsa-mir-198	MI0000240	hsa-miR-198-5p	MIMAT0000228	na	na
264_	hsa-mir-199a-1	MI0000242	hsa-miR-199a-5p	MIMAT0000231	hsa-miR-199a-3p	MIMAT0000232
265_	hsa-mir-199a-2	MI0000281	hsa-miR-199a-5p	MIMAT0000231	hsa-miR-199a-3p	MIMAT0000232
266_	hsa-mir-199b	MI0000282	hsa-miR-199b-5p	MIMAT0000263	hsa-miR-199b-3p	MIMAT0004563
267_	hsa-mir-19a	MI0000073	hsa-miR-19a-5p	MIMAT0004490	hsa-miR-19a-3p	MIMAT0000073
268_	hsa-mir-19b-1	MI0000074	hsa-miR-19b-1-5p	MIMAT0004491	hsa-miR-19b-3p	MIMAT0000074
269_	hsa-mir-19b-2	MI0000075	hsa-miR-19b-2-5p	MIMAT0004492	hsa-miR-19b-3p	MIMAT0000074
270_	hsa-mir-200a	MI0000737	hsa-miR-200a-5p	MIMAT0001620	hsa-miR-200a-3p	MIMAT0000682
271_	hsa-mir-200b	MI0000342	hsa-miR-200b-5p	MIMAT0004571	hsa-miR-200b-3p	MIMAT0000318
272_	hsa-mir-200c	MI0000650	hsa-miR-200c-5p	MIMAT0004657	hsa-miR-200c-3p	MIMAT0000617
273_	hsa-mir-202	MI0003130	hsa-miR-202-5p	MIMAT0002810	hsa-miR-202-3p	MIMAT0002811
274_	hsa-mir-203a	MI0000283	na	na	hsa-miR-203a-3p	MIMAT0000264
275_	hsa-mir-203b	MI0017343	hsa-miR-203b-5p	MIMAT0019813	hsa-miR-203b-3p	MIMAT0019814
276_	hsa-mir-204	MI0000284	hsa-miR-204-5p	MIMAT0000265	hsa-miR-204-3p	MIMAT0022693
277_	hsa-mir-205	MI0000285	hsa-miR-205-5p	MIMAT0000266	hsa-miR-205-3p	MIMAT0009197
278_	hsa-mir-2052	MI0010486	hsa-miR-2052-5p	MIMAT0009977	na	na
279_	hsa-mir-2053	MI0010487	na	na	hsa-miR-2053-3p	MIMAT0009978
280_	hsa-mir-2054	MI0010488	hsa-miR-2054-5p	MIMAT0009979	na	na
281_	hsa-mir-206	MI0000490	na	na	hsa-miR-206-3p	MIMAT0000462
282_	hsa-mir-208a	MI0000251	hsa-miR-208a-5p	MIMAT0026474	hsa-miR-208a-3p	MIMAT0000241
283_	hsa-mir-208b	MI0005570	hsa-miR-208b-5p	MIMAT0026722	hsa-miR-208b-3p	MIMAT0004960
284_	hsa-mir-20a	MI0000076	hsa-miR-20a-5p	MIMAT0000075	hsa-miR-20a-3p	MIMAT0004493
285_	hsa-mir-20b	MI0001519	hsa-miR-20b-5p	MIMAT0001413	hsa-miR-20b-3p	MIMAT0004752
286_	hsa-mir-21	MI0000077	hsa-miR-21-5p	MIMAT0000076	hsa-miR-21-3p	MIMAT0004494
287_	hsa-mir-210	MI0000286	hsa-miR-210-5p	MIMAT0026475	hsa-miR-210-3p	MIMAT0000267
288_	hsa-mir-211	MI0000287	hsa-miR-211-5p	MIMAT0000268	hsa-miR-211-3p	MIMAT0022694

289_	hsa-mir-2110	MI0010629	hsa-miR-2110-5p	MIMAT0010133	na	na
290_	hsa-mir-2113	MI0003939	na	na	hsa-miR-2113-3p	MIMAT0009206
291_	hsa-mir-2114	MI0010633	hsa-miR-2114-5p	MIMAT0011156	hsa-miR-2114-3p	MIMAT0011157
292_	hsa-mir-2115	MI0010634	hsa-miR-2115-5p	MIMAT0011158	hsa-miR-2115-3p	MIMAT0011159
293_	hsa-mir-2116	MI0010635	hsa-miR-2116-5p	MIMAT0011160	hsa-miR-2116-3p	MIMAT0011161
294_	hsa-mir-2117	MI0010636	na	na	hsa-miR-2117-3p	MIMAT0011162
295_	hsa-mir-212	MI0000288	hsa-miR-212-5p	MIMAT0022695	hsa-miR-212-3p	MIMAT0000269
296_	hsa-mir-214	MI0000290	hsa-miR-214-5p	MIMAT0004564	hsa-miR-214-3p	MIMAT0000271
297_	hsa-mir-215	MI0000291	hsa-miR-215-5p	MIMAT0000272	hsa-miR-215-3p	MIMAT0026476
298_	hsa-mir-216a	MI0000292	hsa-miR-216a-5p	MIMAT0000273	hsa-miR-216a-3p	MIMAT0022844
299_	hsa-mir-216b	MI0005569	hsa-miR-216b-5p	MIMAT0004959	hsa-miR-216b-3p	MIMAT0026721
300_	hsa-mir-217	MI0000293	hsa-miR-217-5p	MIMAT0000274	na	na
301_	hsa-mir-218-1	MI0000294	hsa-miR-218-5p	MIMAT0000275	hsa-miR-218-1-3p	MIMAT0004565
302_	hsa-mir-218-2	MI0000295	hsa-miR-218-5p	MIMAT0000275	hsa-miR-218-2-3p	MIMAT0004566
303_	hsa-mir-219a-1	MI0000296	hsa-miR-219a-5p	MIMAT0000276	hsa-miR-219a-1-3p	MIMAT0004567
304_	hsa-mir-219a-2	MI0000740	hsa-miR-219a-5p	MIMAT0000276	hsa-miR-219a-2-3p	MIMAT0004675
305_	hsa-mir-219b	MI0017299	hsa-miR-219b-5p	MIMAT0019747	hsa-miR-219b-3p	MIMAT0019748
306_	hsa-mir-22	MI0000078	hsa-miR-22-5p	MIMAT0004495	hsa-miR-22-3p	MIMAT0000077
307_	hsa-mir-221	MI0000298	hsa-miR-221-5p	MIMAT0004568	hsa-miR-221-3p	MIMAT0000278
308_	hsa-mir-222	MI0000299	hsa-miR-222-5p	MIMAT0004569	hsa-miR-222-3p	MIMAT0000279
309_	hsa-mir-223	MI0000300	hsa-miR-223-5p	MIMAT0004570	hsa-miR-223-3p	MIMAT0000280
310_	hsa-mir-224	MI0000301	hsa-miR-224-5p	MIMAT0000281	hsa-miR-224-3p	MIMAT0009198
311_	hsa-mir-2276	MI0011282	hsa-miR-2276-5p	MIMAT0026921	hsa-miR-2276-3p	MIMAT0011775
312_	hsa-mir-2277	MI0011284	hsa-miR-2277-5p	MIMAT0017352	hsa-miR-2277-3p	MIMAT0011777
313_	hsa-mir-2278	MI0011285	hsa-miR-2278-5p	MIMAT0011778	na	na
314_	hsa-mir-2355	MI0015873	hsa-miR-2355-5p	MIMAT0016895	hsa-miR-2355-3p	MIMAT0017950
315_	hsa-mir-2392	MI0016870	na	na	hsa-miR-2392-3p	MIMAT0019043
316_	hsa-mir-23a	MI0000079	hsa-miR-23a-5p	MIMAT0004496	hsa-miR-23a-3p	MIMAT0000078
317_	hsa-mir-23b	MI0000439	hsa-miR-23b-5p	MIMAT0004587	hsa-miR-23b-3p	MIMAT0000418

318_	hsa-mir-23c	MI0016010	na	na	hsa-miR-23c-3p	MIMAT0018000
319_	hsa-mir-24-1	MI0000080	hsa-miR-24-1-5p	MIMAT0000079	hsa-miR-24-3p	MIMAT0000080
320_	hsa-mir-24-2	MI0000081	hsa-miR-24-2-5p	MIMAT0004497	hsa-miR-24-3p	MIMAT0000080
321_	hsa-mir-2467	MI0017432	hsa-miR-2467-5p	MIMAT0019952	hsa-miR-2467-3p	MIMAT0019953
322_	hsa-mir-25	MI0000082	hsa-miR-25-5p	MIMAT0004498	hsa-miR-25-3p	MIMAT0000081
323_	hsa-mir-2681	MI0012062	hsa-miR-2681-5p	MIMAT0013515	hsa-miR-2681-3p	MIMAT0013516
324_	hsa-mir-2682	MI0012063	hsa-miR-2682-5p	MIMAT0013517	hsa-miR-2682-3p	MIMAT0013518
325_	hsa-mir-26a-1	MI0000083	hsa-miR-26a-5p	MIMAT0000082	hsa-miR-26a-1-3p	MIMAT0004499
326_	hsa-mir-26a-2	MI0000750	hsa-miR-26a-5p	MIMAT0000082	hsa-miR-26a-2-3p	MIMAT0004681
327_	hsa-mir-26b	MI0000084	hsa-miR-26b-5p	MIMAT0000083	hsa-miR-26b-3p	MIMAT0004500
328_	hsa-mir-27a	MI0000085	hsa-miR-27a-5p	MIMAT0004501	hsa-miR-27a-3p	MIMAT0000084
329_	hsa-mir-27b	MI0000440	hsa-miR-27b-5p	MIMAT0004588	hsa-miR-27b-3p	MIMAT0000419
330_	hsa-mir-28	MI0000086	hsa-miR-28-5p	MIMAT0000085	hsa-miR-28-3p	MIMAT0004502
331_	hsa-mir-2861	MI0013006	na	na	hsa-miR-2861-3p	MIMAT0013802
332_	hsa-mir-2909	MI0013083	hsa-miR-2909-5p	MIMAT0013863	na	na
333_	hsa-mir-296	MI0000747	hsa-miR-296-5p	MIMAT0000690	hsa-miR-296-3p	MIMAT0004679
334_	hsa-mir-297	MI0005775	hsa-miR-297-5p	MIMAT0004450	na	na
335_	hsa-mir-298	MI0005523	hsa-miR-298-5p	MIMAT0004901	na	na
336_	hsa-mir-299	MI0000744	hsa-miR-299-5p	MIMAT0002890	hsa-miR-299-3p	MIMAT0000687
337_	hsa-mir-29a	MI0000087	hsa-miR-29a-5p	MIMAT0004503	hsa-miR-29a-3p	MIMAT0000086
338_	hsa-mir-29b-1	MI0000105	hsa-miR-29b-1-5p	MIMAT0004514	hsa-miR-29b-3p	MIMAT0000100
339_	hsa-mir-29b-2	MI0000107	hsa-miR-29b-2-5p	MIMAT0004515	hsa-miR-29b-3p	MIMAT0000100
340_	hsa-mir-29c	MI0000735	hsa-miR-29c-5p	MIMAT0004673	hsa-miR-29c-3p	MIMAT0000681
341_	hsa-mir-300	MI0005525	na	na	hsa-miR-300-3p	MIMAT0004903
342_	hsa-mir-301a	MI0000745	hsa-miR-301a-5p	MIMAT0022696	hsa-miR-301a-3p	MIMAT0000688
343_	hsa-mir-301b	MI0005568	na	na	hsa-miR-301b-3p	MIMAT0004958
344_	hsa-mir-302a	MI0000738	hsa-miR-302a-5p	MIMAT0000683	hsa-miR-302a-3p	MIMAT0000684
345_	hsa-mir-302b	MI0000772	hsa-miR-302b-5p	MIMAT0000714	hsa-miR-302b-3p	MIMAT0000715
346_	hsa-mir-302c	MI0000773	hsa-miR-302c-5p	MIMAT0000716	hsa-miR-302c-3p	MIMAT0000717

347_	hsa-mir-302d	MI0000774	hsa-miR-302d-5p	MIMAT0004685	hsa-miR-302d-3p	MIMAT0000718
348_	hsa-mir-302e	MI0006417	hsa-miR-302e-5p	MIMAT0005931	na	na
349_	hsa-mir-302f	MI0006418	na	na	hsa-miR-302f-3p	MIMAT0005932
350_	hsa-mir-3064	MI0017375	hsa-miR-3064-5p	MIMAT0019864	hsa-miR-3064-3p	MIMAT0019865
351_	hsa-mir-3065	MI0014228	hsa-miR-3065-5p	MIMAT0015066	hsa-miR-3065-3p	MIMAT0015378
352_	hsa-mir-3074	MI0014181	hsa-miR-3074-5p	MIMAT0019208	hsa-miR-3074-3p	MIMAT0015027
353_	hsa-mir-30a	MI0000088	hsa-miR-30a-5p	MIMAT0000087	hsa-miR-30a-3p	MIMAT0000088
354_	hsa-mir-30b	MI0000441	hsa-miR-30b-5p	MIMAT0000420	hsa-miR-30b-3p	MIMAT0004589
355_	hsa-mir-30c-1	MI0000736	hsa-miR-30c-5p	MIMAT0000244	hsa-miR-30c-1-3p	MIMAT0004674
356_	hsa-mir-30c-2	MI0000254	hsa-miR-30c-5p	MIMAT0000244	hsa-miR-30c-2-3p	MIMAT0004550
357_	hsa-mir-30d	MI0000255	hsa-miR-30d-5p	MIMAT0000245	hsa-miR-30d-3p	MIMAT0004551
358_	hsa-mir-30e	MI0000749	hsa-miR-30e-5p	MIMAT0000692	hsa-miR-30e-3p	MIMAT0000693
359_	hsa-mir-31	MI0000089	hsa-miR-31-5p	MIMAT0000089	hsa-miR-31-3p	MIMAT0004504
360_	hsa-mir-3115	MI0014127	hsa-miR-3115-5p	MIMAT0014977	na	na
361_	hsa-mir-3116-1	MI0014128	na	na	hsa-miR-3116-3p	MIMAT0014978
362_	hsa-mir-3116-2	MI0014129	na	na	hsa-miR-3116-3p	MIMAT0014978
363_	hsa-mir-3117	MI0014130	hsa-miR-3117-5p	MIMAT0019197	hsa-miR-3117-3p	MIMAT0014979
364_	hsa-mir-3118-1	MI0014131	na	na	hsa-miR-3118-3p	MIMAT0014980
365_	hsa-mir-3118-2	MI0014132	na	na	hsa-miR-3118-3p	MIMAT0014980
366_	hsa-mir-3118-3	MI0014133	na	na	hsa-miR-3118-3p	MIMAT0014980
367_	hsa-mir-3118-4	MI0014207	na	na	hsa-miR-3118-3p	MIMAT0014980
368_	hsa-mir-3118-5	MI0014243	na	na	hsa-miR-3118-3p	MIMAT0014980
369_	hsa-mir-3118-6	MI0015981	na	na	hsa-miR-3118-3p	MIMAT0014980
370_	hsa-mir-3119-1	MI0014134	hsa-miR-3119-5p	MIMAT0014981	na	na
371_	hsa-mir-3119-2	MI0014135	hsa-miR-3119-5p	MIMAT0014981	na	na
372_	hsa-mir-3120	MI0014136	hsa-miR-3120-5p	MIMAT0019198	hsa-miR-3120-3p	MIMAT0014982
373_	hsa-mir-3121	MI0014137	hsa-miR-3121-5p	MIMAT0019199	hsa-miR-3121-3p	MIMAT0014983
374_	hsa-mir-3122	MI0014138	hsa-miR-3122-5p	MIMAT0014984	na	na
375_	hsa-mir-3123	MI0014139	na	na	hsa-miR-3123-3p	MIMAT0014985

376_	hsa-mir-3124	MI0014140	hsa-miR-3124-5p	MIMAT0014986	hsa-miR-3124-3p	MIMAT0019200
377_	hsa-mir-3125	MI0014142	hsa-miR-3125-5p	MIMAT0014988	na	na
378_	hsa-mir-3126	MI0014143	hsa-miR-3126-5p	MIMAT0014989	hsa-miR-3126-3p	MIMAT0015377
379_	hsa-mir-3127	MI0014144	hsa-miR-3127-5p	MIMAT0014990	hsa-miR-3127-3p	MIMAT0019201
380_	hsa-mir-3128	MI0014145	hsa-miR-3128-5p	MIMAT0014991	na	na
381_	hsa-mir-3129	MI0014146	hsa-miR-3129-5p	MIMAT0014992	hsa-miR-3129-3p	MIMAT0019202
382_	hsa-mir-3130-1	MI0014147	hsa-miR-3130-5p	MIMAT0014995	hsa-miR-3130-3p	MIMAT0014994
383_	hsa-mir-3130-2	MI0014148	hsa-miR-3130-5p	MIMAT0014995	hsa-miR-3130-3p	MIMAT0014994
384_	hsa-mir-3131	MI0014151	hsa-miR-3131-5p	MIMAT0014996	na	na
385_	hsa-mir-3132	MI0014152	hsa-miR-3132-5p	MIMAT0014997	na	na
386_	hsa-mir-3133	MI0014153	hsa-miR-3133-5p	MIMAT0014998	na	na
387_	hsa-mir-3134	MI0014155	na	na	hsa-miR-3134-3p	MIMAT0015000
388_	hsa-mir-3135a	MI0014156	hsa-miR-3135a-5p	MIMAT0015001	na	na
389_	hsa-mir-3135b	MI0016809	hsa-miR-3135b-5p	MIMAT0018985	na	na
390_	hsa-mir-3136	MI0014158	hsa-miR-3136-5p	MIMAT0015003	hsa-miR-3136-3p	MIMAT0019203
391_	hsa-mir-3137	MI0014160	hsa-miR-3137-5p	MIMAT0015005	na	na
392_	hsa-mir-3138	MI0014161	na	na	hsa-miR-3138-3p	MIMAT0015006
393_	hsa-mir-3139	MI0014162	hsa-miR-3139-5p	MIMAT0015007	na	na
394_	hsa-mir-3140	MI0014163	hsa-miR-3140-5p	MIMAT0019204	hsa-miR-3140-3p	MIMAT0015008
395_	hsa-mir-3141	MI0014165	hsa-miR-3141-5p	MIMAT0015010	na	na
396_	hsa-mir-3142	MI0014166	hsa-miR-3142-5p	MIMAT0015011	na	na
397_	hsa-mir-3143	MI0014167	hsa-miR-3143-5p	MIMAT0015012	na	na
398_	hsa-mir-3144	MI0014169	hsa-miR-3144-5p	MIMAT0015014	hsa-miR-3144-3p	MIMAT0015015
399_	hsa-mir-3145	MI0014170	hsa-miR-3145-5p	MIMAT0019205	hsa-miR-3145-3p	MIMAT0015016
400_	hsa-mir-3146	MI0014172	na	na	hsa-miR-3146-3p	MIMAT0015018
401_	hsa-mir-3147	MI0014173	hsa-miR-3147-5p	MIMAT0015019	na	na
402_	hsa-mir-3148	MI0014175	hsa-miR-3148-5p	MIMAT0015021	na	na
403_	hsa-mir-3149	MI0014176	na	na	hsa-miR-3149-3p	MIMAT0015022
404_	hsa-mir-3150a	MI0014177	hsa-miR-3150a-5p	MIMAT0019206	hsa-miR-3150a-3p	MIMAT0015023
405_	hsa-mir-3150b	MI0016426	hsa-miR-3150b-5p	MIMAT0019226	hsa-miR-3150b-3p	MIMAT0018194

406_	hsa-mir-3151	MI0014178	hsa-miR-3151-5p	MIMAT0015024	hsa-miR-3151-3p	MIMAT0027026
407_	hsa-mir-3152	MI0014179	hsa-miR-3152-5p	MIMAT0019207	hsa-miR-3152-3p	MIMAT0015025
408_	hsa-mir-3153	MI0014180	na	na	hsa-miR-3153-3p	MIMAT0015026
409_	hsa-mir-3154	MI0014182	na	na	hsa-miR-3154-3p	MIMAT0015028
410_	hsa-mir-3155a	MI0014183	na	na	hsa-miR-3155a-3p	MIMAT0015029
411_	hsa-mir-3155b	MI0016839	na	na	hsa-miR-3155b-3p	MIMAT0019012
412_	hsa-mir-3156-1	MI0014184	hsa-miR-3156-5p	MIMAT0015030	hsa-miR-3156-3p	MIMAT0019209
413_	hsa-mir-3156-2	MI0014230	hsa-miR-3156-5p	MIMAT0015030	hsa-miR-3156-3p	MIMAT0019209
414_	hsa-mir-3156-3	MI0014242	hsa-miR-3156-5p	MIMAT0015030	na	na
415_	hsa-mir-3157	MI0014185	hsa-miR-3157-5p	MIMAT0015031	hsa-miR-3157-3p	MIMAT0019210
416_	hsa-mir-3158-1	MI0014186	hsa-miR-3158-5p	MIMAT0019211	hsa-miR-3158-3p	MIMAT0015032
417_	hsa-mir-3158-2	MI0014187	hsa-miR-3158-5p	MIMAT0019211	hsa-miR-3158-3p	MIMAT0015032
418_	hsa-mir-3159	MI0014188	hsa-miR-3159-5p	MIMAT0015033	na	na
419_	hsa-mir-3160-1	MI0014189	hsa-miR-3160-5p	MIMAT0019212	hsa-miR-3160-3p	MIMAT0015034
420_	hsa-mir-3160-2	MI0014190	hsa-miR-3160-5p	MIMAT0019212	hsa-miR-3160-3p	MIMAT0015034
421_	hsa-mir-3161	MI0014191	hsa-miR-3161-5p	MIMAT0015035	na	na
422_	hsa-mir-3162	MI0014192	hsa-miR-3162-5p	MIMAT0015036	hsa-miR-3162-3p	MIMAT0019213
423_	hsa-mir-3163	MI0014193	hsa-miR-3163-5p	MIMAT0015037	na	na
424_	hsa-mir-3164	MI0014194	hsa-miR-3164-5p	MIMAT0015038	na	na
425_	hsa-mir-3165	MI0014195	hsa-miR-3165-5p	MIMAT0015039	na	na
426_	hsa-mir-3166	MI0014196	na	na	hsa-miR-3166-3p	MIMAT0015040
427_	hsa-mir-3167	MI0014198	na	na	hsa-miR-3167-3p	MIMAT0015042
428_	hsa-mir-3168	MI0014199	hsa-miR-3168-5p	MIMAT0015043	na	na
429_	hsa-mir-3169	MI0014200	hsa-miR-3169-5p	MIMAT0015044	na	na
430_	hsa-mir-3170	MI0014201	hsa-miR-3170-5p	MIMAT0015045	na	na
431_	hsa-mir-3171	MI0014202	hsa-miR-3171-5p	MIMAT0015046	na	na
432_	hsa-mir-3173	MI0014204	hsa-miR-3173-5p	MIMAT0019214	hsa-miR-3173-3p	MIMAT0015048
433_	hsa-mir-3174	MI0014208	hsa-miR-3174-5p	MIMAT0015051	na	na

434_	hsa-mir-3175	MI0014209	hsa-miR-3175-5p	MIMAT0015052	na	na
435_	hsa-mir-3176	MI0014210	na	na	hsa-miR-3176-3p	MIMAT0015053
436_	hsa-mir-3177	MI0014211	hsa-miR-3177-5p	MIMAT0019215	hsa-miR-3177-3p	MIMAT0015054
437_	hsa-mir-3178	MI0014212	hsa-miR-3178-5p	MIMAT0015055	na	na
438_	hsa-mir-3179-1	MI0014213	na	na	hsa-miR-3179-3p	MIMAT0015056
439_	hsa-mir-3179-2	MI0014216	na	na	hsa-miR-3179-3p	MIMAT0015056
440_	hsa-mir-3179-3	MI0014221	na	na	hsa-miR-3179-3p	MIMAT0015056
441_	hsa-mir-3180-1	MI0014214	hsa-miR-3180-5p	MIMAT0015057	hsa-miR-3180-3p	MIMAT0015058
442_	hsa-mir-3180-2	MI0014215	hsa-miR-3180-5p	MIMAT0015057	hsa-miR-3180-3p	MIMAT0015058
443_	hsa-mir-3180-3	MI0014217	hsa-miR-3180-5p	MIMAT0015057	hsa-miR-3180-3p	MIMAT0015058
444_	hsa-mir-3180-4	MI0016408	hsa-miR-3180-5p	MIMAT0018178	hsa-miR-3180-3p	MIMAT0015058
445_	hsa-mir-3180-5	MI0016409	hsa-miR-3180-5p	MIMAT0018178	hsa-miR-3180-3p	MIMAT0015058
446_	hsa-mir-3181	MI0014223	hsa-miR-3181-5p	MIMAT0015061	na	na
447_	hsa-mir-3182	MI0014224	hsa-miR-3182-5p	MIMAT0015062	na	na
448_	hsa-mir-3183	MI0014225	hsa-miR-3183-5p	MIMAT0015063	na	na
449_	hsa-mir-3184	MI0014226	hsa-miR-3184-5p	MIMAT0015064	hsa-miR-3184-3p	MIMAT0022731
450_	hsa-mir-3185	MI0014227	hsa-miR-3185-5p	MIMAT0015065	na	na
451_	hsa-mir-3186	MI0014229	hsa-miR-3186-5p	MIMAT0015067	hsa-miR-3186-3p	MIMAT0015068
452_	hsa-mir-3187	MI0014231	hsa-miR-3187-5p	MIMAT0019216	hsa-miR-3187-3p	MIMAT0015069
453_	hsa-mir-3188	MI0014232	na	na	hsa-miR-3188-3p	MIMAT0015070
454_	hsa-mir-3189	MI0014233	hsa-miR-3189-5p	MIMAT0019217	hsa-miR-3189-3p	MIMAT0015071
455_	hsa-mir-3190	MI0014235	hsa-miR-3190-5p	MIMAT0015073	hsa-miR-3190-3p	MIMAT0022839
456_	hsa-mir-3191	MI0014236	hsa-miR-3191-5p	MIMAT0022732	hsa-miR-3191-3p	MIMAT0015075
457_	hsa-mir-3192	MI0014237	hsa-miR-3192-5p	MIMAT0015076	hsa-miR-3192-3p	MIMAT0027027
458_	hsa-mir-3193	MI0014238	hsa-miR-3193-5p	MIMAT0015077	na	na
459_	hsa-mir-3194	MI0014239	hsa-miR-3194-5p	MIMAT0015078	hsa-miR-3194-3p	MIMAT0019218
460_	hsa-mir-3195	MI0014240	hsa-miR-3195-5p	MIMAT0015079	na	na
461_	hsa-mir-3196	MI0014241	hsa-miR-3196-5p	MIMAT0015080	na	na

462_	hsa-mir-3197	MI0014245	hsa-miR-3197-5p	MIMAT0015082	na	na
463_	hsa-mir-3198-1	MI0014246	na	na	hsa-miR-3198-3p	MIMAT0015083
464_	hsa-mir-3198-2	MI0017335	na	na	hsa-miR-3198-3p	MIMAT0015083
465_	hsa-mir-3199-1	MI0014247	hsa-miR-3199-5p	MIMAT0015084	na	na
466_	hsa-mir-3199-2	MI0014248	hsa-miR-3199-5p	MIMAT0015084	na	na
467_	hsa-mir-32	MI0000090	hsa-miR-32-5p	MIMAT0000090	hsa-miR-32-3p	MIMAT0004505
468_	hsa-mir-3200	MI0014249	hsa-miR-3200-5p	MIMAT0017392	hsa-miR-3200-3p	MIMAT0015085
469_	hsa-mir-3201	MI0014250	hsa-miR-3201-5p	MIMAT0015086	na	na
470_	hsa-mir-3202-1	MI0014252	hsa-miR-3202-5p	MIMAT0015089	na	na
471_	hsa-mir-3202-2	MI0014253	hsa-miR-3202-5p	MIMAT0015089	na	na
472_	hsa-mir-320a	MI0000542	na	na	hsa-miR-320a-3p	MIMAT0000510
473_	hsa-mir-320b-1	MI0003776	na	na	hsa-miR-320b-3p	MIMAT0005792
474_	hsa-mir-320b-2	MI0003839	na	na	hsa-miR-320b-3p	MIMAT0005792
475_	hsa-mir-320c-1	MI0003778	na	na	hsa-miR-320c-3p	MIMAT0005793
476_	hsa-mir-320c-2	MI0008191	na	na	hsa-miR-320c-3p	MIMAT0005793
477_	hsa-mir-320d-1	MI0008190	na	na	hsa-miR-320d-3p	MIMAT0006764
478_	hsa-mir-320d-2	MI0008192	na	na	hsa-miR-320d-3p	MIMAT0006764
479_	hsa-mir-320e	MI0014234	na	na	hsa-miR-320e-3p	MIMAT0015072
480_	hsa-mir-323a	MI0000807	hsa-miR-323a-5p	MIMAT0004696	hsa-miR-323a-3p	MIMAT0000755
481_	hsa-mir-323b	MI0014206	hsa-miR-323b-5p	MIMAT0001630	hsa-miR-323b-3p	MIMAT0015050
482_	hsa-mir-324	MI0000813	hsa-miR-324-5p	MIMAT0000761	hsa-miR-324-3p	MIMAT0000762
483_	hsa-mir-325	MI0000824	hsa-miR-325-5p	MIMAT0000771	na	na
484_	hsa-mir-326	MI0000808	na	na	hsa-miR-326-3p	MIMAT0000756
485_	hsa-mir-328	MI0000804	hsa-miR-328-5p	MIMAT0026486	hsa-miR-328-3p	MIMAT0000752
486_	hsa-mir-329-1	MI0001725	hsa-miR-329-5p	MIMAT0026555	hsa-miR-329-3p	MIMAT0001629
487_	hsa-mir-329-2	MI0001726	hsa-miR-329-5p	MIMAT0026555	hsa-miR-329-3p	MIMAT0001629
488_	hsa-mir-330	MI0000803	hsa-miR-330-5p	MIMAT0004693	hsa-miR-330-3p	MIMAT0000751
489_	hsa-mir-331	MI0000812	hsa-miR-331-5p	MIMAT0004700	hsa-miR-331-3p	MIMAT0000760

490_	hsa-mir-335	MI0000816	hsa-miR-335-5p	MIMAT0000765	hsa-miR-335-3p	MIMAT0004703
491_	hsa-mir-337	MI0000806	hsa-miR-337-5p	MIMAT0004695	hsa-miR-337-3p	MIMAT0000754
492_	hsa-mir-338	MI0000814	hsa-miR-338-5p	MIMAT0004701	hsa-miR-338-3p	MIMAT0000763
493_	hsa-mir-339	MI0000815	hsa-miR-339-5p	MIMAT0000764	hsa-miR-339-3p	MIMAT0004702
494_	hsa-mir-33a	MI0000091	hsa-miR-33a-5p	MIMAT0000091	hsa-miR-33a-3p	MIMAT0004506
495_	hsa-mir-33b	MI0003646	hsa-miR-33b-5p	MIMAT0003301	hsa-miR-33b-3p	MIMAT0004811
496_	hsa-mir-340	MI0000802	hsa-miR-340-5p	MIMAT0004692	hsa-miR-340-3p	MIMAT0000750
497_	hsa-mir-342	MI0000805	hsa-miR-342-5p	MIMAT0004694	hsa-miR-342-3p	MIMAT0000753
498_	hsa-mir-345	MI0000825	hsa-miR-345-5p	MIMAT0000772	hsa-miR-345-3p	MIMAT0022698
499_	hsa-mir-346	MI0000826	hsa-miR-346-5p	MIMAT0000773	na	na
500_	hsa-mir-34a	MI0000268	hsa-miR-34a-5p	MIMAT0000255	hsa-miR-34a-3p	MIMAT0004557
501_	hsa-mir-34b	MI0000742	hsa-miR-34b-5p	MIMAT0000685	hsa-miR-34b-3p	MIMAT0004676
502_	hsa-mir-34c	MI0000743	hsa-miR-34c-5p	MIMAT0000686	hsa-miR-34c-3p	MIMAT0004677
503_	hsa-mir-3529	MI0017351	hsa-miR-3529-5p	MIMAT0019828	hsa-miR-3529-3p	MIMAT0022741
504_	hsa-mir-3591	MI0017383	hsa-miR-3591-5p	MIMAT0019876	hsa-miR-3591-3p	MIMAT0019877
505_	hsa-mir-3605	MI0015995	hsa-miR-3605-5p	MIMAT0017981	hsa-miR-3605-3p	MIMAT0017982
506_	hsa-mir-3606	MI0015996	hsa-miR-3606-5p	MIMAT0017983	hsa-miR-3606-3p	MIMAT0022965
507_	hsa-mir-3607	MI0015997	hsa-miR-3607-5p	MIMAT0017984	hsa-miR-3607-3p	MIMAT0017985
508_	hsa-mir-3609	MI0015999	na	na	hsa-miR-3609-3p	MIMAT0017986
509_	hsa-mir-361	MI0000760	hsa-miR-361-5p	MIMAT0000703	hsa-miR-361-3p	MIMAT0004682
510_	hsa-mir-3610	MI0016000	na	na	hsa-miR-3610-3p	MIMAT0017987
511_	hsa-mir-3611	MI0016001	na	na	hsa-miR-3611-3p	MIMAT0017988
512_	hsa-mir-3612	MI0016002	hsa-miR-3612-5p	MIMAT0017989	na	na
513_	hsa-mir-3613	MI0016003	hsa-miR-3613-5p	MIMAT0017990	hsa-miR-3613-3p	MIMAT0017991
514_	hsa-mir-3614	MI0016004	hsa-miR-3614-5p	MIMAT0017992	hsa-miR-3614-3p	MIMAT0017993
515_	hsa-mir-3615	MI0016005	na	na	hsa-miR-3615-3p	MIMAT0017994
516_	hsa-mir-3616	MI0016006	hsa-miR-3616-5p	MIMAT0017995	hsa-miR-3616-3p	MIMAT0017996
517_	hsa-mir-3617	MI0016007	hsa-miR-3617-5p	MIMAT0017997	hsa-miR-3617-3p	MIMAT0022966

518_	hsa-mir-3618	MI0016008	na	na	hsa-miR-3618-3p	MIMAT0017998
519_	hsa-mir-3619	MI0016009	hsa-miR-3619-5p	MIMAT0017999	hsa-miR-3619-3p	MIMAT0019219
520_	hsa-mir-362	MI0000762	hsa-miR-362-5p	MIMAT0000705	hsa-miR-362-3p	MIMAT0004683
521_	hsa-mir-3620	MI0016011	hsa-miR-3620-5p	MIMAT0022967	hsa-miR-3620-3p	MIMAT0018001
522_	hsa-mir-3621	MI0016012	hsa-miR-3621-5p	MIMAT0018002	na	na
523_	hsa-mir-3622a	MI0016013	hsa-miR-3622a-5p	MIMAT0018003	hsa-miR-3622a-3p	MIMAT0018004
524_	hsa-mir-3622b	MI0016014	hsa-miR-3622b-5p	MIMAT0018005	hsa-miR-3622b-3p	MIMAT0018006
525_	hsa-mir-363	MI0000764	hsa-miR-363-5p	MIMAT0003385	hsa-miR-363-3p	MIMAT0000707
526_	hsa-mir-3646	MI0016046	na	na	hsa-miR-3646-3p	MIMAT0018065
527_	hsa-mir-3648	MI0016048	hsa-miR-3648-5p	MIMAT0018068	na	na
528_	hsa-mir-3649	MI0016049	na	na	hsa-miR-3649-3p	MIMAT0018069
529_	hsa-mir-3650	MI0016050	hsa-miR-3650-5p	MIMAT0018070	na	na
530_	hsa-mir-3651	MI0016051	na	na	hsa-miR-3651-3p	MIMAT0018071
531_	hsa-mir-3652	MI0016052	hsa-miR-3652-5p	MIMAT0018072	na	na
532_	hsa-mir-3653	MI0016053	na	na	hsa-miR-3653-3p	MIMAT0018073
533_	hsa-mir-3654	MI0016054	na	na	hsa-miR-3654-3p	MIMAT0018074
534_	hsa-mir-3655	MI0016055	hsa-miR-3655-5p	MIMAT0018075	na	na
535_	hsa-mir-3656	MI0016056	na	na	hsa-miR-3656-3p	MIMAT0018076
536_	hsa-mir-3657	MI0016057	na	na	hsa-miR-3657-3p	MIMAT0018077
537_	hsa-mir-3658	MI0016058	hsa-miR-3658-5p	MIMAT0018078	na	na
538_	hsa-mir-3659	MI0016060	na	na	hsa-miR-3659-3p	MIMAT0018080
539_	hsa-mir-365a	MI0000767	hsa-miR-365a-5p	MIMAT0009199	hsa-miR-365a-3p	MIMAT0000710
540_	hsa-mir-365b	MI0000769	hsa-miR-365b-5p	MIMAT0022833	hsa-miR-365b-3p	MIMAT0022834
541_	hsa-mir-3660	MI0016061	na	na	hsa-miR-3660-3p	MIMAT0018081
542_	hsa-mir-3661	MI0016062	hsa-miR-3661-5p	MIMAT0018082	na	na
543_	hsa-mir-3662	MI0016063	na	na	hsa-miR-3662-3p	MIMAT0018083
544_	hsa-mir-3663	MI0016064	hsa-miR-3663-5p	MIMAT0018084	hsa-miR-3663-3p	MIMAT0018085
545_	hsa-mir-3664	MI0016065	hsa-miR-3664-5p	MIMAT0018086	hsa-miR-3664-3p	MIMAT0019220

546_	hsa-mir-3665	MI0016066	hsa-miR-3665-5p	MIMAT0018087	na	na
547_	hsa-mir-3666	MI0016067	hsa-miR-3666-5p	MIMAT0018088	na	na
548_	hsa-mir-3667	MI0016068	hsa-miR-3667-5p	MIMAT0018089	hsa-miR-3667-3p	MIMAT0018090
549_	hsa-mir-3668	MI0016069	hsa-miR-3668-5p	MIMAT0018091	na	na
550_	hsa-mir-3669	MI0016070	na	na	hsa-miR-3669-3p	MIMAT0018092
551_	hsa-mir-367	MI0000775	hsa-miR-367-5p	MIMAT0004686	hsa-miR-367-3p	MIMAT0000719
552_	hsa-mir-3670-1	MI0016071	na	na	hsa-miR-3670-3p	MIMAT0018093
553_	hsa-mir-3670-2	MI0019112	na	na	hsa-miR-3670-3p	MIMAT0018093
554_	hsa-mir-3671	MI0016072	na	na	hsa-miR-3671-3p	MIMAT0018094
555_	hsa-mir-3672	MI0016073	hsa-miR-3672-5p	MIMAT0018095	na	na
556_	hsa-mir-3673	MI0016074	hsa-miR-3673-5p	MIMAT0018096	na	na
557_	hsa-mir-3674	MI0016075	hsa-miR-3674-5p	MIMAT0018097	na	na
558_	hsa-mir-3675	MI0016076	hsa-miR-3675-5p	MIMAT0018098	hsa-miR-3675-3p	MIMAT0018099
559_	hsa-mir-3677	MI0016078	hsa-miR-3677-5p	MIMAT0019221	hsa-miR-3677-3p	MIMAT0018101
560_	hsa-mir-3678	MI0016079	hsa-miR-3678-5p	MIMAT0018102	hsa-miR-3678-3p	MIMAT0018103
561_	hsa-mir-3679	MI0016080	hsa-miR-3679-5p	MIMAT0018104	hsa-miR-3679-3p	MIMAT0018105
562_	hsa-mir-3680-1	MI0016081	hsa-miR-3680-5p	MIMAT0018106	hsa-miR-3680-3p	MIMAT0018107
563_	hsa-mir-3680-2	MI0019113	hsa-miR-3680-5p	MIMAT0018106	hsa-miR-3680-3p	MIMAT0018107
564_	hsa-mir-3681	MI0016082	hsa-miR-3681-5p	MIMAT0018108	hsa-miR-3681-3p	MIMAT0018109
565_	hsa-mir-3682	MI0016083	hsa-miR-3682-5p	MIMAT0019222	hsa-miR-3682-3p	MIMAT0018110
566_	hsa-mir-3683	MI0016084	hsa-miR-3683-5p	MIMAT0018111	na	na
567_	hsa-mir-3684	MI0016085	na	na	hsa-miR-3684-3p	MIMAT0018112
568_	hsa-mir-3685	MI0016086	hsa-miR-3685-5p	MIMAT0018113	na	na
569_	hsa-mir-3686	MI0016087	na	na	hsa-miR-3686-3p	MIMAT0018114
570_	hsa-mir-3687	MI0016088	na	na	hsa-miR-3687-3p	MIMAT0018115
571_	hsa-mir-3688-1	MI0016089	hsa-miR-3688-5p	MIMAT0019223	hsa-miR-3688-3p	MIMAT0018116
572_	hsa-mir-3688-2	MI0017447	hsa-miR-3688-5p	MIMAT0019223	hsa-miR-3688-3p	MIMAT0018116
573_	hsa-mir-3689a	MI0016090	hsa-miR-3689a-5p	MIMAT0018117	hsa-miR-3689a-3p	MIMAT0018118

574_	hsa-mir-3689b	MI0016411	hsa-miR-3689b-5p	MIMAT0018180	hsa-miR-3689b-3p	MIMAT0018181
575_	hsa-mir-3689c	MI0016832	na	na	hsa-miR-3689c-3p	MIMAT0019007
576_	hsa-mir-3689d-1	MI0016834	hsa-miR-3689d-5p	MIMAT0019008	na	na
577_	hsa-mir-3689d-2	MI0016835	hsa-miR-3689d-5p	MIMAT0019008	na	na
578_	hsa-mir-3689e	MI0016836	hsa-miR-3689e-5p	MIMAT0019009	na	na
579_	hsa-mir-3689f	MI0016837	hsa-miR-3689f-5p	MIMAT0019010	na	na
580_	hsa-mir-369	MI0000777	hsa-miR-369-5p	MIMAT0001621	hsa-miR-369-3p	MIMAT0000721
581_	hsa-mir-3690-1	MI0016091	hsa-miR-3690-5p	MIMAT0018119	na	na
582_	hsa-mir-3690-2	MI0023561	hsa-miR-3690-5p	MIMAT0018119	na	na
583_	hsa-mir-3691	MI0016092	hsa-miR-3691-5p	MIMAT0018120	hsa-miR-3691-3p	MIMAT0019224
584_	hsa-mir-3692	MI0016093	hsa-miR-3692-5p	MIMAT0018121	hsa-miR-3692-3p	MIMAT0018122
585_	hsa-mir-370	MI0000778	hsa-miR-370-5p	MIMAT0026483	hsa-miR-370-3p	MIMAT0000722
586_	hsa-mir-3713	MI0016134	hsa-miR-3713-5p	MIMAT0018164	na	na
587_	hsa-mir-3714	MI0016135	hsa-miR-3714-5p	MIMAT0018165	na	na
588_	hsa-mir-371a	MI0000779	hsa-miR-371a-5p	MIMAT0004687	hsa-miR-371a-3p	MIMAT0000723
589_	hsa-mir-371b	MI0017393	hsa-miR-371b-5p	MIMAT0019892	hsa-miR-371b-3p	MIMAT0019893
590_	hsa-mir-372	MI0000780	hsa-miR-372-5p	MIMAT0026484	hsa-miR-372-3p	MIMAT0000724
591_	hsa-mir-373	MI0000781	hsa-miR-373-5p	MIMAT0000725	hsa-miR-373-3p	MIMAT0000726
592_	hsa-mir-374a	MI0000782	hsa-miR-374a-5p	MIMAT0000727	hsa-miR-374a-3p	MIMAT0004688
593_	hsa-mir-374b	MI0005566	hsa-miR-374b-5p	MIMAT0004955	hsa-miR-374b-3p	MIMAT0004956
594_	hsa-mir-374c	MI0016684	hsa-miR-374c-5p	MIMAT0018443	hsa-miR-374c-3p	MIMAT0022735
595_	hsa-mir-375	MI0000783	na	na	hsa-miR-375-3p	MIMAT0000728
596_	hsa-mir-376a-1	MI0000784	hsa-miR-376a-5p	MIMAT0003386	hsa-miR-376a-3p	MIMAT0000729
597_	hsa-mir-376a-2	MI0003529	hsa-miR-376a-2-5p	MIMAT0022928	hsa-miR-376a-3p	MIMAT0000729
598_	hsa-mir-376b	MI0002466	hsa-miR-376b-5p	MIMAT0022923	hsa-miR-376b-3p	MIMAT0002172
599_	hsa-mir-376c	MI0000776	hsa-miR-376c-5p	MIMAT0022861	hsa-miR-376c-3p	MIMAT0000720
600_	hsa-mir-377	MI0000785	hsa-miR-377-5p	MIMAT0004689	hsa-miR-377-3p	MIMAT0000730
601_	hsa-mir-378a	MI0000786	hsa-miR-378a-5p	MIMAT0000731	hsa-miR-378a-3p	MIMAT0000732

602_	hsa-mir-378b	MI0014154	na	na	hsa-miR-378b-3p	MIMAT0014999
603_	hsa-mir-378c	MI0015825	hsa-miR-378c-5p	MIMAT0016847	na	na
604_	hsa-mir-378d-1	MI0016749	hsa-miR-378d-5p	MIMAT0018926	na	na
605_	hsa-mir-378d-2	MI0003840	hsa-miR-378d-5p	MIMAT0018926	na	na
606_	hsa-mir-378e	MI0016750	na	na	hsa-miR-378e-3p	MIMAT0018927
607_	hsa-mir-378f	MI0016756	na	na	hsa-miR-378f-3p	MIMAT0018932
608_	hsa-mir-378g	MI0016761	hsa-miR-378g-5p	MIMAT0018937	na	na
609_	hsa-mir-378h	MI0016808	hsa-miR-378h-5p	MIMAT0018984	na	na
610_	hsa-mir-378i	MI0016902	hsa-miR-378i-5p	MIMAT0019074	na	na
611_	hsa-mir-378j	MI0021273	hsa-miR-378j-5p	MIMAT0024612	na	na
612_	hsa-mir-379	MI0000787	hsa-miR-379-5p	MIMAT0000733	hsa-miR-379-3p	MIMAT0004690
613_	hsa-mir-380	MI0000788	hsa-miR-380-5p	MIMAT0000734	hsa-miR-380-3p	MIMAT0000735
614_	hsa-mir-381	MI0000789	hsa-miR-381-5p	MIMAT0022862	hsa-miR-381-3p	MIMAT0000736
615_	hsa-mir-382	MI0000790	hsa-miR-382-5p	MIMAT0000737	hsa-miR-382-3p	MIMAT0022697
616_	hsa-mir-383	MI0000791	hsa-miR-383-5p	MIMAT0000738	hsa-miR-383-3p	MIMAT0026485
617_	hsa-mir-384	MI0001145	na	na	hsa-miR-384-3p	MIMAT0001075
618_	hsa-mir-3907	MI0016410	na	na	hsa-miR-3907-3p	MIMAT0018179
619_	hsa-mir-3908	MI0016412	hsa-miR-3908-5p	MIMAT0018182	na	na
620_	hsa-mir-3909	MI0016413	na	na	hsa-miR-3909-3p	MIMAT0018183
621_	hsa-mir-3910-1	MI0016414	na	na	hsa-miR-3910-3p	MIMAT0018184
622_	hsa-mir-3910-2	MI0016431	na	na	hsa-miR-3910-3p	MIMAT0018184
623_	hsa-mir-3911	MI0016415	hsa-miR-3911-5p	MIMAT0018185	na	na
624_	hsa-mir-3912	MI0016416	hsa-miR-3912-5p	MIMAT0027036	hsa-miR-3912-3p	MIMAT0018186
625_	hsa-mir-3913-1	MI0016417	hsa-miR-3913-5p	MIMAT0018187	hsa-miR-3913-3p	MIMAT0019225
626_	hsa-mir-3913-2	MI0016418	hsa-miR-3913-5p	MIMAT0018187	hsa-miR-3913-3p	MIMAT0019225
627_	hsa-mir-3914-1	MI0016419	na	na	hsa-miR-3914-3p	MIMAT0018188
628_	hsa-mir-3914-2	MI0016421	na	na	hsa-miR-3914-3p	MIMAT0018188

629_	hsa-mir-3915	MI0016420	hsa-miR-3915-5p	MIMAT0018189	na	na
630_	hsa-mir-3916	MI0016422	hsa-miR-3916-5p	MIMAT0018190	na	na
631_	hsa-mir-3917	MI0016423	na	na	hsa-miR-3917-3p	MIMAT0018191
632_	hsa-mir-3918	MI0016424	hsa-miR-3918-5p	MIMAT0018192	na	na
633_	hsa-mir-3919	MI0016425	na	na	hsa-miR-3919-3p	MIMAT0018193
634_	hsa-mir-3920	MI0016427	na	na	hsa-miR-3920-3p	MIMAT0018195
635_	hsa-mir-3921	MI0016428	na	na	hsa-miR-3921-3p	MIMAT0018196
636_	hsa-mir-3922	MI0016429	hsa-miR-3922-5p	MIMAT0019227	hsa-miR-3922-3p	MIMAT0018197
637_	hsa-mir-3923	MI0016430	na	na	hsa-miR-3923-3p	MIMAT0018198
638_	hsa-mir-3924	MI0016432	na	na	hsa-miR-3924-3p	MIMAT0018199
639_	hsa-mir-3925	MI0016433	hsa-miR-3925-5p	MIMAT0018200	hsa-miR-3925-3p	MIMAT0019228
640_	hsa-mir-3926-1	MI0016434	hsa-miR-3926-5p	MIMAT0018201	na	na
641_	hsa-mir-3926-2	MI0016437	hsa-miR-3926-5p	MIMAT0018201	na	na
642_	hsa-mir-3927	MI0016435	hsa-miR-3927-5p	MIMAT0022970	hsa-miR-3927-3p	MIMAT0018202
643_	hsa-mir-3928	MI0016438	hsa-miR-3928-5p	MIMAT0027037	hsa-miR-3928-3p	MIMAT0018205
644_	hsa-mir-3929	MI0016439	na	na	hsa-miR-3929-3p	MIMAT0018206
645_	hsa-mir-3934	MI0016590	hsa-miR-3934-5p	MIMAT0018349	hsa-miR-3934-3p	MIMAT0022975
646_	hsa-mir-3935	MI0016591	na	na	hsa-miR-3935-3p	MIMAT0018350
647_	hsa-mir-3936	MI0016592	na	na	hsa-miR-3936-3p	MIMAT0018351
648_	hsa-mir-3937	MI0016593	na	na	hsa-miR-3937-3p	MIMAT0018352
649_	hsa-mir-3938	MI0016594	na	na	hsa-miR-3938-3p	MIMAT0018353
650_	hsa-mir-3939	MI0016596	na	na	hsa-miR-3939-3p	MIMAT0018355
651_	hsa-mir-3940	MI0016597	hsa-miR-3940-5p	MIMAT0019229	hsa-miR-3940-3p	MIMAT0018356
652_	hsa-mir-3941	MI0016598	na	na	hsa-miR-3941-3p	MIMAT0018357
653_	hsa-mir-3942	MI0016599	hsa-miR-3942-5p	MIMAT0018358	hsa-miR-3942-3p	MIMAT0019230
654_	hsa-mir-3943	MI0016600	hsa-miR-3943-5p	MIMAT0018359	na	na
655_	hsa-mir-3944	MI0016601	hsa-miR-3944-5p	MIMAT0019231	hsa-miR-3944-3p	MIMAT0018360

656_	hsa-mir-3945	MI0016602	hsa-miR-3945-5p	MIMAT0018361	na	na
657_	hsa-mir-3960	MI0016964	na	na	hsa-miR-3960-3p	MIMAT0019337
658_	hsa-mir-3972	MI0016990	na	na	hsa-miR-3972-3p	MIMAT0019357
659_	hsa-mir-3973	MI0016991	na	na	hsa-miR-3973-3p	MIMAT0019358
660_	hsa-mir-3974	MI0016992	hsa-miR-3974-5p	MIMAT0019359	na	na
661_	hsa-mir-3975	MI0016993	na	na	hsa-miR-3975-3p	MIMAT0019360
662_	hsa-mir-3976	MI0016994	hsa-miR-3976-5p	MIMAT0019361	na	na
663_	hsa-mir-3977	MI0016995	hsa-miR-3977-5p	MIMAT0019362	na	na
664_	hsa-mir-3978	MI0016996	hsa-miR-3978-5p	MIMAT0019363	na	na
665_	hsa-mir-409	MI0001735	hsa-miR-409-5p	MIMAT0001638	hsa-miR-409-3p	MIMAT0001639
666_	hsa-mir-410	MI0002465	hsa-miR-410-5p	MIMAT0026558	hsa-miR-410-3p	MIMAT0002171
667_	hsa-mir-411	MI0003675	hsa-miR-411-5p	MIMAT0003329	hsa-miR-411-3p	MIMAT0004813
668_	hsa-mir-412	MI0002464	hsa-miR-412-5p	MIMAT0026557	hsa-miR-412-3p	MIMAT0002170
669_	hsa-mir-421	MI0003685	na	na	hsa-miR-421-3p	MIMAT0003339
670_	hsa-mir-422a	MI0001444	hsa-miR-422a-5p	MIMAT0001339	na	na
671_	hsa-mir-423	MI0001445	hsa-miR-423-5p	MIMAT0004748	hsa-miR-423-3p	MIMAT0001340
672_	hsa-mir-424	MI0001446	hsa-miR-424-5p	MIMAT0001341	hsa-miR-424-3p	MIMAT0004749
673_	hsa-mir-425	MI0001448	hsa-miR-425-5p	MIMAT0003393	hsa-miR-425-3p	MIMAT0001343
674_	hsa-mir-4251	MI0015861	na	na	hsa-miR-4251-3p	MIMAT0016883
675_	hsa-mir-4252	MI0015864	na	na	hsa-miR-4252-3p	MIMAT0016886
676_	hsa-mir-4253	MI0015860	na	na	hsa-miR-4253-3p	MIMAT0016882
677_	hsa-mir-4254	MI0015862	na	na	hsa-miR-4254-3p	MIMAT0016884
678_	hsa-mir-4255	MI0015863	hsa-miR-4255-5p	MIMAT0016885	na	na
679_	hsa-mir-4256	MI0015855	hsa-miR-4256-5p	MIMAT0016877	na	na
680_	hsa-mir-4257	MI0015856	na	na	hsa-miR-4257-3p	MIMAT0016878
681_	hsa-mir-4258	MI0015857	hsa-miR-4258-5p	MIMAT0016879	na	na
682_	hsa-mir-4259	MI0015858	na	na	hsa-miR-4259-3p	MIMAT0016880
683_	hsa-mir-4260	MI0015859	hsa-miR-4260-5p	MIMAT0016881	na	na

684_	hsa-mir-4261	MI0015868	na	na	hsa-miR-4261-3p	MIMAT0016890
685_	hsa-mir-4262	MI0015872	na	na	na	na
686_	hsa-mir-4263	MI0015876	na	na	hsa-miR-4263-3p	MIMAT0016898
687_	hsa-mir-4264	MI0015877	hsa-miR-4264-5p	MIMAT0016899	na	na
688_	hsa-mir-4265	MI0015869	na	na	hsa-miR-4265-3p	MIMAT0016891
689_	hsa-mir-4266	MI0015870	na	na	hsa-miR-4266-3p	MIMAT0016892
690_	hsa-mir-4267	MI0015871	hsa-miR-4267-5p	MIMAT0016893	na	na
691_	hsa-mir-4268	MI0015874	na	na	hsa-miR-4268-3p	MIMAT0016896
692_	hsa-mir-4269	MI0015875	hsa-miR-4269-5p	MIMAT0016897	na	na
693_	hsa-mir-4270	MI0015878	hsa-miR-4270-5p	MIMAT0016900	na	na
694_	hsa-mir-4271	MI0015879	na	na	hsa-miR-4271-3p	MIMAT0016901
695_	hsa-mir-4272	MI0015880	na	na	hsa-miR-4272-3p	MIMAT0016902
696_	hsa-mir-4273	MI0015881	hsa-miR-4273-5p	MIMAT0016903	na	na
697_	hsa-mir-4274	MI0015884	na	na	hsa-miR-4274-3p	MIMAT0016906
698_	hsa-mir-4275	MI0015883	hsa-miR-4275-5p	MIMAT0016905	na	na
699_	hsa-mir-4276	MI0015882	hsa-miR-4276-5p	MIMAT0016904	na	na
700_	hsa-mir-4277	MI0015886	hsa-miR-4277-5p	MIMAT0016908	na	na
701_	hsa-mir-4278	MI0015888	na	na	hsa-miR-4278-3p	MIMAT0016910
702_	hsa-mir-4279	MI0015887	na	na	hsa-miR-4279-3p	MIMAT0016909
703_	hsa-mir-4280	MI0015889	hsa-miR-4280-5p	MIMAT0016911	na	na
704_	hsa-mir-4281	MI0015885	na	na	hsa-miR-4281-3p	MIMAT0016907
705_	hsa-mir-4282	MI0015890	na	na	hsa-miR-4282-3p	MIMAT0016912
706_	hsa-mir-4283-1	MI0015892	hsa-miR-4283-5p	MIMAT0016914	na	na
707_	hsa-mir-4283-2	MI0015982	hsa-miR-4283-5p	MIMAT0016914	na	na
708_	hsa-mir-4284	MI0015893	hsa-miR-4284-5p	MIMAT0016915	na	na
709_	hsa-mir-4285	MI0015891	hsa-miR-4285-5p	MIMAT0016913	na	na
710_	hsa-mir-4286	MI0015894	hsa-miR-4286-5p	MIMAT0016916	na	na
711_	hsa-mir-4287	MI0015895	hsa-miR-4287-5p	MIMAT0016917	na	na

712_	hsa-mir-4288	MI0015896	na	na	hsa-miR-4288-3p	MIMAT0016918
713_	hsa-mir-4289	MI0015898	hsa-miR-4289-5p	MIMAT0016920	na	na
714_	hsa-mir-429	MI0001641	na	na	hsa-miR-429-3p	MIMAT0001536
715_	hsa-mir-4290	MI0015899	na	na	hsa-miR-4290-3p	MIMAT0016921
716_	hsa-mir-4291	MI0015900	hsa-miR-4291-5p	MIMAT0016922	na	na
717_	hsa-mir-4292	MI0015897	na	na	hsa-miR-4292-3p	MIMAT0016919
718_	hsa-mir-4293	MI0015826	na	na	hsa-miR-4293-3p	MIMAT0016848
719_	hsa-mir-4294	MI0015827	hsa-miR-4294-5p	MIMAT0016849	na	na
720_	hsa-mir-4295	MI0015822	hsa-miR-4295-5p	MIMAT0016844	na	na
721_	hsa-mir-4296	MI0015823	hsa-miR-4296-5p	MIMAT0016845	na	na
722_	hsa-mir-4297	MI0015824	hsa-miR-4297-5p	MIMAT0016846	na	na
723_	hsa-mir-4298	MI0015830	hsa-miR-4298-5p	MIMAT0016852	na	na
724_	hsa-mir-4299	MI0015829	na	na	hsa-miR-4299-3p	MIMAT0016851
725_	hsa-mir-4300	MI0015831	na	na	hsa-miR-4300-3p	MIMAT0016853
726_	hsa-mir-4301	MI0015828	hsa-miR-4301-5p	MIMAT0016850	na	na
727_	hsa-mir-4302	MI0015833	hsa-miR-4302-5p	MIMAT0016855	na	na
728_	hsa-mir-4303	MI0015834	hsa-miR-4303-5p	MIMAT0016856	na	na
729_	hsa-mir-4304	MI0015832	hsa-miR-4304-5p	MIMAT0016854	na	na
730_	hsa-mir-4305	MI0015835	hsa-miR-4305-5p	MIMAT0016857	na	na
731_	hsa-mir-4306	MI0015836	na	na	hsa-miR-4306-3p	MIMAT0016858
732_	hsa-mir-4307	MI0015838	na	na	hsa-miR-4307-3p	MIMAT0016860
733_	hsa-mir-4308	MI0015839	na	na	hsa-miR-4308-3p	MIMAT0016861
734_	hsa-mir-4309	MI0015837	hsa-miR-4309-5p	MIMAT0016859	na	na
735_	hsa-mir-431	MI0001721	hsa-miR-431-5p	MIMAT0001625	hsa-miR-431-3p	MIMAT0004757
736_	hsa-mir-4310	MI0015840	na	na	hsa-miR-4310-3p	MIMAT0016862
737_	hsa-mir-4311	MI0015841	hsa-miR-4311-5p	MIMAT0016863	na	na
738_	hsa-mir-4312	MI0015842	na	na	hsa-miR-4312-3p	MIMAT0016864
739_	hsa-mir-4313	MI0015843	hsa-miR-4313-5p	MIMAT0016865	na	na

740_	hsa-mir-4314	MI0015846	hsa-miR-4314-5p	MIMAT0016868	na	na
741_	hsa-mir-4315-1	MI0015844	hsa-miR-4315-5p	MIMAT0016866	na	na
742_	hsa-mir-4315-2	MI0015983	hsa-miR-4315-5p	MIMAT0016866	na	na
743_	hsa-mir-4316	MI0015845	hsa-miR-4316-5p	MIMAT0016867	na	na
744_	hsa-mir-4317	MI0015850	hsa-miR-4317-5p	MIMAT0016872	na	na
745_	hsa-mir-4318	MI0015847	na	na	hsa-miR-4318-3p	MIMAT0016869
746_	hsa-mir-4319	MI0015848	hsa-miR-4319-5p	MIMAT0016870	na	na
747_	hsa-mir-432	MI0003133	hsa-miR-432-5p	MIMAT0002814	hsa-miR-432-3p	MIMAT0002815
748_	hsa-mir-4320	MI0015849	na	na	hsa-miR-4320-3p	MIMAT0016871
749_	hsa-mir-4321	MI0015852	na	na	hsa-miR-4321-3p	MIMAT0016874
750_	hsa-mir-4322	MI0015851	na	na	hsa-miR-4322-3p	MIMAT0016873
751_	hsa-mir-4323	MI0015853	na	na	hsa-miR-4323-3p	MIMAT0016875
752_	hsa-mir-4324	MI0015854	na	na	hsa-miR-4324-3p	MIMAT0016876
753_	hsa-mir-4325	MI0015865	hsa-miR-4325-5p	MIMAT0016887	na	na
754_	hsa-mir-4326	MI0015866	hsa-miR-4326-5p	MIMAT0016888	na	na
755_	hsa-mir-4327	MI0015867	hsa-miR-4327-5p	MIMAT0016889	na	na
756_	hsa-mir-4328	MI0015904	na	na	hsa-miR-4328-3p	MIMAT0016926
757_	hsa-mir-4329	MI0015901	na	na	hsa-miR-4329-3p	MIMAT0016923
758_	hsa-mir-433	MI0001723	hsa-miR-433-5p	MIMAT0026554	hsa-miR-433-3p	MIMAT0001627
759_	hsa-mir-4330	MI0015902	na	na	hsa-miR-4330-3p	MIMAT0016924
760_	hsa-mir-4417	MI0016753	hsa-miR-4417-5p	MIMAT0018929	na	na
761_	hsa-mir-4418	MI0016754	na	na	hsa-miR-4418-3p	MIMAT0018930
762_	hsa-mir-4419a	MI0016755	na	na	na	na
763_	hsa-mir-4419b	MI0016861	na	na	hsa-miR-4419b-3p	MIMAT0019034
764_	hsa-mir-4420	MI0016757	na	na	hsa-miR-4420-3p	MIMAT0018933
765_	hsa-mir-4421	MI0016758	na	na	hsa-miR-4421-3p	MIMAT0018934
766_	hsa-mir-4422	MI0016759	hsa-miR-4422-5p	MIMAT0018935	na	na
767_	hsa-mir-4423	MI0016760	hsa-miR-4423-5p	MIMAT0019232	hsa-miR-4423-3p	MIMAT0018936

768_	hsa-mir-4424	MI0016763	hsa-miR-4424-5p	MIMAT0018939	na	na
769_	hsa-mir-4425	MI0016764	na	na	hsa-miR-4425-3p	MIMAT0018940
770_	hsa-mir-4426	MI0016765	hsa-miR-4426-5p	MIMAT0018941	na	na
771_	hsa-mir-4427	MI0016766	na	na	hsa-miR-4427-3p	MIMAT0018942
772_	hsa-mir-4428	MI0016767	na	na	hsa-miR-4428-3p	MIMAT0018943
773_	hsa-mir-4429	MI0016768	hsa-miR-4429-5p	MIMAT0018944	na	na
774_	hsa-mir-4430	MI0016769	hsa-miR-4430-5p	MIMAT0018945	na	na
775_	hsa-mir-4431	MI0016771	hsa-miR-4431-5p	MIMAT0018947	na	na
776_	hsa-mir-4432	MI0016772	na	na	hsa-miR-4432-3p	MIMAT0018948
777_	hsa-mir-4433	MI0016773	hsa-miR-4433-5p	MIMAT0020956	hsa-miR-4433-3p	MIMAT0018949
778_	hsa-mir-4433b	MI0025511	hsa-miR-4433b-5p	MIMAT0030413	hsa-miR-4433b-3p	MIMAT0030414
779_	hsa-mir-4434	MI0016774	hsa-miR-4434-5p	MIMAT0018950	na	na
780_	hsa-mir-4435-1	MI0016775	hsa-miR-4435-5p	MIMAT0018951	na	na
781_	hsa-mir-4435-2	MI0016777	hsa-miR-4435-5p	MIMAT0018951	na	na
782_	hsa-mir-4436a	MI0016776	na	na	hsa-miR-4436a-3p	MIMAT0018952
783_	hsa-mir-4436b-1	MI0017425	hsa-miR-4436b-5p	MIMAT0019940	hsa-miR-4436b-3p	MIMAT0019941
784_	hsa-mir-4436b-2	MI0019110	hsa-miR-4436b-5p	MIMAT0019940	hsa-miR-4436b-3p	MIMAT0019941
785_	hsa-mir-4437	MI0016778	na	na	hsa-miR-4437-3p	MIMAT0018953
786_	hsa-mir-4438	MI0016781	na	na	hsa-miR-4438-3p	MIMAT0018956
787_	hsa-mir-4439	MI0016782	hsa-miR-4439-5p	MIMAT0018957	na	na
788_	hsa-mir-4440	MI0016783	na	na	hsa-miR-4440-3p	MIMAT0018958
789_	hsa-mir-4441	MI0016784	na	na	hsa-miR-4441-3p	MIMAT0018959
790_	hsa-mir-4442	MI0016785	na	na	hsa-miR-4442-3p	MIMAT0018960
791_	hsa-mir-4443	MI0016786	hsa-miR-4443-5p	MIMAT0018961	na	na
792_	hsa-mir-4444-1	MI0016787	na	na	hsa-miR-4444-3p	MIMAT0018962
793_	hsa-mir-4444-2	MI0019111	na	na	hsa-miR-4444-3p	MIMAT0018962
794_	hsa-mir-4445	MI0016788	hsa-miR-4445-5p	MIMAT0018963	hsa-miR-4445-3p	MIMAT0018964
795_	hsa-mir-4446	MI0016789	hsa-miR-4446-5p	MIMAT0019233	hsa-miR-4446-3p	MIMAT0018965

796_	hsa-mir-4447	MI0016790	na	na	hsa-miR-4447-3p	MIMAT0018966
797_	hsa-mir-4448	MI0016791	na	na	hsa-miR-4448-3p	MIMAT0018967
798_	hsa-mir-4449	MI0016792	na	na	hsa-miR-4449-3p	MIMAT0018968
799_	hsa-mir-4450	MI0016795	hsa-miR-4450-5p	MIMAT0018971	na	na
800_	hsa-mir-4451	MI0016797	na	na	hsa-miR-4451-3p	MIMAT0018973
801_	hsa-mir-4452	MI0016798	na	na	hsa-miR-4452-3p	MIMAT0018974
802_	hsa-mir-4453	MI0016799	hsa-miR-4453-5p	MIMAT0018975	na	na
803_	hsa-mir-4454	MI0016800	hsa-miR-4454-5p	MIMAT0018976	na	na
804_	hsa-mir-4455	MI0016801	hsa-miR-4455-5p	MIMAT0018977	na	na
805_	hsa-mir-4456	MI0016802	hsa-miR-4456-5p	MIMAT0018978	na	na
806_	hsa-mir-4457	MI0016803	na	na	hsa-miR-4457-3p	MIMAT0018979
807_	hsa-mir-4458	MI0016804	hsa-miR-4458-5p	MIMAT0018980	na	na
808_	hsa-mir-4459	MI0016805	hsa-miR-4459-5p	MIMAT0018981	na	na
809_	hsa-mir-4460	MI0016806	hsa-miR-4460-5p	MIMAT0018982	na	na
810_	hsa-mir-4461	MI0016807	na	na	hsa-miR-4461-3p	MIMAT0018983
811_	hsa-mir-4462	MI0016810	na	na	hsa-miR-4462-3p	MIMAT0018986
812_	hsa-mir-4463	MI0016811	na	na	hsa-miR-4463-3p	MIMAT0018987
813_	hsa-mir-4464	MI0016812	hsa-miR-4464-5p	MIMAT0018988	na	na
814_	hsa-mir-4465	MI0016816	na	na	hsa-miR-4465-3p	MIMAT0018992
815_	hsa-mir-4466	MI0016817	hsa-miR-4466-5p	MIMAT0018993	na	na
816_	hsa-mir-4467	MI0016818	hsa-miR-4467-5p	MIMAT0018994	na	na
817_	hsa-mir-4468	MI0016819	na	na	hsa-miR-4468-3p	MIMAT0018995
818_	hsa-mir-4469	MI0016820	na	na	hsa-miR-4469-3p	MIMAT0018996
819_	hsa-mir-4470	MI0016821	na	na	hsa-miR-4470-3p	MIMAT0018997
820_	hsa-mir-4471	MI0016822	na	na	hsa-miR-4471-3p	MIMAT0018998
821_	hsa-mir-4472-1	MI0016823	hsa-miR-4472-5p	MIMAT0018999	na	na
822_	hsa-mir-4472-2	MI0016824	hsa-miR-4472-5p	MIMAT0018999	na	na
823_	hsa-mir-4473	MI0016825	na	na	hsa-miR-4473-3p	MIMAT0019000

824_	hsa-mir-4474	MI0016826	hsa-miR-4474-5p	MIMAT0019234	hsa-miR-4474-3p	MIMAT0019001
825_	hsa-mir-4475	MI0016827	na	na	hsa-miR-4475-3p	MIMAT0019002
826_	hsa-mir-4476	MI0016828	na	na	hsa-miR-4476-3p	MIMAT0019003
827_	hsa-mir-4477a	MI0016829	na	na	hsa-miR-4477a-3p	MIMAT0019004
828_	hsa-mir-4477b	MI0016830	na	na	hsa-miR-4477b-3p	MIMAT0019005
829_	hsa-mir-4478	MI0016831	hsa-miR-4478-5p	MIMAT0019006	na	na
830_	hsa-mir-4479	MI0016838	na	na	hsa-miR-4479-3p	MIMAT0019011
831_	hsa-mir-448	MI0001637	na	na	hsa-miR-448-3p	MIMAT0001532
832_	hsa-mir-4480	MI0016841	na	na	hsa-miR-4480-3p	MIMAT0019014
833_	hsa-mir-4481	MI0016842	hsa-miR-4481-5p	MIMAT0019015	na	na
834_	hsa-mir-4482	MI0016843	hsa-miR-4482-5p	MIMAT0019016	hsa-miR-4482-3p	MIMAT0020958
835_	hsa-mir-4483	MI0016844	na	na	hsa-miR-4483-3p	MIMAT0019017
836_	hsa-mir-4484	MI0016845	na	na	hsa-miR-4484-3p	MIMAT0019018
837_	hsa-mir-4485	MI0016846	na	na	hsa-miR-4485-3p	MIMAT0019019
838_	hsa-mir-4486	MI0016847	hsa-miR-4486-5p	MIMAT0019020	na	na
839_	hsa-mir-4487	MI0016848	hsa-miR-4487-5p	MIMAT0019021	na	na
840_	hsa-mir-4488	MI0016849	hsa-miR-4488-5p	MIMAT0019022	na	na
841_	hsa-mir-4489	MI0016850	hsa-miR-4489-5p	MIMAT0019023	na	na
842_	hsa-mir-4490	MI0016852	na	na	hsa-miR-4490-3p	MIMAT0019025
843_	hsa-mir-4491	MI0016853	na	na	hsa-miR-4491-3p	MIMAT0019026
844_	hsa-mir-4492	MI0016854	na	na	hsa-miR-4492-3p	MIMAT0019027
845_	hsa-mir-4493	MI0016855	na	na	hsa-miR-4493-3p	MIMAT0019028
846_	hsa-mir-4494	MI0016856	na	na	hsa-miR-4494-3p	MIMAT0019029
847_	hsa-mir-4495	MI0016857	hsa-miR-4495-5p	MIMAT0019030	na	na
848_	hsa-mir-4496	MI0016858	na	na	hsa-miR-4496-3p	MIMAT0019031
849_	hsa-mir-4497	MI0016859	hsa-miR-4497-5p	MIMAT0019032	na	na
850_	hsa-mir-4498	MI0016860	hsa-miR-4498-5p	MIMAT0019033	na	na
851_	hsa-mir-4499	MI0016862	hsa-miR-4499-5p	MIMAT0019035	na	na

852_	hsa-mir-449a	MI0001648	hsa-miR-449a-5p	MIMAT0001541	na	na
853_	hsa-mir-449b	MI0003673	hsa-miR-449b-5p	MIMAT0003327	hsa-miR-449b-3p	MIMAT0009203
854_	hsa-mir-449c	MI0003823	hsa-miR-449c-5p	MIMAT0010251	hsa-miR-449c-3p	MIMAT0013771
855_	hsa-mir-4500	MI0016863	na	na	hsa-miR-4500-3p	MIMAT0019036
856_	hsa-mir-4501	MI0016864	hsa-miR-4501-5p	MIMAT0019037	na	na
857_	hsa-mir-4502	MI0016865	na	na	hsa-miR-4502-3p	MIMAT0019038
858_	hsa-mir-4503	MI0016866	hsa-miR-4503-5p	MIMAT0019039	na	na
859_	hsa-mir-4504	MI0016867	na	na	hsa-miR-4504-3p	MIMAT0019040
860_	hsa-mir-4505	MI0016868	hsa-miR-4505-5p	MIMAT0019041	na	na
861_	hsa-mir-4506	MI0016869	na	na	hsa-miR-4506-3p	MIMAT0019042
862_	hsa-mir-4507	MI0016871	na	na	hsa-miR-4507-3p	MIMAT0019044
863_	hsa-mir-4508	MI0016872	hsa-miR-4508-5p	MIMAT0019045	na	na
864_	hsa-mir-4509-1	MI0016873	hsa-miR-4509-5p	MIMAT0019046	na	na
865_	hsa-mir-4509-2	MI0016874	hsa-miR-4509-5p	MIMAT0019046	na	na
866_	hsa-mir-4509-3	MI0016875	hsa-miR-4509-5p	MIMAT0019046	na	na
867_	hsa-mir-450a-1	MI0001652	hsa-miR-450a-5p	MIMAT0001545	hsa-miR-450a-1-3p	MIMAT0022700
868_	hsa-mir-450a-2	MI0003187	hsa-miR-450a-5p	MIMAT0001545	hsa-miR-450a-2-3p	MIMAT0031074
869_	hsa-mir-450b	MI0005531	hsa-miR-450b-5p	MIMAT0004909	hsa-miR-450b-3p	MIMAT0004910
870_	hsa-mir-4510	MI0016876	hsa-miR-4510-5p	MIMAT0019047	na	na
871_	hsa-mir-4511	MI0016877	hsa-miR-4511-5p	MIMAT0019048	na	na
872_	hsa-mir-4512	MI0016878	na	na	hsa-miR-4512-3p	MIMAT0019049
873_	hsa-mir-4513	MI0016879	hsa-miR-4513-5p	MIMAT0019050	na	na
874_	hsa-mir-4514	MI0016880	hsa-miR-4514-5p	MIMAT0019051	na	na
875_	hsa-mir-4515	MI0016881	hsa-miR-4515-5p	MIMAT0019052	na	na
876_	hsa-mir-4516	MI0016882	hsa-miR-4516-5p	MIMAT0019053	na	na
877_	hsa-mir-4517	MI0016883	hsa-miR-4517-5p	MIMAT0019054	na	na
878_	hsa-mir-4518	MI0016884	na	na	hsa-miR-4518-3p	MIMAT0019055
879_	hsa-mir-4519	MI0016885	hsa-miR-4519-5p	MIMAT0019056	na	na

880_	hsa-mir-451a	MI0001729	hsa-miR-451a-5p	MIMAT0001631	na	na
881_	hsa-mir-451b	MI0017360	hsa-miR-451b-5p	MIMAT0019840	na	na
882_	hsa-mir-452	MI0001733	hsa-miR-452-5p	MIMAT0001635	hsa-miR-452-3p	MIMAT0001636
883_	hsa-mir-4520a	MI0016886	hsa-miR-4520a-5p	MIMAT0019235	hsa-miR-4520a-3p	MIMAT0019057
884_	hsa-mir-4520b	MI0017358	hsa-miR-4520b-5p	MIMAT0020299	hsa-miR-4520b-3p	MIMAT0020300
885_	hsa-mir-4521	MI0016887	hsa-miR-4521-5p	MIMAT0019058	na	na
886_	hsa-mir-4522	MI0016889	na	na	hsa-miR-4522-3p	MIMAT0019060
887_	hsa-mir-4523	MI0016890	hsa-miR-4523-5p	MIMAT0019061	na	na
888_	hsa-mir-4524a	MI0016891	hsa-miR-4524a-5p	MIMAT0019062	hsa-miR-4524a-3p	MIMAT0019063
889_	hsa-mir-4524b	MI0019114	hsa-miR-4524b-5p	MIMAT0022255	hsa-miR-4524b-3p	MIMAT0022256
890_	hsa-mir-4525	MI0016892	hsa-miR-4525-5p	MIMAT0019064	na	na
891_	hsa-mir-4526	MI0016893	na	na	hsa-miR-4526-3p	MIMAT0019065
892_	hsa-mir-4527	MI0016894	hsa-miR-4527-5p	MIMAT0019066	na	na
893_	hsa-mir-4528	MI0016895	na	na	hsa-miR-4528-3p	MIMAT0019067
894_	hsa-mir-4529	MI0016896	hsa-miR-4529-5p	MIMAT0019236	hsa-miR-4529-3p	MIMAT0019068
895_	hsa-mir-4530	MI0016897	na	na	hsa-miR-4530-3p	MIMAT0019069
896_	hsa-mir-4531	MI0016898	na	na	hsa-miR-4531-3p	MIMAT0019070
897_	hsa-mir-4532	MI0016899	hsa-miR-4532-5p	MIMAT0019071	na	na
898_	hsa-mir-4533	MI0016900	hsa-miR-4533-5p	MIMAT0019072	na	na
899_	hsa-mir-4534	MI0016901	na	na	hsa-miR-4534-3p	MIMAT0019073
900_	hsa-mir-4535	MI0016903	na	na	hsa-miR-4535-3p	MIMAT0019075
901_	hsa-mir-4536-1	MI0016906	hsa-miR-4536-5p	MIMAT0019078	hsa-miR-4536-3p	MIMAT0020959
902_	hsa-mir-4536-2	MI0019149	hsa-miR-4536-5p	MIMAT0019078	hsa-miR-4536-3p	MIMAT0020959
903_	hsa-mir-4537	MI0016908	hsa-miR-4537-5p	MIMAT0019080	na	na
904_	hsa-mir-4538	MI0016909	hsa-miR-4538-5p	MIMAT0019081	na	na
905_	hsa-mir-4539	MI0016910	na	na	hsa-miR-4539-3p	MIMAT0019082
906_	hsa-mir-454	MI0003820	hsa-miR-454-5p	MIMAT0003884	hsa-miR-454-3p	MIMAT0003885
907_	hsa-mir-4540	MI0016911	na	na	hsa-miR-4540-3p	MIMAT0019083

908_	hsa-mir-455	MI0003513	hsa-miR-455-5p	MIMAT0003150	hsa-miR-455-3p	MIMAT0004784
909_	hsa-mir-4632	MI0017259	hsa-miR-4632-5p	MIMAT0022977	hsa-miR-4632-3p	MIMAT0019688
910_	hsa-mir-4633	MI0017260	hsa-miR-4633-5p	MIMAT0019689	hsa-miR-4633-3p	MIMAT0019690
911_	hsa-mir-4634	MI0017261	hsa-miR-4634-5p	MIMAT0019691	na	na
912_	hsa-mir-4635	MI0017262	na	na	hsa-miR-4635-3p	MIMAT0019692
913_	hsa-mir-4636	MI0017263	hsa-miR-4636-5p	MIMAT0019693	na	na
914_	hsa-mir-4637	MI0017264	na	na	hsa-miR-4637-3p	MIMAT0019694
915_	hsa-mir-4638	MI0017265	hsa-miR-4638-5p	MIMAT0019695	hsa-miR-4638-3p	MIMAT0019696
916_	hsa-mir-4639	MI0017266	hsa-miR-4639-5p	MIMAT0019697	hsa-miR-4639-3p	MIMAT0019698
917_	hsa-mir-4640	MI0017267	hsa-miR-4640-5p	MIMAT0019699	hsa-miR-4640-3p	MIMAT0019700
918_	hsa-mir-4641	MI0017268	na	na	hsa-miR-4641-3p	MIMAT0019701
919_	hsa-mir-4642	MI0017269	hsa-miR-4642-5p	MIMAT0019702	na	na
920_	hsa-mir-4643	MI0017270	na	na	hsa-miR-4643-3p	MIMAT0019703
921_	hsa-mir-4644	MI0017271	na	na	hsa-miR-4644-3p	MIMAT0019704
922_	hsa-mir-4645	MI0017272	hsa-miR-4645-5p	MIMAT0019705	hsa-miR-4645-3p	MIMAT0019706
923_	hsa-mir-4646	MI0017273	hsa-miR-4646-5p	MIMAT0019707	hsa-miR-4646-3p	MIMAT0019708
924_	hsa-mir-4647	MI0017274	hsa-miR-4647-5p	MIMAT0019709	na	na
925_	hsa-mir-4648	MI0017275	hsa-miR-4648-5p	MIMAT0019710	na	na
926_	hsa-mir-4649	MI0017276	hsa-miR-4649-5p	MIMAT0019711	hsa-miR-4649-3p	MIMAT0019712
927_	hsa-mir-4650-1	MI0017277	hsa-miR-4650-5p	MIMAT0019713	hsa-miR-4650-3p	MIMAT0019714
928_	hsa-mir-4650-2	MI0017278	hsa-miR-4650-5p	MIMAT0019713	hsa-miR-4650-3p	MIMAT0019714
929_	hsa-mir-4651	MI0017279	hsa-miR-4651-5p	MIMAT0019715	na	na
930_	hsa-mir-4652	MI0017280	hsa-miR-4652-5p	MIMAT0019716	hsa-miR-4652-3p	MIMAT0019717
931_	hsa-mir-4653	MI0017281	hsa-miR-4653-5p	MIMAT0019718	hsa-miR-4653-3p	MIMAT0019719
932_	hsa-mir-4654	MI0017282	hsa-miR-4654-5p	MIMAT0019720	na	na
933_	hsa-mir-4655	MI0017283	hsa-miR-4655-5p	MIMAT0019721	hsa-miR-4655-3p	MIMAT0019722
934_	hsa-mir-4656	MI0017284	hsa-miR-4656-5p	MIMAT0019723	na	na
935_	hsa-mir-4657	MI0017285	hsa-miR-4657-5p	MIMAT0019724	na	na

936_	hsa-mir-4658	MI0017286	na	na	hsa-miR-4658-3p	MIMAT0019725
937_	hsa-mir-4659a	MI0017287	hsa-miR-4659a-5p	MIMAT0019726	hsa-miR-4659a-3p	MIMAT0019727
938_	hsa-mir-4659b	MI0017291	hsa-miR-4659b-5p	MIMAT0019733	hsa-miR-4659b-3p	MIMAT0019734
939_	hsa-mir-466	MI0014157	na	na	hsa-miR-466-3p	MIMAT0015002
940_	hsa-mir-4660	MI0017288	hsa-miR-4660-5p	MIMAT0019728	na	na
941_	hsa-mir-4661	MI0017289	hsa-miR-4661-5p	MIMAT0019729	hsa-miR-4661-3p	MIMAT0019730
942_	hsa-mir-4662a	MI0017290	hsa-miR-4662a-5p	MIMAT0019731	hsa-miR-4662a-3p	MIMAT0019732
943_	hsa-mir-4662b	MI0017293	na	na	hsa-miR-4662b-3p	MIMAT0019736
944_	hsa-mir-4663	MI0017292	hsa-miR-4663-5p	MIMAT0019735	na	na
945_	hsa-mir-4664	MI0017294	hsa-miR-4664-5p	MIMAT0019737	hsa-miR-4664-3p	MIMAT0019738
946_	hsa-mir-4665	MI0017295	hsa-miR-4665-5p	MIMAT0019739	hsa-miR-4665-3p	MIMAT0019740
947_	hsa-mir-4666a	MI0017296	hsa-miR-4666a-5p	MIMAT0019741	hsa-miR-4666a-3p	MIMAT0019742
948_	hsa-mir-4666b	MI0019299	hsa-miR-4666b-5p	MIMAT0022485	na	na
949_	hsa-mir-4667	MI0017297	hsa-miR-4667-5p	MIMAT0019743	hsa-miR-4667-3p	MIMAT0019744
950_	hsa-mir-4668	MI0017298	hsa-miR-4668-5p	MIMAT0019745	hsa-miR-4668-3p	MIMAT0019746
951_	hsa-mir-4669	MI0017300	na	na	hsa-miR-4669-3p	MIMAT0019749
952_	hsa-mir-4670	MI0017301	hsa-miR-4670-5p	MIMAT0019750	hsa-miR-4670-3p	MIMAT0019751
953_	hsa-mir-4671	MI0017302	hsa-miR-4671-5p	MIMAT0019752	hsa-miR-4671-3p	MIMAT0019753
954_	hsa-mir-4672	MI0017303	na	na	hsa-miR-4672-3p	MIMAT0019754
955_	hsa-mir-4673	MI0017304	hsa-miR-4673-5p	MIMAT0019755	na	na
956_	hsa-mir-4674	MI0017305	na	na	hsa-miR-4674-3p	MIMAT0019756
957_	hsa-mir-4675	MI0017306	na	na	hsa-miR-4675-3p	MIMAT0019757
958_	hsa-mir-4676	MI0017307	hsa-miR-4676-5p	MIMAT0019758	hsa-miR-4676-3p	MIMAT0019759
959_	hsa-mir-4677	MI0017308	hsa-miR-4677-5p	MIMAT0019760	hsa-miR-4677-3p	MIMAT0019761
960_	hsa-mir-4678	MI0017309	hsa-miR-4678-5p	MIMAT0019762	na	na
961_	hsa-mir-4679-1	MI0017310	hsa-miR-4679-5p	MIMAT0019763	na	na
962_	hsa-mir-4679-2	MI0017311	hsa-miR-4679-5p	MIMAT0019763	na	na
963_	hsa-mir-4680	MI0017312	hsa-miR-4680-5p	MIMAT0019764	hsa-miR-4680-3p	MIMAT0019765

964_	hsa-mir-4681	MI0017313	hsa-miR-4681-5p	MIMAT0019766	na	na
965_	hsa-mir-4682	MI0017314	hsa-miR-4682-5p	MIMAT0019767	na	na
966_	hsa-mir-4683	MI0017315	na	na	hsa-miR-4683-3p	MIMAT0019768
967_	hsa-mir-4684	MI0017316	hsa-miR-4684-5p	MIMAT0019769	hsa-miR-4684-3p	MIMAT0019770
968_	hsa-mir-4685	MI0017317	hsa-miR-4685-5p	MIMAT0019771	hsa-miR-4685-3p	MIMAT0019772
969_	hsa-mir-4686	MI0017318	hsa-miR-4686-5p	MIMAT0019773	na	na
970_	hsa-mir-4687	MI0017319	hsa-miR-4687-5p	MIMAT0019774	hsa-miR-4687-3p	MIMAT0019775
971_	hsa-mir-4688	MI0017321	na	na	hsa-miR-4688-3p	MIMAT0019777
972_	hsa-mir-4689	MI0017322	hsa-miR-4689-5p	MIMAT0019778	na	na
973_	hsa-mir-4690	MI0017323	hsa-miR-4690-5p	MIMAT0019779	hsa-miR-4690-3p	MIMAT0019780
974_	hsa-mir-4691	MI0017324	hsa-miR-4691-5p	MIMAT0019781	hsa-miR-4691-3p	MIMAT0019782
975_	hsa-mir-4692	MI0017325	na	na	hsa-miR-4692-3p	MIMAT0019783
976_	hsa-mir-4693	MI0017326	hsa-miR-4693-5p	MIMAT0019784	hsa-miR-4693-3p	MIMAT0019785
977_	hsa-mir-4694	MI0017327	hsa-miR-4694-5p	MIMAT0019786	hsa-miR-4694-3p	MIMAT0019787
978_	hsa-mir-4695	MI0017328	hsa-miR-4695-5p	MIMAT0019788	hsa-miR-4695-3p	MIMAT0019789
979_	hsa-mir-4696	MI0017329	hsa-miR-4696-5p	MIMAT0019790	na	na
980_	hsa-mir-4697	MI0017330	hsa-miR-4697-5p	MIMAT0019791	hsa-miR-4697-3p	MIMAT0019792
981_	hsa-mir-4698	MI0017331	na	na	hsa-miR-4698-3p	MIMAT0019793
982_	hsa-mir-4699	MI0017332	hsa-miR-4699-5p	MIMAT0019794	hsa-miR-4699-3p	MIMAT0019795
983_	hsa-mir-4700	MI0017333	hsa-miR-4700-5p	MIMAT0019796	hsa-miR-4700-3p	MIMAT0019797
984_	hsa-mir-4701	MI0017334	hsa-miR-4701-5p	MIMAT0019798	hsa-miR-4701-3p	MIMAT0019799
985_	hsa-mir-4703	MI0017336	hsa-miR-4703-5p	MIMAT0019801	hsa-miR-4703-3p	MIMAT0019802
986_	hsa-mir-4704	MI0017337	hsa-miR-4704-5p	MIMAT0019803	hsa-miR-4704-3p	MIMAT0019804
987_	hsa-mir-4705	MI0017338	hsa-miR-4705-5p	MIMAT0019805	na	na
988_	hsa-mir-4706	MI0017339	hsa-miR-4706-5p	MIMAT0019806	na	na
989_	hsa-mir-4707	MI0017340	hsa-miR-4707-5p	MIMAT0019807	hsa-miR-4707-3p	MIMAT0019808
990_	hsa-mir-4708	MI0017341	hsa-miR-4708-5p	MIMAT0019809	hsa-miR-4708-3p	MIMAT0019810
991_	hsa-mir-4709	MI0017342	hsa-miR-4709-5p	MIMAT0019811	hsa-miR-4709-3p	MIMAT0019812

992_	hsa-mir-4710	MI0017344	hsa-miR-4710-5p	MIMAT0019815	na	na
993_	hsa-mir-4711	MI0017345	hsa-miR-4711-5p	MIMAT0019816	hsa-miR-4711-3p	MIMAT0019817
994_	hsa-mir-4712	MI0017346	hsa-miR-4712-5p	MIMAT0019818	hsa-miR-4712-3p	MIMAT0019819
995_	hsa-mir-4713	MI0017347	hsa-miR-4713-5p	MIMAT0019820	hsa-miR-4713-3p	MIMAT0019821
996_	hsa-mir-4714	MI0017348	hsa-miR-4714-5p	MIMAT0019822	hsa-miR-4714-3p	MIMAT0019823
997_	hsa-mir-4715	MI0017349	hsa-miR-4715-5p	MIMAT0019824	hsa-miR-4715-3p	MIMAT0019825
998_	hsa-mir-4716	MI0017350	hsa-miR-4716-5p	MIMAT0019826	hsa-miR-4716-3p	MIMAT0019827
999_	hsa-mir-4717	MI0017352	hsa-miR-4717-5p	MIMAT0019829	hsa-miR-4717-3p	MIMAT0019830
1000_	hsa-mir-4718	MI0017353	hsa-miR-4718-5p	MIMAT0019831	na	na
1001_	hsa-mir-4719	MI0017354	na	na	hsa-miR-4719-3p	MIMAT0019832
1002_	hsa-mir-4720	MI0017355	hsa-miR-4720-5p	MIMAT0019833	hsa-miR-4720-3p	MIMAT0019834
1003_	hsa-mir-4721	MI0017356	na	na	hsa-miR-4721-3p	MIMAT0019835
1004_	hsa-mir-4722	MI0017357	hsa-miR-4722-5p	MIMAT0019836	hsa-miR-4722-3p	MIMAT0019837
1005_	hsa-mir-4723	MI0017359	hsa-miR-4723-5p	MIMAT0019838	hsa-miR-4723-3p	MIMAT0019839
1006_	hsa-mir-4724	MI0017361	hsa-miR-4724-5p	MIMAT0019841	hsa-miR-4724-3p	MIMAT0019842
1007_	hsa-mir-4725	MI0017362	hsa-miR-4725-5p	MIMAT0019843	hsa-miR-4725-3p	MIMAT0019844
1008_	hsa-mir-4726	MI0017363	hsa-miR-4726-5p	MIMAT0019845	hsa-miR-4726-3p	MIMAT0019846
1009_	hsa-mir-4727	MI0017364	hsa-miR-4727-5p	MIMAT0019847	hsa-miR-4727-3p	MIMAT0019848
1010_	hsa-mir-4728	MI0017365	hsa-miR-4728-5p	MIMAT0019849	hsa-miR-4728-3p	MIMAT0019850
1011_	hsa-mir-4729	MI0017366	hsa-miR-4729-5p	MIMAT0019851	na	na
1012_	hsa-mir-4730	MI0017367	hsa-miR-4730-5p	MIMAT0019852	na	na
1013_	hsa-mir-4731	MI0017368	hsa-miR-4731-5p	MIMAT0019853	hsa-miR-4731-3p	MIMAT0019854
1014_	hsa-mir-4732	MI0017369	hsa-miR-4732-5p	MIMAT0019855	hsa-miR-4732-3p	MIMAT0019856
1015_	hsa-mir-4733	MI0017370	hsa-miR-4733-5p	MIMAT0019857	hsa-miR-4733-3p	MIMAT0019858
1016_	hsa-mir-4734	MI0017371	na	na	hsa-miR-4734-3p	MIMAT0019859
1017_	hsa-mir-4735	MI0017372	hsa-miR-4735-5p	MIMAT0019860	hsa-miR-4735-3p	MIMAT0019861
1018_	hsa-mir-4736	MI0017373	hsa-miR-4736-5p	MIMAT0019862	na	na
1019_	hsa-mir-4737	MI0017374	hsa-miR-4737-5p	MIMAT0019863	na	na

1020_	hsa-mir-4738	MI0017376	hsa-miR-4738-5p	MIMAT0019866	hsa-miR-4738-3p	MIMAT0019867
1021_	hsa-mir-4739	MI0017377	hsa-miR-4739-5p	MIMAT0019868	na	na
1022_	hsa-mir-4740	MI0017378	hsa-miR-4740-5p	MIMAT0019869	hsa-miR-4740-3p	MIMAT0019870
1023_	hsa-mir-4741	MI0017379	na	na	hsa-miR-4741-3p	MIMAT0019871
1024_	hsa-mir-4742	MI0017380	hsa-miR-4742-5p	MIMAT0019872	hsa-miR-4742-3p	MIMAT0019873
1025_	hsa-mir-4743	MI0017381	hsa-miR-4743-5p	MIMAT0019874	hsa-miR-4743-3p	MIMAT0022978
1026_	hsa-mir-4744	MI0017382	hsa-miR-4744-5p	MIMAT0019875	na	na
1027_	hsa-mir-4745	MI0017384	hsa-miR-4745-5p	MIMAT0019878	hsa-miR-4745-3p	MIMAT0019879
1028_	hsa-mir-4746	MI0017385	hsa-miR-4746-5p	MIMAT0019880	hsa-miR-4746-3p	MIMAT0019881
1029_	hsa-mir-4747	MI0017386	hsa-miR-4747-5p	MIMAT0019882	hsa-miR-4747-3p	MIMAT0019883
1030_	hsa-mir-4748	MI0017387	hsa-miR-4748-5p	MIMAT0019884	na	na
1031_	hsa-mir-4749	MI0017388	hsa-miR-4749-5p	MIMAT0019885	hsa-miR-4749-3p	MIMAT0019886
1032_	hsa-mir-4750	MI0017389	hsa-miR-4750-5p	MIMAT0019887	hsa-miR-4750-3p	MIMAT0022979
1033_	hsa-mir-4751	MI0017390	hsa-miR-4751-5p	MIMAT0019888	na	na
1034_	hsa-mir-4752	MI0017391	hsa-miR-4752-5p	MIMAT0019889	na	na
1035_	hsa-mir-4753	MI0017392	hsa-miR-4753-5p	MIMAT0019890	hsa-miR-4753-3p	MIMAT0019891
1036_	hsa-mir-4754	MI0017394	hsa-miR-4754-5p	MIMAT0019894	na	na
1037_	hsa-mir-4755	MI0017395	hsa-miR-4755-5p	MIMAT0019895	hsa-miR-4755-3p	MIMAT0019896
1038_	hsa-mir-4756	MI0017397	hsa-miR-4756-5p	MIMAT0019899	hsa-miR-4756-3p	MIMAT0019900
1039_	hsa-mir-4757	MI0017398	hsa-miR-4757-5p	MIMAT0019901	hsa-miR-4757-3p	MIMAT0019902
1040_	hsa-mir-4758	MI0017399	hsa-miR-4758-5p	MIMAT0019903	hsa-miR-4758-3p	MIMAT0019904
1041_	hsa-mir-4759	MI0017400	hsa-miR-4759-5p	MIMAT0019905	na	na
1042_	hsa-mir-4760	MI0017401	hsa-miR-4760-5p	MIMAT0019906	hsa-miR-4760-3p	MIMAT0019907
1043_	hsa-mir-4761	MI0017402	hsa-miR-4761-5p	MIMAT0019908	hsa-miR-4761-3p	MIMAT0019909
1044_	hsa-mir-4762	MI0017403	hsa-miR-4762-5p	MIMAT0019910	hsa-miR-4762-3p	MIMAT0019911
1045_	hsa-mir-4763	MI0017404	hsa-miR-4763-5p	MIMAT0019912	hsa-miR-4763-3p	MIMAT0019913
1046_	hsa-mir-4764	MI0017405	hsa-miR-4764-5p	MIMAT0019914	hsa-miR-4764-3p	MIMAT0019915
1047_	hsa-mir-4765	MI0017406	na	na	hsa-miR-4765-3p	MIMAT0019916

1048_	hsa-mir-4766	MI0017407	hsa-miR-4766-5p	MIMAT0019917	hsa-miR-4766-3p	MIMAT0019918
1049_	hsa-mir-4767	MI0017408	hsa-miR-4767-5p	MIMAT0019919	na	na
1050_	hsa-mir-4768	MI0017409	hsa-miR-4768-5p	MIMAT0019920	hsa-miR-4768-3p	MIMAT0019921
1051_	hsa-mir-4769	MI0017410	hsa-miR-4769-5p	MIMAT0019922	hsa-miR-4769-3p	MIMAT0019923
1052_	hsa-mir-4770	MI0017411	na	na	hsa-miR-4770-3p	MIMAT0019924
1053_	hsa-mir-4771-1	MI0017412	na	na	hsa-miR-4771-3p	MIMAT0019925
1054_	hsa-mir-4771-2	MI0017413	na	na	hsa-miR-4771-3p	MIMAT0019925
1055_	hsa-mir-4772	MI0017414	hsa-miR-4772-5p	MIMAT0019926	hsa-miR-4772-3p	MIMAT0019927
1056_	hsa-mir-4773-1	MI0017415	na	na	hsa-miR-4773-3p	MIMAT0019928
1057_	hsa-mir-4773-2	MI0017416	na	na	hsa-miR-4773-3p	MIMAT0019928
1058_	hsa-mir-4774	MI0017417	hsa-miR-4774-5p	MIMAT0019929	hsa-miR-4774-3p	MIMAT0019930
1059_	hsa-mir-4775	MI0017418	hsa-miR-4775-5p	MIMAT0019931	na	na
1060_	hsa-mir-4776-1	MI0017419	hsa-miR-4776-5p	MIMAT0019932	hsa-miR-4776-3p	MIMAT0019933
1061_	hsa-mir-4776-2	MI0017420	hsa-miR-4776-5p	MIMAT0019932	hsa-miR-4776-3p	MIMAT0019933
1062_	hsa-mir-4777	MI0017421	hsa-miR-4777-5p	MIMAT0019934	hsa-miR-4777-3p	MIMAT0019935
1063_	hsa-mir-4778	MI0017422	hsa-miR-4778-5p	MIMAT0019936	hsa-miR-4778-3p	MIMAT0019937
1064_	hsa-mir-4779	MI0017423	na	na	hsa-miR-4779-3p	MIMAT0019938
1065_	hsa-mir-4780	MI0017424	na	na	hsa-miR-4780-3p	MIMAT0019939
1066_	hsa-mir-4781	MI0017426	hsa-miR-4781-5p	MIMAT0019942	hsa-miR-4781-3p	MIMAT0019943
1067_	hsa-mir-4782	MI0017427	hsa-miR-4782-5p	MIMAT0019944	hsa-miR-4782-3p	MIMAT0019945
1068_	hsa-mir-4783	MI0017428	hsa-miR-4783-5p	MIMAT0019946	hsa-miR-4783-3p	MIMAT0019947
1069_	hsa-mir-4784	MI0017429	hsa-miR-4784-5p	MIMAT0019948	na	na
1070_	hsa-mir-4785	MI0017430	na	na	hsa-miR-4785-3p	MIMAT0019949
1071_	hsa-mir-4786	MI0017433	hsa-miR-4786-5p	MIMAT0019954	hsa-miR-4786-3p	MIMAT0019955
1072_	hsa-mir-4787	MI0017434	hsa-miR-4787-5p	MIMAT0019956	hsa-miR-4787-3p	MIMAT0019957
1073_	hsa-mir-4788	MI0017435	hsa-miR-4788-5p	MIMAT0019958	na	na
1074_	hsa-mir-4789	MI0017436	hsa-miR-4789-5p	MIMAT0019959	hsa-miR-4789-3p	MIMAT0019960
1075_	hsa-mir-4790	MI0017437	hsa-miR-4790-5p	MIMAT0019961	hsa-miR-4790-3p	MIMAT0019962
1076_	hsa-mir-4791	MI0017438	hsa-miR-4791-5p	MIMAT0019963	na	na

1077_	hsa-mir-4792	MI0017439	hsa-miR-4792-5p	MIMAT0019964	na	na
1078_	hsa-mir-4793	MI0017440	hsa-miR-4793-5p	MIMAT0019965	hsa-miR-4793-3p	MIMAT0019966
1079_	hsa-mir-4794	MI0017441	hsa-miR-4794-5p	MIMAT0019967	na	na
1080_	hsa-mir-4795	MI0017442	hsa-miR-4795-5p	MIMAT0019968	hsa-miR-4795-3p	MIMAT0019969
1081_	hsa-mir-4796	MI0017443	hsa-miR-4796-5p	MIMAT0019970	hsa-miR-4796-3p	MIMAT0019971
1082_	hsa-mir-4797	MI0017444	hsa-miR-4797-5p	MIMAT0019972	hsa-miR-4797-3p	MIMAT0019973
1083_	hsa-mir-4798	MI0017445	hsa-miR-4798-5p	MIMAT0019974	hsa-miR-4798-3p	MIMAT0019975
1084_	hsa-mir-4799	MI0017446	hsa-miR-4799-5p	MIMAT0019976	hsa-miR-4799-3p	MIMAT0019977
1085_	hsa-mir-4800	MI0017448	hsa-miR-4800-5p	MIMAT0019978	hsa-miR-4800-3p	MIMAT0019979
1086_	hsa-mir-4801	MI0017449	na	na	hsa-miR-4801-3p	MIMAT0019980
1087_	hsa-mir-4802	MI0017450	hsa-miR-4802-5p	MIMAT0019981	hsa-miR-4802-3p	MIMAT0019982
1088_	hsa-mir-4803	MI0017451	hsa-miR-4803-5p	MIMAT0019983	na	na
1089_	hsa-mir-4804	MI0017452	hsa-miR-4804-5p	MIMAT0019984	hsa-miR-4804-3p	MIMAT0019985
1090_	hsa-mir-483	MI0002467	hsa-miR-483-5p	MIMAT0004761	hsa-miR-483-3p	MIMAT0002173
1091_	hsa-mir-484	MI0002468	hsa-miR-484-5p	MIMAT0002174	na	na
1092_	hsa-mir-485	MI0002469	hsa-miR-485-5p	MIMAT0002175	hsa-miR-485-3p	MIMAT0002176
1093_	hsa-mir-486	MI0002470	hsa-miR-486-5p	MIMAT0002177	hsa-miR-486-3p	MIMAT0004762
1094_	hsa-mir-486-2	MI0023622	hsa-miR-486-5p	MIMAT0002177	hsa-miR-486-3p	MIMAT0004762
1095_	hsa-mir-487a	MI0002471	hsa-miR-487a-5p	MIMAT0026559	hsa-miR-487a-3p	MIMAT0002178
1096_	hsa-mir-487b	MI0003530	hsa-miR-487b-5p	MIMAT0026614	hsa-miR-487b-3p	MIMAT0003180
1097_	hsa-mir-488	MI0003123	hsa-miR-488-5p	MIMAT0002804	hsa-miR-488-3p	MIMAT0004763
1098_	hsa-mir-489	MI0003124	hsa-miR-489-5p	MIMAT0026605	hsa-miR-489-3p	MIMAT0002805
1099_	hsa-mir-490	MI0003125	hsa-miR-490-5p	MIMAT0004764	hsa-miR-490-3p	MIMAT0002806
1100_	hsa-mir-491	MI0003126	hsa-miR-491-5p	MIMAT0002807	hsa-miR-491-3p	MIMAT0004765
1101_	hsa-mir-492	MI0003131	hsa-miR-492-5p	MIMAT0002812	na	na
1102_	hsa-mir-493	MI0003132	hsa-miR-493-5p	MIMAT0002813	hsa-miR-493-3p	MIMAT0003161
1103_	hsa-mir-494	MI0003134	hsa-miR-494-5p	MIMAT0026607	hsa-miR-494-3p	MIMAT0002816
1104_	hsa-mir-495	MI0003135	hsa-miR-495-5p	MIMAT0022924	hsa-miR-495-3p	MIMAT0002817
1105_	hsa-mir-496	MI0003136	na	na	hsa-miR-496-3p	MIMAT0002818

1106_	hsa-mir-497	MI0003138	hsa-miR-497-5p	MIMAT0002820	hsa-miR-497-3p	MIMAT0004768
1107_	hsa-mir-498	MI0003142	hsa-miR-498-5p	MIMAT0002824	na	na
1108_	hsa-mir-4999	MI0017865	hsa-miR-4999-5p	MIMAT0021017	hsa-miR-4999-3p	MIMAT0021018
1109_	hsa-mir-499a	MI0003183	hsa-miR-499a-5p	MIMAT0002870	hsa-miR-499a-3p	MIMAT0004772
1110_	hsa-mir-499b	MI0017396	hsa-miR-499b-5p	MIMAT0019897	hsa-miR-499b-3p	MIMAT0019898
1111_	hsa-mir-5000	MI0017866	hsa-miR-5000-5p	MIMAT0021019	hsa-miR-5000-3p	MIMAT0021020
1112_	hsa-mir-5001	MI0017867	hsa-miR-5001-5p	MIMAT0021021	hsa-miR-5001-3p	MIMAT0021022
1113_	hsa-mir-5002	MI0017868	hsa-miR-5002-5p	MIMAT0021023	hsa-miR-5002-3p	MIMAT0021024
1114_	hsa-mir-5003	MI0017869	hsa-miR-5003-5p	MIMAT0021025	hsa-miR-5003-3p	MIMAT0021026
1115_	hsa-mir-5004	MI0017870	hsa-miR-5004-5p	MIMAT0021027	hsa-miR-5004-3p	MIMAT0021028
1116_	hsa-mir-5006	MI0017873	hsa-miR-5006-5p	MIMAT0021033	hsa-miR-5006-3p	MIMAT0021034
1117_	hsa-mir-5007	MI0017874	hsa-miR-5007-5p	MIMAT0021035	hsa-miR-5007-3p	MIMAT0021036
1118_	hsa-mir-5008	MI0017876	hsa-miR-5008-5p	MIMAT0021039	hsa-miR-5008-3p	MIMAT0021040
1119_	hsa-mir-5009	MI0017877	hsa-miR-5009-5p	MIMAT0021041	hsa-miR-5009-3p	MIMAT0021042
1120_	hsa-mir-500a	MI0003184	hsa-miR-500a-5p	MIMAT0004773	hsa-miR-500a-3p	MIMAT0002871
1121_	hsa-mir-500b	MI0015903	hsa-miR-500b-5p	MIMAT0016925	hsa-miR-500b-3p	MIMAT0027032
1122_	hsa-mir-501	MI0003185	hsa-miR-501-5p	MIMAT0002872	hsa-miR-501-3p	MIMAT0004774
1123_	hsa-mir-5010	MI0017878	hsa-miR-5010-5p	MIMAT0021043	hsa-miR-5010-3p	MIMAT0021044
1124_	hsa-mir-5011	MI0017879	hsa-miR-5011-5p	MIMAT0021045	hsa-miR-5011-3p	MIMAT0021046
1125_	hsa-mir-502	MI0003186	hsa-miR-502-5p	MIMAT0002873	hsa-miR-502-3p	MIMAT0004775
1126_	hsa-mir-503	MI0003188	hsa-miR-503-5p	MIMAT0002874	hsa-miR-503-3p	MIMAT0022925
1127_	hsa-mir-504	MI0003189	hsa-miR-504-5p	MIMAT0002875	hsa-miR-504-3p	MIMAT0026612
1128_	hsa-mir-5047	MI0017932	na	na	hsa-miR-5047-3p	MIMAT0020541
1129_	hsa-mir-505	MI0003190	hsa-miR-505-5p	MIMAT0004776	hsa-miR-505-3p	MIMAT0002876
1130_	hsa-mir-506	MI0003193	hsa-miR-506-5p	MIMAT0022701	hsa-miR-506-3p	MIMAT0002878
1131_	hsa-mir-507	MI0003194	na	na	hsa-miR-507-3p	MIMAT0002879
1132_	hsa-mir-508	MI0003195	hsa-miR-508-5p	MIMAT0004778	hsa-miR-508-3p	MIMAT0002880
1133_	hsa-mir-5087	MI0017976	hsa-miR-5087-5p	MIMAT0021079	na	na
1134_	hsa-mir-5088	MI0017977	hsa-miR-5088-5p	MIMAT0021080	hsa-miR-5088-3p	MIMAT0027041

1135_	hsa-mir-5089	MI0017978	hsa-miR-5089-5p	MIMAT0021081	hsa-miR-5089-3p	MIMAT0022984
1136_	hsa-mir-509-1	MI0003196	hsa-miR-509-5p	MIMAT0004779	hsa-miR-509-3p	MIMAT0002881
1137_	hsa-mir-509-2	MI0005530	hsa-miR-509-5p	MIMAT0004779	hsa-miR-509-3p	MIMAT0002881
1138_	hsa-mir-509-3	MI0005717	hsa-miR-509-3-5p	MIMAT0004975	hsa-miR-509-3p	MIMAT0002881
1139_	hsa-mir-5090	MI0017979	hsa-miR-5090-5p	MIMAT0021082	na	na
1140_	hsa-mir-5091	MI0017980	hsa-miR-5091-5p	MIMAT0021083	na	na
1141_	hsa-mir-5092	MI0017981	na	na	hsa-miR-5092-3p	MIMAT0021084
1142_	hsa-mir-5093	MI0017982	na	na	hsa-miR-5093-3p	MIMAT0021085
1143_	hsa-mir-5094	MI0017983	hsa-miR-5094-5p	MIMAT0021086	na	na
1144_	hsa-mir-5095	MI0018001	hsa-miR-5095-5p	MIMAT0020600	na	na
1145_	hsa-mir-5096	MI0018004	hsa-miR-5096-5p	MIMAT0020603	na	na
1146_	hsa-mir-510	MI0003197	hsa-miR-510-5p	MIMAT0002882	hsa-miR-510-3p	MIMAT0026613
1147_	hsa-mir-5100	MI0019116	na	na	hsa-miR-5100-3p	MIMAT0022259
1148_	hsa-mir-511	MI0003127	hsa-miR-511-5p	MIMAT0002808	hsa-miR-511-3p	MIMAT0026606
1149_	hsa-mir-512-1	MI0003140	hsa-miR-512-5p	MIMAT0002822	hsa-miR-512-3p	MIMAT0002823
1150_	hsa-mir-512-2	MI0003141	hsa-miR-512-5p	MIMAT0002822	hsa-miR-512-3p	MIMAT0002823
1151_	hsa-mir-513a-1	MI0003191	hsa-miR-513a-5p	MIMAT0002877	hsa-miR-513a-3p	MIMAT0004777
1152_	hsa-mir-513a-2	MI0003192	hsa-miR-513a-5p	MIMAT0002877	hsa-miR-513a-3p	MIMAT0004777
1153_	hsa-mir-513b	MI0006648	hsa-miR-513b-5p	MIMAT0005788	hsa-miR-513b-3p	MIMAT0026749
1154_	hsa-mir-513c	MI0006649	hsa-miR-513c-5p	MIMAT0005789	hsa-miR-513c-3p	MIMAT0022728
1155_	hsa-mir-514a-1	MI0003198	hsa-miR-514a-5p	MIMAT0022702	hsa-miR-514a-3p	MIMAT0002883
1156_	hsa-mir-514a-2	MI0003199	hsa-miR-514a-5p	MIMAT0022702	hsa-miR-514a-3p	MIMAT0002883
1157_	hsa-mir-514a-3	MI0003200	hsa-miR-514a-5p	MIMAT0022702	hsa-miR-514a-3p	MIMAT0002883
1158_	hsa-mir-514b	MI0014251	hsa-miR-514b-5p	MIMAT0015087	hsa-miR-514b-3p	MIMAT0015088
1159_	hsa-mir-515-1	MI0003144	hsa-miR-515-5p	MIMAT0002826	hsa-miR-515-3p	MIMAT0002827
1160_	hsa-mir-515-2	MI0003147	hsa-miR-515-5p	MIMAT0002826	hsa-miR-515-3p	MIMAT0002827
1161_	hsa-mir-516a-1	MI0003180	hsa-miR-516a-5p	MIMAT0004770	hsa-miR-516a-3p	MIMAT0006778
1162_	hsa-mir-516a-2	MI0003181	hsa-miR-516a-5p	MIMAT0004770	hsa-miR-516a-3p	MIMAT0006778
1163_	hsa-mir-516b-1	MI0003172	hsa-miR-516b-5p	MIMAT0002859	hsa-miR-516b-3p	MIMAT0002860

1164_	hsa-mir-516b-2	MI0003167	hsa-miR-516b-5p	MIMAT0002859	hsa-miR-516b-3p	MIMAT0002860
1165_	hsa-mir-517a	MI0003161	na	na	hsa-miR-517a-3p	MIMAT0002852
1166_	hsa-mir-517b	MI0003165	na	na	hsa-miR-517b-3p	MIMAT0002857
1167_	hsa-mir-517c	MI0003174	na	na	hsa-miR-517c-3p	MIMAT0002866
1168_	hsa-mir-5186	MI0018165	na	na	hsa-miR-5186-3p	MIMAT0021116
1169_	hsa-mir-5187	MI0018166	hsa-miR-5187-5p	MIMAT0021117	hsa-miR-5187-3p	MIMAT0021118
1170_	hsa-mir-5188	MI0018167	na	na	hsa-miR-5188-3p	MIMAT0021119
1171_	hsa-mir-5189	MI0018168	hsa-miR-5189-5p	MIMAT0021120	hsa-miR-5189-3p	MIMAT0027088
1172_	hsa-mir-518a-1	MI0003170	hsa-miR-518a-5p	MIMAT0005457	hsa-miR-518a-3p	MIMAT0002863
1173_	hsa-mir-518a-2	MI0003173	hsa-miR-518a-5p	MIMAT0005457	hsa-miR-518a-3p	MIMAT0002863
1174_	hsa-mir-518b	MI0003156	na	na	hsa-miR-518b-3p	MIMAT0002844
1175_	hsa-mir-518c	MI0003159	hsa-miR-518c-5p	MIMAT0002847	hsa-miR-518c-3p	MIMAT0002848
1176_	hsa-mir-518d	MI0003171	hsa-miR-518d-5p	MIMAT0005456	hsa-miR-518d-3p	MIMAT0002864
1177_	hsa-mir-518e	MI0003169	hsa-miR-518e-5p	MIMAT0005450	hsa-miR-518e-3p	MIMAT0002861
1178_	hsa-mir-518f	MI0003154	hsa-miR-518f-5p	MIMAT0002841	hsa-miR-518f-3p	MIMAT0002842
1179_	hsa-mir-5190	MI0018169	na	na	hsa-miR-5190-3p	MIMAT0021121
1180_	hsa-mir-5191	MI0018170	na	na	hsa-miR-5191-3p	MIMAT0021122
1181_	hsa-mir-5192	MI0018171	na	na	hsa-miR-5192-3p	MIMAT0021123
1182_	hsa-mir-5193	MI0018172	na	na	hsa-miR-5193-3p	MIMAT0021124
1183_	hsa-mir-5194	MI0018173	na	na	hsa-miR-5194-3p	MIMAT0021125
1184_	hsa-mir-5195	MI0018174	hsa-miR-5195-5p	MIMAT0021126	hsa-miR-5195-3p	MIMAT0021127
1185_	hsa-mir-5196	MI0018175	hsa-miR-5196-5p	MIMAT0021128	hsa-miR-5196-3p	MIMAT0021129
1186_	hsa-mir-5197	MI0018176	hsa-miR-5197-5p	MIMAT0021130	hsa-miR-5197-3p	MIMAT0021131
1187_	hsa-mir-519a-1	MI0003178	hsa-miR-519a-5p	MIMAT0005452	hsa-miR-519a-3p	MIMAT0002869
1188_	hsa-mir-519a-2	MI0003182	na	na	hsa-miR-519a-3p	MIMAT0002869
1189_	hsa-mir-519b	MI0003151	hsa-miR-519b-5p	MIMAT0005454	hsa-miR-519b-3p	MIMAT0002837
1190_	hsa-mir-519c	MI0003148	hsa-miR-519c-5p	MIMAT0002831	hsa-miR-519c-3p	MIMAT0002832
1191_	hsa-mir-519d	MI0003162	hsa-miR-519d-5p	MIMAT0026610	hsa-miR-519d-3p	MIMAT0002853
1192_	hsa-mir-519e	MI0003145	hsa-miR-519e-5p	MIMAT0002828	hsa-miR-519e-3p	MIMAT0002829

1193_	hsa-mir-520a	MI0003149	hsa-miR-520a-5p	MIMAT0002833	hsa-miR-520a-3p	MIMAT0002834
1194_	hsa-mir-520b	MI0003155	na	na	hsa-miR-520b-3p	MIMAT0002843
1195_	hsa-mir-520c	MI0003158	hsa-miR-520c-5p	MIMAT0005455	hsa-miR-520c-3p	MIMAT0002846
1196_	hsa-mir-520d	MI0003164	hsa-miR-520d-5p	MIMAT0002855	hsa-miR-520d-3p	MIMAT0002856
1197_	hsa-mir-520e	MI0003143	na	na	hsa-miR-520e-3p	MIMAT0002825
1198_	hsa-mir-520f	MI0003146	hsa-miR-520f-5p	MIMAT0026609	hsa-miR-520f-3p	MIMAT0002830
1199_	hsa-mir-520g	MI0003166	hsa-miR-520g-5p	MIMAT0026611	hsa-miR-520g-3p	MIMAT0002858
1200_	hsa-mir-520h	MI0003175	na	na	hsa-miR-520h-3p	MIMAT0002867
1201_	hsa-mir-521-1	MI0003176	na	na	hsa-miR-521-3p	MIMAT0002854
1202_	hsa-mir-521-2	MI0003163	na	na	hsa-miR-521-3p	MIMAT0002854
1203_	hsa-mir-522	MI0003177	hsa-miR-518e-5p	MIMAT0005450	hsa-miR-522-3p	MIMAT0002868
1204_	hsa-mir-523	MI0003153	hsa-miR-518e-5p	MIMAT0005450	hsa-miR-523-3p	MIMAT0002840
1205_	hsa-mir-524	MI0003160	hsa-miR-524-5p	MIMAT0002849	hsa-miR-524-3p	MIMAT0002850
1206_	hsa-mir-525	MI0003152	hsa-miR-525-5p	MIMAT0002838	hsa-miR-525-3p	MIMAT0002839
1207_	hsa-mir-526a-1	MI0003157	hsa-miR-526a-5p	MIMAT0002845	na	na
1208_	hsa-mir-526a-2	MI0003168	hsa-miR-526a-5p	MIMAT0002845	na	na
1209_	hsa-mir-526b	MI0003150	hsa-miR-526b-5p	MIMAT0002835	hsa-miR-526b-3p	MIMAT0002836
1210_	hsa-mir-527	MI0003179	hsa-miR-518a-5p	MIMAT0005457	na	na
1211_	hsa-mir-532	MI0003205	hsa-miR-532-5p	MIMAT0002888	hsa-miR-532-3p	MIMAT0004780
1212_	hsa-mir-539	MI0003514	hsa-miR-539-5p	MIMAT0003163	hsa-miR-539-3p	MIMAT0022705
1213_	hsa-mir-541	MI0005539	hsa-miR-541-5p	MIMAT0004919	hsa-miR-541-3p	MIMAT0004920
1214_	hsa-mir-542	MI0003686	hsa-miR-542-5p	MIMAT0003340	hsa-miR-542-3p	MIMAT0003389
1215_	hsa-mir-543	MI0005565	na	na	hsa-miR-543-3p	MIMAT0004954
1216_	hsa-mir-544a	MI0003515	na	na	hsa-miR-544a-3p	MIMAT0003164
1217_	hsa-mir-544b	MI0014159	na	na	hsa-miR-544b-3p	MIMAT0015004
1218_	hsa-mir-545	MI0003516	hsa-miR-545-5p	MIMAT0004785	hsa-miR-545-3p	MIMAT0003165
1219_	hsa-mir-548a-1	MI0003593	na	na	hsa-miR-548a-3p	MIMAT0003251
1220_	hsa-mir-548a-2	MI0003598	na	na	hsa-miR-548a-3p	MIMAT0003251
1221_	hsa-mir-548a-3	MI0003612	hsa-miR-548a-5p	MIMAT0004803	hsa-miR-548a-3p	MIMAT0003251

1222_	hsa-mir-548aa-1	MI0016689	na	na	hsa-miR-548aa-3p	MIMAT0018447
1223_	hsa-mir-548aa-2	MI0016690	na	na	hsa-miR-548aa-3p	MIMAT0018447
1224_	hsa-mir-548ab	MI0016752	hsa-miR-548ab-5p	MIMAT0018928	na	na
1225_	hsa-mir-548ac	MI0016762	na	na	hsa-miR-548ac-3p	MIMAT0018938
1226_	hsa-mir-548ad	MI0016770	na	na	hsa-miR-548ad-3p	MIMAT0018946
1227_	hsa-mir-548ae-1	MI0016779	na	na	hsa-miR-548ae-3p	MIMAT0018954
1228_	hsa-mir-548ae-2	MI0016780	na	na	hsa-miR-548ae-3p	MIMAT0018954
1229_	hsa-mir-548ag-1	MI0016793	hsa-miR-548ag-5p	MIMAT0018969	na	na
1230_	hsa-mir-548ag-2	MI0016794	hsa-miR-548ag-5p	MIMAT0018969	na	na
1231_	hsa-mir-548ah	MI0016796	hsa-miR-548ah-5p	MIMAT0018972	hsa-miR-548ah-3p	MIMAT0020957
1232_	hsa-mir-548ai	MI0016813	hsa-miR-548ai-5p	MIMAT0018989	na	na
1233_	hsa-mir-548aj-1	MI0016814	na	na	hsa-miR-548aj-3p	MIMAT0018990
1234_	hsa-mir-548aj-2	MI0016815	hsa-miR-548aj-5p	MIMAT0022739	hsa-miR-548aj-3p	MIMAT0018990
1235_	hsa-mir-548ak	MI0016840	hsa-miR-548ak-5p	MIMAT0019013	na	na
1236_	hsa-mir-548al	MI0016851	na	na	hsa-miR-548al-3p	MIMAT0019024
1237_	hsa-mir-548am	MI0016904	hsa-miR-548am-5p	MIMAT0022740	hsa-miR-548am-3p	MIMAT0019076
1238_	hsa-mir-548an	MI0016907	hsa-miR-548an-5p	MIMAT0019079	na	na
1239_	hsa-mir-548ao	MI0017871	hsa-miR-548ao-5p	MIMAT0021029	hsa-miR-548ao-3p	MIMAT0021030
1240_	hsa-mir-548ap	MI0017875	hsa-miR-548ap-5p	MIMAT0021037	hsa-miR-548ap-3p	MIMAT0021038
1241_	hsa-mir-548aq	MI0019130	hsa-miR-548aq-5p	MIMAT0022263	hsa-miR-548aq-3p	MIMAT0022264
1242_	hsa-mir-548ar	MI0019131	hsa-miR-548ar-5p	MIMAT0022265	hsa-miR-548ar-3p	MIMAT0022266
1243_	hsa-mir-548as	MI0019132	hsa-miR-548as-5p	MIMAT0022267	hsa-miR-548as-3p	MIMAT0022268
1244_	hsa-mir-548at	MI0019137	hsa-miR-548at-5p	MIMAT0022277	hsa-miR-548at-3p	MIMAT0022278
1245_	hsa-mir-548au	MI0019145	hsa-miR-548au-5p	MIMAT0022291	hsa-miR-548au-3p	MIMAT0022292
1246_	hsa-mir-548av	MI0019152	hsa-miR-548av-5p	MIMAT0022303	hsa-miR-548av-3p	MIMAT0022304
1247_	hsa-mir-548aw	MI0019283	hsa-miR-548aw-5p	MIMAT0022471	na	na
1248_	hsa-mir-548ax	MI0019286	hsa-miR-548ax-5p	MIMAT0022474	na	na
1249_	hsa-mir-548ay	MI0022210	hsa-miR-548ay-5p	MIMAT0025452	hsa-miR-548ay-3p	MIMAT0025453
1250_	hsa-mir-548az	MI0022212	hsa-miR-548az-5p	MIMAT0025456	hsa-miR-548az-3p	MIMAT0025457

1251_	hsa-mir-548b	MI0003596	hsa-miR-548b-5p	MIMAT0004798	hsa-miR-548b-3p	MIMAT0003254
1252_	hsa-mir-548ba	MI0025747	hsa-miR-548ba-5p	MIMAT0031175	na	na
1253_	hsa-mir-548c	MI0003630	hsa-miR-548c-5p	MIMAT0004806	hsa-miR-548c-3p	MIMAT0003285
1254_	hsa-mir-548d-1	MI0003668	hsa-miR-548d-5p	MIMAT0004812	hsa-miR-548d-3p	MIMAT0003323
1255_	hsa-mir-548d-2	MI0003671	hsa-miR-548d-5p	MIMAT0004812	hsa-miR-548d-3p	MIMAT0003323
1256_	hsa-mir-548e	MI0006344	hsa-miR-548e-5p	MIMAT0026736	hsa-miR-548e-3p	MIMAT0005874
1257_	hsa-mir-548f-1	MI0006374	hsa-miR-548f-5p	MIMAT0026739	hsa-miR-548f-3p	MIMAT0005895
1258_	hsa-mir-548f-2	MI0006375	na	na	hsa-miR-548f-3p	MIMAT0005895
1259_	hsa-mir-548f-3	MI0006376	na	na	hsa-miR-548f-3p	MIMAT0005895
1260_	hsa-mir-548f-4	MI0006377	na	na	hsa-miR-548f-3p	MIMAT0005895
1261_	hsa-mir-548f-5	MI0006378	na	na	hsa-miR-548f-3p	MIMAT0005895
1262_	hsa-mir-548g	MI0006395	hsa-miR-548g-5p	MIMAT0022722	hsa-miR-548g-3p	MIMAT0005912
1263_	hsa-mir-548h-1	MI0006411	hsa-miR-548h-5p	MIMAT0005928	na	na
1264_	hsa-mir-548h-2	MI0006412	hsa-miR-548h-5p	MIMAT0005928	na	na
1265_	hsa-mir-548h-3	MI0006413	hsa-miR-548h-5p	MIMAT0005928	na	na
1266_	hsa-mir-548h-4	MI0006414	hsa-miR-548h-5p	MIMAT0005928	hsa-miR-548h-3p	MIMAT0022723
1267_	hsa-mir-548h-5	MI0016751	hsa-miR-548h-5p	MIMAT0005928	na	na
1268_	hsa-mir-548i-1	MI0006421	hsa-miR-548i-5p	MIMAT0005935	na	na
1269_	hsa-mir-548i-2	MI0006422	hsa-miR-548i-5p	MIMAT0005935	na	na
1270_	hsa-mir-548i-3	MI0006423	hsa-miR-548i-5p	MIMAT0005935	na	na
1271_	hsa-mir-548i-4	MI0006424	hsa-miR-548i-5p	MIMAT0005935	na	na
1272_	hsa-mir-548j	MI0006345	hsa-miR-548j-5p	MIMAT0005875	hsa-miR-548j-3p	MIMAT0026737
1273_	hsa-mir-548k	MI0006354	hsa-miR-548k-5p	MIMAT0005882	na	na
1274_	hsa-mir-548l	MI0006361	hsa-miR-548l-5p	MIMAT0005889	na	na
1275_	hsa-mir-548m	MI0006400	hsa-miR-548m-5p	MIMAT0005917	na	na
1276_	hsa-mir-548n	MI0006399	hsa-miR-548n-5p	MIMAT0005916	na	na
1277_	hsa-mir-548o	MI0006402	na	na	hsa-miR-548o-3p	MIMAT0005919
1278_	hsa-mir-548o-2	MI0016746	hsa-miR-548o-5p	MIMAT0022738	hsa-miR-548o-3p	MIMAT0005919
1279_	hsa-mir-548p	MI0006420	na	na	hsa-miR-548p-3p	MIMAT0005934

1280_	hsa-mir-548q	MI0010637	hsa-miR-548q-5p	MIMAT0011163	na	na
1281_	hsa-mir-548s	MI0014141	na	na	hsa-miR-548s-3p	MIMAT0014987
1282_	hsa-mir-548t	MI0014164	hsa-miR-548t-5p	MIMAT0015009	hsa-miR-548t-3p	MIMAT0022730
1283_	hsa-mir-548u	MI0014168	na	na	hsa-miR-548u-3p	MIMAT0015013
1284_	hsa-mir-548v	MI0014174	na	na	hsa-miR-548v-3p	MIMAT0015020
1285_	hsa-mir-548w	MI0014222	hsa-miR-548w-5p	MIMAT0015060	na	na
1286_	hsa-mir-548x	MI0014244	hsa-miR-548x-5p	MIMAT0022733	hsa-miR-548x-3p	MIMAT0015081
1287_	hsa-mir-548x-2	MI0016833	na	na	hsa-miR-548x-3p	MIMAT0015081
1288_	hsa-mir-548y	MI0016595	hsa-miR-548y-5p	MIMAT0018354	na	na
1289_	hsa-mir-548z	MI0016688	na	na	hsa-miR-548z-3p	MIMAT0018446
1290_	hsa-mir-549a	MI0003679	na	na	hsa-miR-549a-3p	MIMAT0003333
1291_	hsa-mir-550a-1	MI0003600	hsa-miR-550a-3-5p	MIMAT0020925	hsa-miR-550a-3p	MIMAT0003257
1292_	hsa-mir-550a-2	MI0003601	hsa-miR-550a-3-5p	MIMAT0020925	hsa-miR-550a-3p	MIMAT0003257
1293_	hsa-mir-550a-3	MI0003762	hsa-miR-550a-3-5p	MIMAT0020925	hsa-miR-550a-3p	MIMAT0003257
1294_	hsa-mir-550b-1	MI0016686	hsa-miR-550b-2-5p	MIMAT0022737	hsa-miR-550b-3p	MIMAT0018445
1295_	hsa-mir-550b-2	MI0016687	hsa-miR-550b-2-5p	MIMAT0022737	hsa-miR-550b-3p	MIMAT0018445
1296_	hsa-mir-551a	MI0003556	na	na	hsa-miR-551a-3p	MIMAT0003214
1297_	hsa-mir-551b	MI0003575	hsa-miR-551b-5p	MIMAT0004794	hsa-miR-551b-3p	MIMAT0003233
1298_	hsa-mir-552	MI0003557	hsa-miR-552-5p	MIMAT0026615	hsa-miR-552-3p	MIMAT0003215
1299_	hsa-mir-553	MI0003558	hsa-miR-553-5p	MIMAT0003216	na	na
1300_	hsa-mir-554	MI0003559	hsa-miR-554-5p	MIMAT0003217	na	na
1301_	hsa-mir-555	MI0003561	na	na	hsa-miR-555-3p	MIMAT0003219
1302_	hsa-mir-556	MI0003562	hsa-miR-556-5p	MIMAT0003220	hsa-miR-556-3p	MIMAT0004793
1303_	hsa-mir-557	MI0003563	na	na	hsa-miR-557-3p	MIMAT0003221
1304_	hsa-mir-5571	MI0019115	hsa-miR-5571-5p	MIMAT0022257	hsa-miR-5571-3p	MIMAT0022258
1305_	hsa-mir-5572	MI0019117	na	na	hsa-miR-5572-3p	MIMAT0022260
1306_	hsa-mir-5579	MI0019133	hsa-miR-5579-5p	MIMAT0022269	hsa-miR-5579-3p	MIMAT0022270
1307_	hsa-mir-558	MI0003564	na	na	hsa-miR-558-3p	MIMAT0003222
1308_	hsa-mir-5580	MI0019135	hsa-miR-5580-5p	MIMAT0022273	hsa-miR-5580-3p	MIMAT0022274

1309_	hsa-mir-5581	MI0019136	hsa-miR-5581-5p	MIMAT0022275	hsa-miR-5581-3p	MIMAT0022276
1310_	hsa-mir-5582	MI0019138	hsa-miR-5582-5p	MIMAT0022279	hsa-miR-5582-3p	MIMAT0022280
1311_	hsa-mir-5583-1	MI0019139	hsa-miR-5583-5p	MIMAT0022281	hsa-miR-5583-3p	MIMAT0022282
1312_	hsa-mir-5583-2	MI0019140	hsa-miR-5583-5p	MIMAT0022281	hsa-miR-5583-3p	MIMAT0022282
1313_	hsa-mir-5584	MI0019141	hsa-miR-5584-5p	MIMAT0022283	hsa-miR-5584-3p	MIMAT0022284
1314_	hsa-mir-5585	MI0019142	hsa-miR-5585-5p	MIMAT0022285	hsa-miR-5585-3p	MIMAT0022286
1315_	hsa-mir-5586	MI0019143	hsa-miR-5586-5p	MIMAT0022287	hsa-miR-5586-3p	MIMAT0022288
1316_	hsa-mir-5587	MI0019144	hsa-miR-5587-5p	MIMAT0022289	hsa-miR-5587-3p	MIMAT0022290
1317_	hsa-mir-5588	MI0019147	hsa-miR-5588-5p	MIMAT0022295	hsa-miR-5588-3p	MIMAT0022296
1318_	hsa-mir-5589	MI0019148	hsa-miR-5589-5p	MIMAT0022297	hsa-miR-5589-3p	MIMAT0022298
1319_	hsa-mir-559	MI0003565	hsa-miR-559-5p	MIMAT0003223	na	na
1320_	hsa-mir-5590	MI0019150	hsa-miR-5590-5p	MIMAT0022299	hsa-miR-5590-3p	MIMAT0022300
1321_	hsa-mir-5591	MI0019151	hsa-miR-5591-5p	MIMAT0022301	hsa-miR-5591-3p	MIMAT0022302
1322_	hsa-mir-561	MI0003567	hsa-miR-561-5p	MIMAT0022706	hsa-miR-561-3p	MIMAT0003225
1323_	hsa-mir-562	MI0003568	na	na	hsa-miR-562-3p	MIMAT0003226
1324_	hsa-mir-563	MI0003569	na	na	hsa-miR-563-3p	MIMAT0003227
1325_	hsa-mir-564	MI0003570	hsa-miR-564-5p	MIMAT0003228	na	na
1326_	hsa-mir-566	MI0003572	hsa-miR-566-5p	MIMAT0003230	na	na
1327_	hsa-mir-567	MI0003573	hsa-miR-567-5p	MIMAT0003231	na	na
1328_	hsa-mir-568	MI0003574	hsa-miR-568-5p	MIMAT0003232	na	na
1329_	hsa-mir-5680	MI0019280	na	na	hsa-miR-5680-3p	MIMAT0022468
1330_	hsa-mir-5681a	MI0019281	na	na	hsa-miR-5681a-3p	MIMAT0022469
1331_	hsa-mir-5681b	MI0019293	hsa-miR-5681b-5p	MIMAT0022480	na	na
1332_	hsa-mir-5682	MI0019282	na	na	hsa-miR-5682-3p	MIMAT0022470
1333_	hsa-mir-5683	MI0019284	hsa-miR-5683-5p	MIMAT0022472	na	na
1334_	hsa-mir-5684	MI0019285	hsa-miR-5684-5p	MIMAT0022473	na	na
1335_	hsa-mir-5685	MI0019287	hsa-miR-5685-5p	MIMAT0022475	na	na
1336_	hsa-mir-5687	MI0019291	na	na	hsa-miR-5687-3p	MIMAT0022478
1337_	hsa-mir-5688	MI0019292	na	na	hsa-miR-5688-3p	MIMAT0022479

1338_	hsa-mir-5689	MI0019294	hsa-miR-5689-5p	MIMAT0022481	na	na
1339_	hsa-mir-569	MI0003576	na	na	hsa-miR-569-3p	MIMAT0003234
1340_	hsa-mir-5690	MI0019295	hsa-miR-5690-5p	MIMAT0022482	na	na
1341_	hsa-mir-5691	MI0019296	hsa-miR-5691-5p	MIMAT0022483	na	na
1342_	hsa-mir-5692a-1	MI0019297	hsa-miR-5692a-5p	MIMAT0022484	na	na
1343_	hsa-mir-5692a-2	MI0019298	hsa-miR-5692a-5p	MIMAT0022484	na	na
1344_	hsa-mir-5692b	MI0019311	hsa-miR-5692b-5p	MIMAT0022497	na	na
1345_	hsa-mir-5692c-1	MI0019288	na	na	hsa-miR-5692c-3p	MIMAT0022476
1346_	hsa-mir-5692c-2	MI0019289	na	na	hsa-miR-5692c-3p	MIMAT0022476
1347_	hsa-mir-5693	MI0019300	na	na	hsa-miR-5693-3p	MIMAT0022486
1348_	hsa-mir-5694	MI0019301	hsa-miR-5694-5p	MIMAT0022487	na	na
1349_	hsa-mir-5695	MI0019302	na	na	hsa-miR-5695-3p	MIMAT0022488
1350_	hsa-mir-5696	MI0019303	hsa-miR-5696-5p	MIMAT0022489	na	na
1351_	hsa-mir-5697	MI0019304	hsa-miR-5697-5p	MIMAT0022490	na	na
1352_	hsa-mir-5698	MI0019305	hsa-miR-5698-5p	MIMAT0022491	na	na
1353_	hsa-mir-5699	MI0019306	hsa-miR-5699-5p	MIMAT0027103	hsa-miR-5699-3p	MIMAT0022492
1354_	hsa-mir-570	MI0003577	hsa-miR-570-5p	MIMAT0022707	hsa-miR-570-3p	MIMAT0003235
1355_	hsa-mir-5700	MI0019307	hsa-miR-5700-5p	MIMAT0022493	na	na
1356_	hsa-mir-5701-1	MI0019308	hsa-miR-5701-5p	MIMAT0022494	na	na
1357_	hsa-mir-5701-2	MI0019593	hsa-miR-5701-5p	MIMAT0022494	na	na
1358_	hsa-mir-5702	MI0019309	na	na	hsa-miR-5702-3p	MIMAT0022495
1359_	hsa-mir-5703	MI0019310	na	na	hsa-miR-5703-3p	MIMAT0022496
1360_	hsa-mir-5704	MI0019312	hsa-miR-5704-5p	MIMAT0022498	na	na
1361_	hsa-mir-5705	MI0019313	na	na	hsa-miR-5705-3p	MIMAT0022499
1362_	hsa-mir-5706	MI0019314	hsa-miR-5706-5p	MIMAT0022500	na	na
1363_	hsa-mir-5707	MI0019315	hsa-miR-5707-5p	MIMAT0022501	na	na
1364_	hsa-mir-5708	MI0019316	hsa-miR-5708-5p	MIMAT0022502	na	na
1365_	hsa-mir-571	MI0003578	na	na	hsa-miR-571-3p	MIMAT0003236
1366_	hsa-mir-572	MI0003579	na	na	hsa-miR-572-3p	MIMAT0003237

1367_	hsa-mir-573	MI0003580	hsa-miR-573-5p	MIMAT0003238	na	na
1368_	hsa-mir-5739	MI0019412	na	na	hsa-miR-5739-3p	MIMAT0023116
1369_	hsa-mir-574	MI0003581	hsa-miR-574-5p	MIMAT0004795	hsa-miR-574-3p	MIMAT0003239
1370_	hsa-mir-575	MI0003582	na	na	hsa-miR-575-3p	MIMAT0003240
1371_	hsa-mir-576	MI0003583	hsa-miR-576-5p	MIMAT0003241	hsa-miR-576-3p	MIMAT0004796
1372_	hsa-mir-577	MI0003584	hsa-miR-577-5p	MIMAT0003242	na	na
1373_	hsa-mir-578	MI0003585	na	na	hsa-miR-578-3p	MIMAT0003243
1374_	hsa-mir-5787	MI0019797	hsa-miR-5787-5p	MIMAT0023252	na	na
1375_	hsa-mir-579	MI0003586	hsa-miR-579-5p	MIMAT0026616	hsa-miR-579-3p	MIMAT0003244
1376_	hsa-mir-580	MI0003587	hsa-miR-580-5p	MIMAT0026617	hsa-miR-580-3p	MIMAT0003245
1377_	hsa-mir-581	MI0003588	hsa-miR-581-5p	MIMAT0003246	na	na
1378_	hsa-mir-582	MI0003589	hsa-miR-582-5p	MIMAT0003247	hsa-miR-582-3p	MIMAT0004797
1379_	hsa-mir-583	MI0003590	hsa-miR-583-5p	MIMAT0003248	na	na
1380_	hsa-mir-584	MI0003591	hsa-miR-584-5p	MIMAT0003249	hsa-miR-584-3p	MIMAT0022708
1381_	hsa-mir-585	MI0003592	hsa-miR-585-5p	MIMAT0026618	hsa-miR-585-3p	MIMAT0003250
1382_	hsa-mir-586	MI0003594	hsa-miR-586-5p	MIMAT0003252	na	na
1383_	hsa-mir-587	MI0003595	hsa-miR-587-5p	MIMAT0003253	na	na
1384_	hsa-mir-588	MI0003597	hsa-miR-588-5p	MIMAT0003255	na	na
1385_	hsa-mir-589	MI0003599	hsa-miR-589-5p	MIMAT0004799	hsa-miR-589-3p	MIMAT0003256
1386_	hsa-mir-590	MI0003602	hsa-miR-590-5p	MIMAT0003258	hsa-miR-590-3p	MIMAT0004801
1387_	hsa-mir-591	MI0003603	hsa-miR-591-5p	MIMAT0003259	na	na
1388_	hsa-mir-592	MI0003604	hsa-miR-592-5p	MIMAT0003260	na	na
1389_	hsa-mir-593	MI0003605	hsa-miR-593-5p	MIMAT0003261	hsa-miR-593-3p	MIMAT0004802
1390_	hsa-mir-595	MI0003607	na	na	hsa-miR-595-3p	MIMAT0003263
1391_	hsa-mir-596	MI0003608	hsa-miR-596-5p	MIMAT0003264	na	na
1392_	hsa-mir-597	MI0003609	hsa-miR-597-5p	MIMAT0003265	hsa-miR-597-3p	MIMAT0026619
1393_	hsa-mir-598	MI0003610	hsa-miR-598-5p	MIMAT0026620	hsa-miR-1322-3p	MIMAT0005953
1394_	hsa-mir-599	MI0003611	na	na	hsa-miR-599-3p	MIMAT0003267
1395_	hsa-mir-600	MI0003613	na	na	hsa-miR-600-3p	MIMAT0003268

1396_	hsa-mir-601	MI0003614	hsa-miR-601-5p	MIMAT0003269	na	na
1397_	hsa-mir-602	MI0003615	hsa-miR-602-5p	MIMAT0003270	na	na
1398_	hsa-mir-603	MI0003616	na	na	hsa-miR-603-3p	MIMAT0003271
1399_	hsa-mir-604	MI0003617	na	na	hsa-miR-604-3p	MIMAT0003272
1400_	hsa-mir-605	MI0003618	hsa-miR-605-5p	MIMAT0003273	hsa-miR-605-3p	MIMAT0026621
1401_	hsa-mir-606	MI0003619	na	na	hsa-miR-606-3p	MIMAT0003274
1402_	hsa-mir-6068	MI0020345	hsa-miR-6068-5p	MIMAT0023693	na	na
1403_	hsa-mir-6069	MI0020346	hsa-miR-6069-5p	MIMAT0023694	na	na
1404_	hsa-mir-607	MI0003620	na	na	hsa-miR-607-3p	MIMAT0003275
1405_	hsa-mir-6070	MI0020347	hsa-miR-6070-5p	MIMAT0023695	na	na
1406_	hsa-mir-6071	MI0020348	hsa-miR-6071-5p	MIMAT0023696	na	na
1407_	hsa-mir-6072	MI0020349	na	na	hsa-miR-6072-3p	MIMAT0023697
1408_	hsa-mir-6073	MI0020350	na	na	hsa-miR-6073-3p	MIMAT0023698
1409_	hsa-mir-6074	MI0020351	na	na	hsa-miR-6074-3p	MIMAT0023699
1410_	hsa-mir-6075	MI0020352	na	na	hsa-miR-6075-3p	MIMAT0023700
1411_	hsa-mir-6076	MI0020353	hsa-miR-6076-5p	MIMAT0023701	na	na
1412_	hsa-mir-6077-1	MI0020354	hsa-miR-6077-5p	MIMAT0023702	na	na
1413_	hsa-mir-6077-2	MI0023562	hsa-miR-6077-5p	MIMAT0023702	na	na
1414_	hsa-mir-6078	MI0020355	hsa-miR-6078-5p	MIMAT0023703	na	na
1415_	hsa-mir-6079	MI0020356	na	na	hsa-miR-6079-3p	MIMAT0023704
1416_	hsa-mir-608	MI0003621	hsa-miR-608-5p	MIMAT0003276	na	na
1417_	hsa-mir-6080	MI0020357	na	na	hsa-miR-6080-3p	MIMAT0023705
1418_	hsa-mir-6081	MI0020358	na	na	hsa-miR-6081-3p	MIMAT0023706
1419_	hsa-mir-6082	MI0020359	hsa-miR-6082-5p	MIMAT0023707	na	na
1420_	hsa-mir-6083	MI0020360	na	na	hsa-miR-6083-3p	MIMAT0023708
1421_	hsa-mir-6084	MI0020361	na	na	hsa-miR-6084-3p	MIMAT0023709
1422_	hsa-mir-6085	MI0020362	na	na	hsa-miR-6085-3p	MIMAT0023710
1423_	hsa-mir-6086	MI0020363	hsa-miR-6086-5p	MIMAT0023711	na	na
1424_	hsa-mir-6087	MI0020364	hsa-miR-6087-5p	MIMAT0023712	na	na

1425_	hsa-mir-6088	MI0020365	hsa-miR-6088-5p	MIMAT0023713	na	na
1426_	hsa-mir-6089-1	MI0020366	na	na	hsa-miR-6089-3p	MIMAT0023714
1427_	hsa-mir-6089-2	MI0023563	na	na	hsa-miR-6089-3p	MIMAT0023714
1428_	hsa-mir-609	MI0003622	hsa-miR-609-5p	MIMAT0003277	na	na
1429_	hsa-mir-6090	MI0020367	na	na	hsa-miR-6090-3p	MIMAT0023715
1430_	hsa-mir-610	MI0003623	hsa-miR-610-5p	MIMAT0003278	na	na
1431_	hsa-mir-611	MI0003624	na	na	hsa-miR-611-3p	MIMAT0003279
1432_	hsa-mir-612	MI0003625	hsa-miR-612-5p	MIMAT0003280	na	na
1433_	hsa-mir-6124	MI0021258	hsa-miR-6124-5p	MIMAT0024597	na	na
1434_	hsa-mir-6125	MI0021259	na	na	hsa-miR-6125-3p	MIMAT0024598
1435_	hsa-mir-6126	MI0021260	hsa-miR-6126-5p	MIMAT0024599	na	na
1436_	hsa-mir-6127	MI0021271	hsa-miR-6127-5p	MIMAT0024610	na	na
1437_	hsa-mir-6128	MI0021272	na	na	hsa-miR-6128-3p	MIMAT0024611
1438_	hsa-mir-6129	MI0021274	hsa-miR-6129-5p	MIMAT0024613	na	na
1439_	hsa-mir-613	MI0003626	na	na	hsa-miR-613-3p	MIMAT0003281
1440_	hsa-mir-6130	MI0021275	na	na	hsa-miR-6130-3p	MIMAT0024614
1441_	hsa-mir-6131	MI0021276	na	na	hsa-miR-6131-3p	MIMAT0024615
1442_	hsa-mir-6132	MI0021277	hsa-miR-6132-5p	MIMAT0024616	na	na
1443_	hsa-mir-6133	MI0021278	na	na	hsa-miR-6133-3p	MIMAT0024617
1444_	hsa-mir-6134	MI0021279	na	na	hsa-miR-6134-3p	MIMAT0024618
1445_	hsa-mir-614	MI0003627	na	na	hsa-miR-614-3p	MIMAT0003282
1446_	hsa-mir-615	MI0003628	hsa-miR-615-5p	MIMAT0004804	hsa-miR-615-3p	MIMAT0003283
1447_	hsa-mir-616	MI0003629	hsa-miR-616-5p	MIMAT0003284	hsa-miR-616-3p	MIMAT0004805
1448_	hsa-mir-6165	MI0021472	hsa-miR-6165-5p	MIMAT0024782	na	na
1449_	hsa-mir-617	MI0003631	hsa-miR-617-5p	MIMAT0003286	na	na
1450_	hsa-mir-618	MI0003632	hsa-miR-618-5p	MIMAT0003287	na	na
1451_	hsa-mir-619	MI0003633	hsa-miR-619-5p	MIMAT0026622	hsa-miR-619-3p	MIMAT0003288
1452_	hsa-mir-620	MI0003634	na	na	hsa-miR-620-3p	MIMAT0003289
1453_	hsa-mir-621	MI0003635	na	na	hsa-miR-621-3p	MIMAT0003290

1454_	hsa-mir-622	MI0003636	na	na	hsa-miR-622-3p	MIMAT0003291
1455_	hsa-mir-623	MI0003637	hsa-miR-623-5p	MIMAT0003292	na	na
1456_	hsa-mir-624	MI0003638	hsa-miR-624-5p	MIMAT0003293	hsa-miR-624-3p	MIMAT0004807
1457_	hsa-mir-625	MI0003639	hsa-miR-625-5p	MIMAT0003294	hsa-miR-625-3p	MIMAT0004808
1458_	hsa-mir-626	MI0003640	na	na	hsa-miR-626-3p	MIMAT0003295
1459_	hsa-mir-627	MI0003641	hsa-miR-627-5p	MIMAT0003296	hsa-miR-627-3p	MIMAT0026623
1460_	hsa-mir-628	MI0003642	hsa-miR-628-5p	MIMAT0004809	hsa-miR-628-3p	MIMAT0003297
1461_	hsa-mir-629	MI0003643	hsa-miR-629-5p	MIMAT0004810	hsa-miR-629-3p	MIMAT0003298
1462_	hsa-mir-630	MI0003644	na	na	hsa-miR-630-3p	MIMAT0003299
1463_	hsa-mir-631	MI0003645	hsa-miR-631-5p	MIMAT0003300	na	na
1464_	hsa-mir-632	MI0003647	na	na	hsa-miR-632-3p	MIMAT0003302
1465_	hsa-mir-633	MI0003648	na	na	hsa-miR-633-3p	MIMAT0003303
1466_	hsa-mir-634	MI0003649	na	na	hsa-miR-634-3p	MIMAT0003304
1467_	hsa-mir-635	MI0003650	hsa-miR-635-5p	MIMAT0003305	na	na
1468_	hsa-mir-636	MI0003651	na	na	hsa-miR-636-3p	MIMAT0003306
1469_	hsa-mir-637	MI0003652	na	na	hsa-miR-637-3p	MIMAT0003307
1470_	hsa-mir-638	MI0003653	hsa-miR-638-5p	MIMAT0003308	na	na
1471_	hsa-mir-639	MI0003654	na	na	hsa-miR-639-3p	MIMAT0003309
1472_	hsa-mir-640	MI0003655	na	na	hsa-miR-640-3p	MIMAT0003310
1473_	hsa-mir-641	MI0003656	hsa-miR-641-5p	MIMAT0003311	na	na
1474_	hsa-mir-642a	MI0003657	hsa-miR-642a-5p	MIMAT0003312	hsa-miR-642a-3p	MIMAT0020924
1475_	hsa-mir-642b	MI0016685	hsa-miR-642b-5p	MIMAT0022736	hsa-miR-642b-3p	MIMAT0018444
1476_	hsa-mir-643	MI0003658	na	na	hsa-miR-643-3p	MIMAT0003313
1477_	hsa-mir-644a	MI0003659	na	na	hsa-miR-644a-3p	MIMAT0003314
1478_	hsa-mir-645	MI0003660	na	na	hsa-miR-645-3p	MIMAT0003315
1479_	hsa-mir-646	MI0003661	na	na	hsa-miR-646-3p	MIMAT0003316
1480_	hsa-mir-647	MI0003662	hsa-miR-647-5p	MIMAT0003317	na	na
1481_	hsa-mir-648	MI0003663	hsa-miR-648-5p	MIMAT0003318	na	na
1482_	hsa-mir-649	MI0003664	na	na	hsa-miR-649-3p	MIMAT0003319

1483_	hsa-mir-6499	MI0022209	hsa-miR-6499-5p	MIMAT0025450	hsa-miR-6499-3p	MIMAT0025451
1484_	hsa-mir-650	MI0003665	hsa-miR-650-5p	MIMAT0003320	na	na
1485_	hsa-mir-6500	MI0022211	hsa-miR-6500-5p	MIMAT0025454	hsa-miR-6500-3p	MIMAT0025455
1486_	hsa-mir-6501	MI0022213	hsa-miR-6501-5p	MIMAT0025458	hsa-miR-6501-3p	MIMAT0025459
1487_	hsa-mir-6502	MI0022214	hsa-miR-6502-5p	MIMAT0025460	hsa-miR-6502-3p	MIMAT0025461
1488_	hsa-mir-6503	MI0022215	hsa-miR-6503-5p	MIMAT0025462	hsa-miR-6503-3p	MIMAT0025463
1489_	hsa-mir-6504	MI0022216	hsa-miR-6504-5p	MIMAT0025464	hsa-miR-6504-3p	MIMAT0025465
1490_	hsa-mir-6505	MI0022217	hsa-miR-6505-5p	MIMAT0025466	hsa-miR-6505-3p	MIMAT0025467
1491_	hsa-mir-6506	MI0022218	hsa-miR-6506-5p	MIMAT0025468	hsa-miR-6506-3p	MIMAT0025469
1492_	hsa-mir-6507	MI0022219	hsa-miR-6507-5p	MIMAT0025470	hsa-miR-6507-3p	MIMAT0025471
1493_	hsa-mir-6508	MI0022220	hsa-miR-6508-5p	MIMAT0025472	hsa-miR-6508-3p	MIMAT0025473
1494_	hsa-mir-6509	MI0022221	hsa-miR-6509-5p	MIMAT0025474	hsa-miR-6509-3p	MIMAT0025475
1495_	hsa-mir-651	MI0003666	hsa-miR-651-5p	MIMAT0003321	hsa-miR-651-3p	MIMAT0026624
1496_	hsa-mir-6510	MI0022222	hsa-miR-6510-5p	MIMAT0025476	hsa-miR-6510-3p	MIMAT0025477
1497_	hsa-mir-6511a-1	MI0022223	hsa-miR-6511a-5p	MIMAT0025478	hsa-miR-6511a-3p	MIMAT0025479
1498_	hsa-mir-6511a-2	MI0023564	hsa-miR-6511a-5p	MIMAT0025478	hsa-miR-6511a-3p	MIMAT0025479
1499_	hsa-mir-6511a-3	MI0023565	hsa-miR-6511a-5p	MIMAT0025478	hsa-miR-6511a-3p	MIMAT0025479
1500_	hsa-mir-6511a-4	MI0023566	hsa-miR-6511a-5p	MIMAT0025478	hsa-miR-6511a-3p	MIMAT0025479
1501_	hsa-mir-6511b-1	MI0022552	hsa-miR-6511b-5p	MIMAT0025847	hsa-miR-6511b-3p	MIMAT0025848
1502_	hsa-mir-6511b-2	MI0023431	hsa-miR-6511b-5p	MIMAT0025847	hsa-miR-6511b-3p	MIMAT0025848
1503_	hsa-mir-6512	MI0022224	hsa-miR-6512-5p	MIMAT0025480	hsa-miR-6512-3p	MIMAT0025481
1504_	hsa-mir-6513	MI0022225	hsa-miR-6513-5p	MIMAT0025482	hsa-miR-6513-3p	MIMAT0025483
1505_	hsa-mir-6514	MI0022226	hsa-miR-6514-5p	MIMAT0025484	hsa-miR-6514-3p	MIMAT0025485
1506_	hsa-mir-6515	MI0022227	hsa-miR-6515-5p	MIMAT0025486	hsa-miR-6515-3p	MIMAT0025487
1507_	hsa-mir-6516	MI0025513	hsa-miR-6516-5p	MIMAT0030417	hsa-miR-6516-3p	MIMAT0030418
1508_	hsa-mir-652	MI0003667	hsa-miR-652-5p	MIMAT0022709	hsa-miR-652-3p	MIMAT0003322
1509_	hsa-mir-653	MI0003674	hsa-miR-653-5p	MIMAT0003328	hsa-miR-653-3p	MIMAT0026625
1510_	hsa-mir-654	MI0003676	hsa-miR-654-5p	MIMAT0003330	hsa-miR-654-3p	MIMAT0004814
1511_	hsa-mir-655	MI0003677	hsa-miR-655-5p	MIMAT0026626	hsa-miR-655-3p	MIMAT0003331

1512_	hsa-mir-656	MI0003678	hsa-miR-656-5p	MIMAT0026627	hsa-miR-656-3p	MIMAT0003332
1513_	hsa-mir-657	MI0003681	na	na	hsa-miR-657-3p	MIMAT0003335
1514_	hsa-mir-658	MI0003682	na	na	hsa-miR-658-3p	MIMAT0003336
1515_	hsa-mir-659	MI0003683	hsa-miR-659-5p	MIMAT0022710	hsa-miR-659-3p	MIMAT0003337
1516_	hsa-mir-660	MI0003684	hsa-miR-660-5p	MIMAT0003338	hsa-miR-660-3p	MIMAT0022711
1517_	hsa-mir-661	MI0003669	na	na	hsa-miR-661-3p	MIMAT0003324
1518_	hsa-mir-662	MI0003670	na	na	hsa-miR-662-3p	MIMAT0003325
1519_	hsa-mir-663a	MI0003672	hsa-miR-663a-5p	MIMAT0003326	na	na
1520_	hsa-mir-663b	MI0006336	na	na	hsa-miR-663b-3p	MIMAT0005867
1521_	hsa-mir-664a	MI0006442	hsa-miR-664a-5p	MIMAT0005948	hsa-miR-664a-3p	MIMAT0005949
1522_	hsa-mir-664b	MI0019134	hsa-miR-664b-5p	MIMAT0022271	hsa-miR-664b-3p	MIMAT0022272
1523_	hsa-mir-665	MI0005563	na	na	hsa-miR-665-3p	MIMAT0004952
1524_	hsa-mir-668	MI0003761	hsa-miR-668-5p	MIMAT0026636	hsa-miR-668-3p	MIMAT0003881
1525_	hsa-mir-670	MI0003933	hsa-miR-670-5p	MIMAT0010357	hsa-miR-670-3p	MIMAT0026640
1526_	hsa-mir-671	MI0003760	hsa-miR-671-5p	MIMAT0003880	hsa-miR-671-3p	MIMAT0004819
1527_	hsa-mir-6715a	MI0022548	na	na	hsa-miR-6715a-3p	MIMAT0025841
1528_	hsa-mir-6715b	MI0022549	hsa-miR-6715b-5p	MIMAT0025842	hsa-miR-6715b-3p	MIMAT0025843
1529_	hsa-mir-6716	MI0022550	hsa-miR-6716-5p	MIMAT0025844	hsa-miR-6716-3p	MIMAT0025845
1530_	hsa-mir-6717	MI0022551	hsa-miR-6717-5p	MIMAT0025846	na	na
1531_	hsa-mir-6718	MI0022553	hsa-miR-6718-5p	MIMAT0025849	na	na
1532_	hsa-mir-6719	MI0022554	na	na	hsa-miR-6719-3p	MIMAT0025850
1533_	hsa-mir-6720	MI0022555	hsa-miR-6720-5p	MIMAT0027345	hsa-miR-6720-3p	MIMAT0025851
1534_	hsa-mir-6721	MI0022556	hsa-miR-6721-5p	MIMAT0025852	na	na
1535_	hsa-mir-6722	MI0022557	hsa-miR-6722-5p	MIMAT0025853	hsa-miR-6722-3p	MIMAT0025854
1536_	hsa-mir-6723	MI0022558	hsa-miR-6723-5p	MIMAT0025855	na	na
1537_	hsa-mir-6724	MI0022559	hsa-miR-6724-5p	MIMAT0025856	na	na
1538_	hsa-mir-6726	MI0022571	hsa-miR-6726-5p	MIMAT0027353	hsa-miR-6726-3p	MIMAT0027354
1539_	hsa-mir-6727	MI0022572	hsa-miR-6727-5p	MIMAT0027355	hsa-miR-6727-3p	MIMAT0027356
1540_	hsa-mir-6728	MI0022573	hsa-miR-6728-5p	MIMAT0027357	hsa-miR-6728-3p	MIMAT0027358

1541_	hsa-mir-6729	MI0022574	hsa-miR-6729-5p	MIMAT0027359	hsa-miR-6729-3p	MIMAT0027360
1542_	hsa-mir-6730	MI0022575	hsa-miR-6730-5p	MIMAT0027361	hsa-miR-6730-3p	MIMAT0027362
1543_	hsa-mir-6731	MI0022576	hsa-miR-6731-5p	MIMAT0027363	hsa-miR-6731-3p	MIMAT0027364
1544_	hsa-mir-6732	MI0022577	hsa-miR-6732-5p	MIMAT0027365	hsa-miR-6732-3p	MIMAT0027366
1545_	hsa-mir-6733	MI0022578	hsa-miR-6733-5p	MIMAT0027367	hsa-miR-6733-3p	MIMAT0027368
1546_	hsa-mir-6734	MI0022579	hsa-miR-6734-5p	MIMAT0027369	hsa-miR-6734-3p	MIMAT0027370
1547_	hsa-mir-6735	MI0022580	hsa-miR-6735-5p	MIMAT0027371	hsa-miR-6735-3p	MIMAT0027372
1548_	hsa-mir-6736	MI0022581	hsa-miR-6736-5p	MIMAT0027373	hsa-miR-6736-3p	MIMAT0027374
1549_	hsa-mir-6737	MI0022582	hsa-miR-6737-5p	MIMAT0027375	hsa-miR-6737-3p	MIMAT0027376
1550_	hsa-mir-6738	MI0022583	hsa-miR-6738-5p	MIMAT0027377	hsa-miR-6738-3p	MIMAT0027378
1551_	hsa-mir-6739	MI0022584	hsa-miR-6739-5p	MIMAT0027379	hsa-miR-6739-3p	MIMAT0027380
1552_	hsa-mir-6740	MI0022585	hsa-miR-6740-5p	MIMAT0027381	hsa-miR-6740-3p	MIMAT0027382
1553_	hsa-mir-6741	MI0022586	hsa-miR-6741-5p	MIMAT0027383	hsa-miR-6741-3p	MIMAT0027384
1554_	hsa-mir-6742	MI0022587	hsa-miR-6742-5p	MIMAT0027385	hsa-miR-6742-3p	MIMAT0027386
1555_	hsa-mir-6743	MI0022588	hsa-miR-6743-5p	MIMAT0027387	hsa-miR-6743-3p	MIMAT0027388
1556_	hsa-mir-6744	MI0022589	hsa-miR-6744-5p	MIMAT0027389	hsa-miR-6744-3p	MIMAT0027390
1557_	hsa-mir-6745	MI0022590	na	na	hsa-miR-6745-3p	MIMAT0027391
1558_	hsa-mir-6746	MI0022591	hsa-miR-6746-5p	MIMAT0027392	hsa-miR-6746-3p	MIMAT0027393
1559_	hsa-mir-6747	MI0022592	hsa-miR-6747-5p	MIMAT0027394	hsa-miR-6747-3p	MIMAT0027395
1560_	hsa-mir-6748	MI0022593	hsa-miR-6748-5p	MIMAT0027396	hsa-miR-6748-3p	MIMAT0027397
1561_	hsa-mir-6749	MI0022594	hsa-miR-6749-5p	MIMAT0027398	hsa-miR-6749-3p	MIMAT0027399
1562_	hsa-mir-675	MI0005416	hsa-miR-675-5p	MIMAT0004284	hsa-miR-675-3p	MIMAT0006790
1563_	hsa-mir-6750	MI0022595	hsa-miR-6750-5p	MIMAT0027400	hsa-miR-6750-3p	MIMAT0027401
1564_	hsa-mir-6751	MI0022596	hsa-miR-6751-5p	MIMAT0027402	hsa-miR-6751-3p	MIMAT0027403
1565_	hsa-mir-6752	MI0022597	hsa-miR-6752-5p	MIMAT0027404	hsa-miR-6752-3p	MIMAT0027405
1566_	hsa-mir-6753	MI0022598	hsa-miR-6753-5p	MIMAT0027406	hsa-miR-6753-3p	MIMAT0027407
1567_	hsa-mir-6754	MI0022599	hsa-miR-6754-5p	MIMAT0027408	hsa-miR-6754-3p	MIMAT0027409
1568_	hsa-mir-6755	MI0022600	hsa-miR-6755-5p	MIMAT0027410	hsa-miR-6755-3p	MIMAT0027411
1569_	hsa-mir-6756	MI0022601	hsa-miR-6756-5p	MIMAT0027412	hsa-miR-6756-3p	MIMAT0027413

1570_	hsa-mir-6757	MI0022602	hsa-miR-6757-5p	MIMAT0027414	hsa-miR-6757-3p	MIMAT0027415
1571_	hsa-mir-6758	MI0022603	hsa-miR-6758-5p	MIMAT0027416	hsa-miR-6758-3p	MIMAT0027417
1572_	hsa-mir-6759	MI0022604	hsa-miR-6759-5p	MIMAT0027418	hsa-miR-6759-3p	MIMAT0027419
1573_	hsa-mir-676	MI0016436	hsa-miR-676-5p	MIMAT0018203	hsa-miR-676-3p	MIMAT0018204
1574_	hsa-mir-6760	MI0022605	hsa-miR-6760-5p	MIMAT0027420	hsa-miR-6760-3p	MIMAT0027421
1575_	hsa-mir-6761	MI0022606	hsa-miR-6761-5p	MIMAT0027422	hsa-miR-6761-3p	MIMAT0027423
1576_	hsa-mir-6762	MI0022607	hsa-miR-6762-5p	MIMAT0027424	hsa-miR-6762-3p	MIMAT0027425
1577_	hsa-mir-6763	MI0022608	hsa-miR-6763-5p	MIMAT0027426	hsa-miR-6763-3p	MIMAT0027427
1578_	hsa-mir-6764	MI0022609	hsa-miR-6764-5p	MIMAT0027428	hsa-miR-6764-3p	MIMAT0027429
1579_	hsa-mir-6765	MI0022610	hsa-miR-6765-5p	MIMAT0027430	hsa-miR-6765-3p	MIMAT0027431
1580_	hsa-mir-6766	MI0022611	hsa-miR-6766-5p	MIMAT0027432	hsa-miR-6766-3p	MIMAT0027433
1581_	hsa-mir-6767	MI0022612	hsa-miR-6767-5p	MIMAT0027434	hsa-miR-6767-3p	MIMAT0027435
1582_	hsa-mir-6768	MI0022613	hsa-miR-6768-5p	MIMAT0027436	hsa-miR-6768-3p	MIMAT0027437
1583_	hsa-mir-6769a	MI0022614	hsa-miR-6769a-5p	MIMAT0027438	hsa-miR-6769a-3p	MIMAT0027439
1584_	hsa-mir-6769b	MI0022706	hsa-miR-6769b-5p	MIMAT0027620	hsa-miR-6769b-3p	MIMAT0027621
1585_	hsa-mir-6770-1	MI0022615	hsa-miR-6770-5p	MIMAT0027440	hsa-miR-6770-3p	MIMAT0027441
1586_	hsa-mir-6770-2	MI0026418	hsa-miR-6770-5p	MIMAT0027440	hsa-miR-6770-3p	MIMAT0027441
1587_	hsa-mir-6770-3	MI0026419	hsa-miR-6770-5p	MIMAT0027440	hsa-miR-6770-3p	MIMAT0027441
1588_	hsa-mir-6771	MI0022616	hsa-miR-6771-5p	MIMAT0027442	hsa-miR-6771-3p	MIMAT0027443
1589_	hsa-mir-6772	MI0022617	hsa-miR-6772-5p	MIMAT0027444	hsa-miR-6772-3p	MIMAT0027445
1590_	hsa-mir-6773	MI0022618	hsa-miR-6773-5p	MIMAT0027446	hsa-miR-6773-3p	MIMAT0027447
1591_	hsa-mir-6774	MI0022619	hsa-miR-6774-5p	MIMAT0027448	hsa-miR-6774-3p	MIMAT0027449
1592_	hsa-mir-6775	MI0022620	hsa-miR-6775-5p	MIMAT0027450	hsa-miR-6775-3p	MIMAT0027451
1593_	hsa-mir-6776	MI0022621	hsa-miR-6776-5p	MIMAT0027452	hsa-miR-6776-3p	MIMAT0027453
1594_	hsa-mir-6777	MI0022622	hsa-miR-6777-5p	MIMAT0027454	hsa-miR-6777-3p	MIMAT0027455
1595_	hsa-mir-6778	MI0022623	hsa-miR-6778-5p	MIMAT0027456	hsa-miR-6778-3p	MIMAT0027457
1596_	hsa-mir-6779	MI0022624	hsa-miR-6779-5p	MIMAT0027458	hsa-miR-6779-3p	MIMAT0027459
1597_	hsa-mir-6780a	MI0022625	hsa-miR-6780a-5p	MIMAT0027460	hsa-miR-6780a-3p	MIMAT0027461
1598_	hsa-mir-6780b	MI0022681	hsa-miR-6780b-5p	MIMAT0027572	hsa-miR-6780b-3p	MIMAT0027573

1599_	hsa-mir-6781	MI0022626	hsa-miR-6781-5p	MIMAT0027462	hsa-miR-6781-3p	MIMAT0027463
1600_	hsa-mir-6782	MI0022627	hsa-miR-6782-5p	MIMAT0027464	hsa-miR-6782-3p	MIMAT0027465
1601_	hsa-mir-6783	MI0022628	hsa-miR-6783-5p	MIMAT0027466	hsa-miR-6783-3p	MIMAT0027467
1602_	hsa-mir-6784	MI0022629	hsa-miR-6784-5p	MIMAT0027468	hsa-miR-6784-3p	MIMAT0027469
1603_	hsa-mir-6785	MI0022630	hsa-miR-6785-5p	MIMAT0027470	hsa-miR-6785-3p	MIMAT0027471
1604_	hsa-mir-6786	MI0022631	hsa-miR-6786-5p	MIMAT0027472	hsa-miR-6786-3p	MIMAT0027473
1605_	hsa-mir-6787	MI0022632	hsa-miR-6787-5p	MIMAT0027474	hsa-miR-6787-3p	MIMAT0027475
1606_	hsa-mir-6788	MI0022633	hsa-miR-6788-5p	MIMAT0027476	hsa-miR-6788-3p	MIMAT0027477
1607_	hsa-mir-6789	MI0022634	hsa-miR-6789-5p	MIMAT0027478	hsa-miR-6789-3p	MIMAT0027479
1608_	hsa-mir-6790	MI0022635	hsa-miR-6790-5p	MIMAT0027480	hsa-miR-6790-3p	MIMAT0027481
1609_	hsa-mir-6791	MI0022636	hsa-miR-6791-5p	MIMAT0027482	hsa-miR-6791-3p	MIMAT0027483
1610_	hsa-mir-6792	MI0022637	hsa-miR-6792-5p	MIMAT0027484	hsa-miR-6792-3p	MIMAT0027485
1611_	hsa-mir-6793	MI0022638	hsa-miR-6793-5p	MIMAT0027486	hsa-miR-6793-3p	MIMAT0027487
1612_	hsa-mir-6794	MI0022639	hsa-miR-6794-5p	MIMAT0027488	hsa-miR-6794-3p	MIMAT0027489
1613_	hsa-mir-6795	MI0022640	hsa-miR-6795-5p	MIMAT0027490	hsa-miR-6795-3p	MIMAT0027491
1614_	hsa-mir-6796	MI0022641	hsa-miR-6796-5p	MIMAT0027492	hsa-miR-6796-3p	MIMAT0027493
1615_	hsa-mir-6797	MI0022642	hsa-miR-6797-5p	MIMAT0027494	hsa-miR-6797-3p	MIMAT0027495
1616_	hsa-mir-6798	MI0022643	hsa-miR-6798-5p	MIMAT0027496	hsa-miR-6798-3p	MIMAT0027497
1617_	hsa-mir-6799	MI0022644	hsa-miR-6799-5p	MIMAT0027498	hsa-miR-6799-3p	MIMAT0027499
1618_	hsa-mir-6800	MI0022645	hsa-miR-6800-5p	MIMAT0027500	hsa-miR-6800-3p	MIMAT0027501
1619_	hsa-mir-6801	MI0022646	hsa-miR-6801-5p	MIMAT0027502	hsa-miR-6801-3p	MIMAT0027503
1620_	hsa-mir-6802	MI0022647	hsa-miR-6802-5p	MIMAT0027504	hsa-miR-6802-3p	MIMAT0027505
1621_	hsa-mir-6803	MI0022648	hsa-miR-6803-5p	MIMAT0027506	hsa-miR-6803-3p	MIMAT0027507
1622_	hsa-mir-6804	MI0022649	hsa-miR-6804-5p	MIMAT0027508	hsa-miR-6804-3p	MIMAT0027509
1623_	hsa-mir-6805	MI0022650	hsa-miR-6805-5p	MIMAT0027510	hsa-miR-6805-3p	MIMAT0027511
1624_	hsa-mir-6806	MI0022651	hsa-miR-6806-5p	MIMAT0027512	hsa-miR-6806-3p	MIMAT0027513
1625_	hsa-mir-6807	MI0022652	hsa-miR-6807-5p	MIMAT0027514	hsa-miR-6807-3p	MIMAT0027515
1626_	hsa-mir-6808	MI0022653	hsa-miR-6808-5p	MIMAT0027516	hsa-miR-6808-3p	MIMAT0027517
1627_	hsa-mir-6809	MI0022654	hsa-miR-6809-5p	MIMAT0027518	hsa-miR-6809-3p	MIMAT0027519

1628_	hsa-mir-6810	MI0022655	hsa-miR-6810-5p	MIMAT0027520	hsa-miR-6810-3p	MIMAT0027521
1629_	hsa-mir-6811	MI0022656	hsa-miR-6811-5p	MIMAT0027522	hsa-miR-6811-3p	MIMAT0027523
1630_	hsa-mir-6812	MI0022657	hsa-miR-6812-5p	MIMAT0027524	hsa-miR-6812-3p	MIMAT0027525
1631_	hsa-mir-6813	MI0022658	hsa-miR-6813-5p	MIMAT0027526	hsa-miR-6813-3p	MIMAT0027527
1632_	hsa-mir-6814	MI0022659	hsa-miR-6814-5p	MIMAT0027528	hsa-miR-6814-3p	MIMAT0027529
1633_	hsa-mir-6815	MI0022660	hsa-miR-6815-5p	MIMAT0027530	hsa-miR-6815-3p	MIMAT0027531
1634_	hsa-mir-6816	MI0022661	hsa-miR-6816-5p	MIMAT0027532	hsa-miR-6816-3p	MIMAT0027533
1635_	hsa-mir-6817	MI0022662	hsa-miR-6817-5p	MIMAT0027534	hsa-miR-6817-3p	MIMAT0027535
1636_	hsa-mir-6818	MI0022663	hsa-miR-6818-5p	MIMAT0027536	hsa-miR-6818-3p	MIMAT0027537
1637_	hsa-mir-6819	MI0022664	hsa-miR-6819-5p	MIMAT0027538	hsa-miR-6819-3p	MIMAT0027539
1638_	hsa-mir-6820	MI0022665	hsa-miR-6820-5p	MIMAT0027540	hsa-miR-6820-3p	MIMAT0027541
1639_	hsa-mir-6821	MI0022666	hsa-miR-6821-5p	MIMAT0027542	hsa-miR-6821-3p	MIMAT0027543
1640_	hsa-mir-6822	MI0022667	hsa-miR-6822-5p	MIMAT0027544	hsa-miR-6822-3p	MIMAT0027545
1641_	hsa-mir-6823	MI0022668	hsa-miR-6823-5p	MIMAT0027546	hsa-miR-6823-3p	MIMAT0027547
1642_	hsa-mir-6824	MI0022669	hsa-miR-6824-5p	MIMAT0027548	hsa-miR-6824-3p	MIMAT0027549
1643_	hsa-mir-6825	MI0022670	hsa-miR-6825-5p	MIMAT0027550	hsa-miR-6825-3p	MIMAT0027551
1644_	hsa-mir-6826	MI0022671	hsa-miR-6826-5p	MIMAT0027552	hsa-miR-6826-3p	MIMAT0027553
1645_	hsa-mir-6827	MI0022672	hsa-miR-6827-5p	MIMAT0027554	hsa-miR-6827-3p	MIMAT0027555
1646_	hsa-mir-6828	MI0022673	hsa-miR-6828-5p	MIMAT0027556	hsa-miR-6828-3p	MIMAT0027557
1647_	hsa-mir-6829	MI0022674	hsa-miR-6829-5p	MIMAT0027558	hsa-miR-6829-3p	MIMAT0027559
1648_	hsa-mir-6830	MI0022675	hsa-miR-6830-5p	MIMAT0027560	hsa-miR-6830-3p	MIMAT0027561
1649_	hsa-mir-6831	MI0022676	hsa-miR-6831-5p	MIMAT0027562	hsa-miR-6831-3p	MIMAT0027563
1650_	hsa-mir-6832	MI0022677	hsa-miR-6832-5p	MIMAT0027564	hsa-miR-6832-3p	MIMAT0027565
1651_	hsa-mir-6833	MI0022678	hsa-miR-6833-5p	MIMAT0027566	hsa-miR-6833-3p	MIMAT0027567
1652_	hsa-mir-6834	MI0022679	hsa-miR-6834-5p	MIMAT0027568	hsa-miR-6834-3p	MIMAT0027569
1653_	hsa-mir-6835	MI0022680	hsa-miR-6835-5p	MIMAT0027570	hsa-miR-6835-3p	MIMAT0027571
1654_	hsa-mir-6836	MI0022682	hsa-miR-6836-5p	MIMAT0027574	hsa-miR-6836-3p	MIMAT0027575
1655_	hsa-mir-6837	MI0022683	hsa-miR-6837-5p	MIMAT0027576	hsa-miR-6837-3p	MIMAT0027577
1656_	hsa-mir-6838	MI0022684	hsa-miR-6838-5p	MIMAT0027578	hsa-miR-6838-3p	MIMAT0027579

1657_	hsa-mir-6839	MI0022685	hsa-miR-6839-5p	MIMAT0027580	hsa-miR-6839-3p	MIMAT0027581
1658_	hsa-mir-6840	MI0022686	hsa-miR-6840-5p	MIMAT0027582	hsa-miR-6840-3p	MIMAT0027583
1659_	hsa-mir-6841	MI0022687	hsa-miR-6841-5p	MIMAT0027584	hsa-miR-6841-3p	MIMAT0027585
1660_	hsa-mir-6842	MI0022688	hsa-miR-6842-5p	MIMAT0027586	hsa-miR-6842-3p	MIMAT0027587
1661_	hsa-mir-6843	MI0022689	na	na	hsa-miR-6843-3p	MIMAT0027588
1662_	hsa-mir-6844	MI0022690	na	na	hsa-miR-6844-3p	MIMAT0027589
1663_	hsa-mir-6845	MI0022691	hsa-miR-6845-5p	MIMAT0027590	hsa-miR-6845-3p	MIMAT0027591
1664_	hsa-mir-6846	MI0022692	hsa-miR-6846-5p	MIMAT0027592	hsa-miR-6846-3p	MIMAT0027593
1665_	hsa-mir-6847	MI0022693	hsa-miR-6847-5p	MIMAT0027594	hsa-miR-6847-3p	MIMAT0027595
1666_	hsa-mir-6848	MI0022694	hsa-miR-6848-5p	MIMAT0027596	hsa-miR-6848-3p	MIMAT0027597
1667_	hsa-mir-6849	MI0022695	hsa-miR-6849-5p	MIMAT0027598	hsa-miR-6849-3p	MIMAT0027599
1668_	hsa-mir-6850	MI0022696	hsa-miR-6850-5p	MIMAT0027600	hsa-miR-6850-3p	MIMAT0027601
1669_	hsa-mir-6851	MI0022697	hsa-miR-6851-5p	MIMAT0027602	hsa-miR-6851-3p	MIMAT0027603
1670_	hsa-mir-6852	MI0022698	hsa-miR-6852-5p	MIMAT0027604	hsa-miR-6852-3p	MIMAT0027605
1671_	hsa-mir-6853	MI0022699	hsa-miR-6853-5p	MIMAT0027606	hsa-miR-6853-3p	MIMAT0027607
1672_	hsa-mir-6854	MI0022700	hsa-miR-6854-5p	MIMAT0027608	hsa-miR-6854-3p	MIMAT0027609
1673_	hsa-mir-6855	MI0022701	hsa-miR-6855-5p	MIMAT0027610	hsa-miR-6855-3p	MIMAT0027611
1674_	hsa-mir-6856	MI0022702	hsa-miR-6856-5p	MIMAT0027612	hsa-miR-6856-3p	MIMAT0027613
1675_	hsa-mir-6857	MI0022703	hsa-miR-6857-5p	MIMAT0027614	hsa-miR-6857-3p	MIMAT0027615
1676_	hsa-mir-6858	MI0022704	hsa-miR-6858-5p	MIMAT0027616	hsa-miR-6858-3p	MIMAT0027617
1677_	hsa-mir-6859-1	MI0022705	hsa-miR-6859-5p	MIMAT0027618	hsa-miR-6859-3p	MIMAT0027619
1678_	hsa-mir-6859-2	MI0026420	hsa-miR-6859-5p	MIMAT0027618	hsa-miR-6859-3p	MIMAT0027619
1679_	hsa-mir-6859-3	MI0026421	hsa-miR-6859-5p	MIMAT0027618	hsa-miR-6859-3p	MIMAT0027619
1680_	hsa-mir-6860	MI0022707	na	na	hsa-miR-6860-3p	MIMAT0027622
1681_	hsa-mir-6861	MI0022708	hsa-miR-6861-5p	MIMAT0027623	hsa-miR-6861-3p	MIMAT0027624
1682_	hsa-mir-6862-1	MI0022709	hsa-miR-6862-5p	MIMAT0027625	hsa-miR-6862-3p	MIMAT0027626
1683_	hsa-mir-6862-2	MI0026415	hsa-miR-6862-5p	MIMAT0027625	hsa-miR-6862-3p	MIMAT0027626
1684_	hsa-mir-6863	MI0022710	na	na	hsa-miR-6863-3p	MIMAT0027627
1685_	hsa-mir-6864	MI0022711	hsa-miR-6864-5p	MIMAT0027628	hsa-miR-6864-3p	MIMAT0027629

1686_	hsa-mir-6865	MI0022712	hsa-miR-6865-5p	MIMAT0027630	hsa-miR-6865-3p	MIMAT0027631
1687_	hsa-mir-6866	MI0022713	hsa-miR-6866-5p	MIMAT0027632	hsa-miR-6866-3p	MIMAT0027633
1688_	hsa-mir-6867	MI0022714	hsa-miR-6867-5p	MIMAT0027634	hsa-miR-6867-3p	MIMAT0027635
1689_	hsa-mir-6868	MI0022715	hsa-miR-6868-5p	MIMAT0027636	hsa-miR-6868-3p	MIMAT0027637
1690_	hsa-mir-6869	MI0022716	hsa-miR-6869-5p	MIMAT0027638	hsa-miR-6869-3p	MIMAT0027639
1691_	hsa-mir-6870	MI0022717	hsa-miR-6870-5p	MIMAT0027640	hsa-miR-6870-3p	MIMAT0027641
1692_	hsa-mir-6871	MI0022718	hsa-miR-6871-5p	MIMAT0027642	hsa-miR-6871-3p	MIMAT0027643
1693_	hsa-mir-6872	MI0022719	hsa-miR-6872-5p	MIMAT0027644	hsa-miR-6872-3p	MIMAT0027645
1694_	hsa-mir-6873	MI0022720	hsa-miR-6873-5p	MIMAT0027646	hsa-miR-6873-3p	MIMAT0027647
1695_	hsa-mir-6874	MI0022721	hsa-miR-6874-5p	MIMAT0027648	hsa-miR-6874-3p	MIMAT0027649
1696_	hsa-mir-6875	MI0022722	hsa-miR-6875-5p	MIMAT0027650	hsa-miR-6875-3p	MIMAT0027651
1697_	hsa-mir-6876	MI0022723	hsa-miR-6876-5p	MIMAT0027652	hsa-miR-6876-3p	MIMAT0027653
1698_	hsa-mir-6877	MI0022724	hsa-miR-6877-5p	MIMAT0027654	hsa-miR-6877-3p	MIMAT0027655
1699_	hsa-mir-6878	MI0022725	hsa-miR-6878-5p	MIMAT0027656	hsa-miR-6878-3p	MIMAT0027657
1700_	hsa-mir-6879	MI0022726	hsa-miR-6879-5p	MIMAT0027658	hsa-miR-6879-3p	MIMAT0027659
1701_	hsa-mir-6880	MI0022727	hsa-miR-6880-5p	MIMAT0027660	hsa-miR-6880-3p	MIMAT0027661
1702_	hsa-mir-6881	MI0022728	hsa-miR-6881-5p	MIMAT0027662	hsa-miR-6881-3p	MIMAT0027663
1703_	hsa-mir-6882	MI0022729	hsa-miR-6882-5p	MIMAT0027664	hsa-miR-6882-3p	MIMAT0027665
1704_	hsa-mir-6883	MI0022730	hsa-miR-6883-5p	MIMAT0027666	hsa-miR-6883-3p	MIMAT0027667
1705_	hsa-mir-6884	MI0022731	hsa-miR-6884-5p	MIMAT0027668	hsa-miR-6884-3p	MIMAT0027669
1706_	hsa-mir-6885	MI0022732	hsa-miR-6885-5p	MIMAT0027670	hsa-miR-6885-3p	MIMAT0027671
1707_	hsa-mir-6886	MI0022733	hsa-miR-6886-5p	MIMAT0027672	hsa-miR-6886-3p	MIMAT0027673
1708_	hsa-mir-6887	MI0022734	hsa-miR-6887-5p	MIMAT0027674	hsa-miR-6887-3p	MIMAT0027675
1709_	hsa-mir-6888	MI0022735	hsa-miR-6888-5p	MIMAT0027676	hsa-miR-6888-3p	MIMAT0027677
1710_	hsa-mir-6889	MI0022736	hsa-miR-6889-5p	MIMAT0027678	hsa-miR-6889-3p	MIMAT0027679
1711_	hsa-mir-6890	MI0022737	hsa-miR-6890-5p	MIMAT0027680	hsa-miR-6890-3p	MIMAT0027681
1712_	hsa-mir-6891	MI0022738	hsa-miR-6891-5p	MIMAT0027682	hsa-miR-6891-3p	MIMAT0027683
1713_	hsa-mir-6892	MI0022739	hsa-miR-6892-5p	MIMAT0027684	hsa-miR-6892-3p	MIMAT0027685
1714_	hsa-mir-6893	MI0022740	hsa-miR-6893-5p	MIMAT0027686	hsa-miR-6893-3p	MIMAT0027687

1715_	hsa-mir-6894	MI0022741	hsa-miR-6894-5p	MIMAT0027688	hsa-miR-6894-3p	MIMAT0027689
1716_	hsa-mir-6895	MI0022742	hsa-miR-6895-5p	MIMAT0027690	hsa-miR-6895-3p	MIMAT0027691
1717_	hsa-mir-7-1	MI0000263	hsa-miR-7-5p	MIMAT0000252	hsa-miR-7-1-3p	MIMAT0004553
1718_	hsa-mir-7-2	MI0000264	hsa-miR-7-5p	MIMAT0000252	hsa-miR-7-2-3p	MIMAT0004554
1719_	hsa-mir-7-3	MI0000265	hsa-miR-7-5p	MIMAT0000252	na	na
1720_	hsa-mir-708	MI0005543	hsa-miR-708-5p	MIMAT0004926	hsa-miR-708-3p	MIMAT0004927
1721_	hsa-mir-7106	MI0022957	hsa-miR-7106-5p	MIMAT0028109	hsa-miR-7106-3p	MIMAT0028110
1722_	hsa-mir-7107	MI0022958	hsa-miR-7107-5p	MIMAT0028111	hsa-miR-7107-3p	MIMAT0028112
1723_	hsa-mir-7108	MI0022959	hsa-miR-7108-5p	MIMAT0028113	hsa-miR-7108-3p	MIMAT0028114
1724_	hsa-mir-7109	MI0022960	hsa-miR-7109-5p	MIMAT0028115	hsa-miR-7109-3p	MIMAT0028116
1725_	hsa-mir-711	MI0012488	na	na	hsa-miR-711-3p	MIMAT0012734
1726_	hsa-mir-7110	MI0022961	hsa-miR-7110-5p	MIMAT0028117	hsa-miR-7110-3p	MIMAT0028118
1727_	hsa-mir-7111	MI0022962	hsa-miR-7111-5p	MIMAT0028119	hsa-miR-7111-3p	MIMAT0028120
1728_	hsa-mir-7112-1	MI0022963	hsa-miR-7112-5p	MIMAT0028121	hsa-miR-7112-3p	MIMAT0028122
1729_	hsa-mir-7112-2	MI0026414	hsa-miR-7112-5p	MIMAT0028121	hsa-miR-7112-3p	MIMAT0028122
1730_	hsa-mir-7113	MI0022964	hsa-miR-7113-5p	MIMAT0028123	hsa-miR-7113-3p	MIMAT0028124
1731_	hsa-mir-7114	MI0022965	hsa-miR-7114-5p	MIMAT0028125	hsa-miR-7114-3p	MIMAT0028126
1732_	hsa-mir-7150	MI0023610	hsa-miR-7150-5p	MIMAT0028211	na	na
1733_	hsa-mir-7151	MI0023611	hsa-miR-7151-5p	MIMAT0028212	hsa-miR-7151-3p	MIMAT0028213
1734_	hsa-mir-7152	MI0023612	hsa-miR-7152-5p	MIMAT0028214	hsa-miR-7152-3p	MIMAT0028215
1735_	hsa-mir-7153	MI0023613	hsa-miR-7153-5p	MIMAT0028216	hsa-miR-7153-3p	MIMAT0028217
1736_	hsa-mir-7154	MI0023614	hsa-miR-7154-5p	MIMAT0028218	hsa-miR-7154-3p	MIMAT0028219
1737_	hsa-mir-7155	MI0023615	hsa-miR-7155-5p	MIMAT0028220	hsa-miR-7155-3p	MIMAT0028221
1738_	hsa-mir-7156	MI0023616	hsa-miR-7156-5p	MIMAT0028222	hsa-miR-7156-3p	MIMAT0028223
1739_	hsa-mir-7157	MI0023617	hsa-miR-7157-5p	MIMAT0028224	hsa-miR-7157-3p	MIMAT0028225
1740_	hsa-mir-7158	MI0023618	hsa-miR-7158-5p	MIMAT0028226	hsa-miR-7158-3p	MIMAT0028227
1741_	hsa-mir-7159	MI0023620	hsa-miR-7159-5p	MIMAT0028228	hsa-miR-7159-3p	MIMAT0028229
1742_	hsa-mir-7160	MI0023621	hsa-miR-7160-5p	MIMAT0028230	hsa-miR-7160-3p	MIMAT0028231
1743_	hsa-mir-7161	MI0023619	hsa-miR-7161-5p	MIMAT0028232	hsa-miR-7161-3p	MIMAT0028233

1744_	hsa-mir-7162	MI0023623	hsa-miR-7162-5p	MIMAT0028234	hsa-miR-7162-3p	MIMAT0028235
1745_	hsa-mir-718	MI0012489	na	na	hsa-miR-718-3p	MIMAT0012735
1746_	hsa-mir-744	MI0005559	hsa-miR-744-5p	MIMAT0004945	hsa-miR-744-3p	MIMAT0004946
1747_	hsa-mir-7515	MI0024354	na	na	hsa-miR-7515-3p	MIMAT0029310
1748_	hsa-mir-758	MI0003757	hsa-miR-758-5p	MIMAT0022929	hsa-miR-758-3p	MIMAT0003879
1749_	hsa-mir-759	MI0004065	hsa-miR-759-5p	MIMAT0010497	na	na
1750_	hsa-mir-760	MI0005567	na	na	hsa-miR-760-3p	MIMAT0004957
1751_	hsa-mir-761	MI0003941	hsa-miR-761-5p	MIMAT0010364	na	na
1752_	hsa-mir-762	MI0003892	na	na	hsa-miR-762-3p	MIMAT0010313
1753_	hsa-mir-764	MI0003944	hsa-miR-764-5p	MIMAT0010367	na	na
1754_	hsa-mir-7641-1	MI0024975	hsa-miR-7641-5p	MIMAT0029782	na	na
1755_	hsa-mir-7641-2	MI0024976	hsa-miR-7641-5p	MIMAT0029782	na	na
1756_	hsa-mir-765	MI0005116	na	na	hsa-miR-765-3p	MIMAT0003945
1757_	hsa-mir-766	MI0003836	hsa-miR-766-5p	MIMAT0022714	hsa-miR-766-3p	MIMAT0003888
1758_	hsa-mir-767	MI0003763	hsa-miR-767-5p	MIMAT0003882	hsa-miR-767-3p	MIMAT0003883
1759_	hsa-mir-769	MI0003834	hsa-miR-769-5p	MIMAT0003886	hsa-miR-769-3p	MIMAT0003887
1760_	hsa-mir-770	MI0005118	hsa-miR-770-5p	MIMAT0003948	na	na
1761_	hsa-mir-7702	MI0025238	hsa-miR-7702-5p	MIMAT0030017	na	na
1762_	hsa-mir-7703	MI0025239	na	na	hsa-miR-7703-3p	MIMAT0030018
1763_	hsa-mir-7704	MI0025240	hsa-miR-7704-5p	MIMAT0030019	na	na
1764_	hsa-mir-7705	MI0025241	hsa-miR-7705-5p	MIMAT0030020	na	na
1765_	hsa-mir-7706	MI0025242	na	na	hsa-miR-7706-3p	MIMAT0030021
1766_	hsa-mir-7843	MI0025510	hsa-miR-7843-5p	MIMAT0030411	hsa-miR-7843-3p	MIMAT0030412
1767_	hsa-mir-7844	MI0025514	hsa-miR-7844-5p	MIMAT0030419	na	na
1768_	hsa-mir-7845	MI0025515	hsa-miR-7845-5p	MIMAT0030420	na	na
1769_	hsa-mir-7846	MI0025516	na	na	hsa-miR-7846-3p	MIMAT0030421
1770_	hsa-mir-7847	MI0025517	na	na	hsa-miR-7847-3p	MIMAT0030422
1771_	hsa-mir-7848	MI0025518	na	na	hsa-miR-7848-3p	MIMAT0030423
1772_	hsa-mir-7849	MI0025519	na	na	hsa-miR-7849-3p	MIMAT0030424

1773_	hsa-mir-7850	MI0025520	hsa-miR-7850-5p	MIMAT0030425	na	na
1774_	hsa-mir-7851	MI0025521	na	na	hsa-miR-7851-3p	MIMAT0030426
1775_	hsa-mir-7852	MI0025522	na	na	hsa-miR-7852-3p	MIMAT0030427
1776_	hsa-mir-7853	MI0025523	hsa-miR-7853-5p	MIMAT0030428	na	na
1777_	hsa-mir-7854	MI0025524	na	na	hsa-miR-7854-3p	MIMAT0030429
1778_	hsa-mir-7855	MI0025525	hsa-miR-7855-5p	MIMAT0030430	na	na
1779_	hsa-mir-7856	MI0025526	hsa-miR-7856-5p	MIMAT0030431	na	na
1780_	hsa-mir-7973-1	MI0025748	hsa-miR-7973-5p	MIMAT0031176	na	na
1781_	hsa-mir-7973-2	MI0025749	hsa-miR-7973-5p	MIMAT0031176	na	na
1782_	hsa-mir-7974	MI0025750	na	na	hsa-miR-7974-3p	MIMAT0031177
1783_	hsa-mir-7975	MI0025751	na	na	hsa-miR-7975-3p	MIMAT0031178
1784_	hsa-mir-7976	MI0025752	hsa-miR-7976-5p	MIMAT0031179	na	na
1785_	hsa-mir-7977	MI0025753	hsa-miR-7977-5p	MIMAT0031180	na	na
1786_	hsa-mir-7978	MI0025754	hsa-miR-7978-5p	MIMAT0031181	na	na
1787_	hsa-mir-802	MI0003906	hsa-miR-802-5p	MIMAT0004185	na	na
1788_	hsa-mir-8052	MI0025888	hsa-miR-8052-5p	MIMAT0030979	na	na
1789_	hsa-mir-8053	MI0025889	hsa-miR-8053-5p	MIMAT0030980	na	na
1790_	hsa-mir-8054	MI0025890	hsa-miR-8054-5p	MIMAT0030981	na	na
1791_	hsa-mir-8055	MI0025891	na	na	hsa-miR-8055-3p	MIMAT0030982
1792_	hsa-mir-8056	MI0025892	na	na	hsa-miR-8056-3p	MIMAT0030983
1793_	hsa-mir-8057	MI0025893	hsa-miR-8057-5p	MIMAT0030984	na	na
1794_	hsa-mir-8058	MI0025894	na	na	hsa-miR-8058-3p	MIMAT0030985
1795_	hsa-mir-8059	MI0025895	hsa-miR-8059-5p	MIMAT0030986	na	na
1796_	hsa-mir-8060	MI0025896	na	na	hsa-miR-8060-3p	MIMAT0030987
1797_	hsa-mir-8061	MI0025897	hsa-miR-8061-5p	MIMAT0030988	na	na
1798_	hsa-mir-8062	MI0025898	hsa-miR-8062-5p	MIMAT0030989	na	na
1799_	hsa-mir-8063	MI0025899	na	na	hsa-miR-8063-3p	MIMAT0030990
1800_	hsa-mir-8064	MI0025900	hsa-miR-8064-5p	MIMAT0030991	na	na
1801_	hsa-mir-8065	MI0025901	na	na	hsa-miR-8065-3p	MIMAT0030992

1802_	hsa-mir-8066	MI0025902	na	na	hsa-miR-8066-3p	MIMAT0030993
1803_	hsa-mir-8067	MI0025903	hsa-miR-8067-5p	MIMAT0030994	na	na
1804_	hsa-mir-8068	MI0025904	hsa-miR-8068-5p	MIMAT0030995	na	na
1805_	hsa-mir-8069	MI0025905	na	na	hsa-miR-8069-3p	MIMAT0030996
1806_	hsa-mir-8070	MI0025906	hsa-miR-8070-5p	MIMAT0030997	na	na
1807_	hsa-mir-8071-1	MI0025907	na	na	hsa-miR-8071-3p	MIMAT0030998
1808_	hsa-mir-8071-2	MI0026417	na	na	hsa-miR-8071-3p	MIMAT0030998
1809_	hsa-mir-8072	MI0025908	hsa-miR-8072-5p	MIMAT0030999	na	na
1810_	hsa-mir-8073	MI0025909	hsa-miR-8073-5p	MIMAT0031000	na	na
1811_	hsa-mir-8074	MI0025910	na	na	hsa-miR-8074-3p	MIMAT0031001
1812_	hsa-mir-8075	MI0025911	na	na	hsa-miR-8075-3p	MIMAT0031002
1813_	hsa-mir-8076	MI0025912	na	na	hsa-miR-8076-3p	MIMAT0031003
1814_	hsa-mir-8077	MI0025913	na	na	hsa-miR-8077-3p	MIMAT0031004
1815_	hsa-mir-8078	MI0025914	na	na	hsa-miR-8078-3p	MIMAT0031005
1816_	hsa-mir-8079	MI0025915	hsa-miR-8079-5p	MIMAT0031006	na	na
1817_	hsa-mir-8080	MI0025916	hsa-miR-8080-5p	MIMAT0031007	na	na
1818_	hsa-mir-8081	MI0025917	na	na	hsa-miR-8081-3p	MIMAT0031008
1819_	hsa-mir-8082	MI0025918	hsa-miR-8082-5p	MIMAT0031009	na	na
1820_	hsa-mir-8083	MI0025919	hsa-miR-8083-5p	MIMAT0031010	na	na
1821_	hsa-mir-8084	MI0025920	hsa-miR-8084-5p	MIMAT0031011	na	na
1822_	hsa-mir-8085	MI0025921	hsa-miR-8085-5p	MIMAT0031012	na	na
1823_	hsa-mir-8086	MI0025922	hsa-miR-8086-5p	MIMAT0031013	na	na
1824_	hsa-mir-8087	MI0025923	hsa-miR-8087-5p	MIMAT0031014	na	na
1825_	hsa-mir-8088	MI0025924	hsa-miR-8088-5p	MIMAT0031015	na	na
1826_	hsa-mir-8089	MI0025925	na	na	hsa-miR-8089-3p	MIMAT0031016
1827_	hsa-mir-873	MI0005564	hsa-miR-873-5p	MIMAT0004953	hsa-miR-873-3p	MIMAT0022717
1828_	hsa-mir-874	MI0005532	hsa-miR-874-5p	MIMAT0026718	hsa-miR-874-3p	MIMAT0004911
1829_	hsa-mir-875	MI0005541	hsa-miR-875-5p	MIMAT0004922	hsa-miR-875-3p	MIMAT0004923
1830_	hsa-mir-876	MI0005542	hsa-miR-876-5p	MIMAT0004924	hsa-miR-876-3p	MIMAT0004925

1831_	hsa-mir-877	MI0005561	hsa-miR-877-5p	MIMAT0004949	hsa-miR-877-3p	MIMAT0004950
1832_	hsa-mir-885	MI0005560	hsa-miR-885-5p	MIMAT0004947	hsa-miR-885-3p	MIMAT0004948
1833_	hsa-mir-887	MI0005562	hsa-miR-887-5p	MIMAT0026720	hsa-miR-887-3p	MIMAT0004951
1834_	hsa-mir-888	MI0005537	hsa-miR-888-5p	MIMAT0004916	hsa-miR-888-3p	MIMAT0004917
1835_	hsa-mir-889	MI0005540	hsa-miR-889-5p	MIMAT0026719	hsa-miR-889-3p	MIMAT0004921
1836_	hsa-mir-890	MI0005533	hsa-miR-890-5p	MIMAT0004912	na	na
1837_	hsa-mir-891a	MI0005524	hsa-miR-891a-5p	MIMAT0004902	hsa-miR-891a-3p	MIMAT0026717
1838_	hsa-mir-891b	MI0005534	hsa-miR-891b-5p	MIMAT0004913	na	na
1839_	hsa-mir-892a	MI0005528	na	na	hsa-miR-892a-3p	MIMAT0004907
1840_	hsa-mir-892b	MI0005538	na	na	hsa-miR-892b-3p	MIMAT0004918
1841_	hsa-mir-892c	MI0022560	hsa-miR-892c-5p	MIMAT0025857	hsa-miR-892c-3p	MIMAT0025858
1842_	hsa-mir-9-1	MI0000466	hsa-miR-9-5p	MIMAT0000441	hsa-miR-9-3p	MIMAT0000442
1843_	hsa-mir-9-2	MI0000467	hsa-miR-9-5p	MIMAT0000441	hsa-miR-9-3p	MIMAT0000442
1844_	hsa-mir-9-3	MI0000468	hsa-miR-9-5p	MIMAT0000441	hsa-miR-9-3p	MIMAT0000442
1845_	hsa-mir-920	MI0005712	na	na	hsa-miR-920-3p	MIMAT0004970
1846_	hsa-mir-921	MI0005713	hsa-miR-921-5p	MIMAT0004971	na	na
1847_	hsa-mir-922	MI0005714	na	na	hsa-miR-922-3p	MIMAT0004972
1848_	hsa-mir-924	MI0005716	hsa-miR-924-5p	MIMAT0004974	na	na
1849_	hsa-mir-92a-1	MI0000093	hsa-miR-92a-1-5p	MIMAT0004507	hsa-miR-92a-3p	MIMAT0000092
1850_	hsa-mir-92a-2	MI0000094	hsa-miR-92a-2-5p	MIMAT0004508	hsa-miR-92a-3p	MIMAT0000092
1851_	hsa-mir-92b	MI0003560	hsa-miR-92b-5p	MIMAT0004792	hsa-miR-92b-3p	MIMAT0003218
1852_	hsa-mir-93	MI0000095	hsa-miR-93-5p	MIMAT0000093	hsa-miR-93-3p	MIMAT0004509
1853_	hsa-mir-933	MI0005755	na	na	hsa-miR-933-3p	MIMAT0004976
1854_	hsa-mir-934	MI0005756	hsa-miR-934-5p	MIMAT0004977	na	na
1855_	hsa-mir-935	MI0005757	na	na	hsa-miR-935-3p	MIMAT0004978
1856_	hsa-mir-936	MI0005758	hsa-miR-936-5p	MIMAT0004979	na	na
1857_	hsa-mir-937	MI0005759	hsa-miR-937-5p	MIMAT0022938	hsa-miR-937-3p	MIMAT0004980
1858_	hsa-mir-938	MI0005760	hsa-miR-938-5p	MIMAT0004981	na	na
1859_	hsa-mir-939	MI0005761	hsa-miR-939-5p	MIMAT0004982	hsa-miR-939-3p	MIMAT0022939

1860_	hsa-mir-940	MI0005762	na	na	hsa-miR-940-3p	MIMAT0004983
1861_	hsa-mir-941-1	MI0005763	na	na	hsa-miR-941-3p	MIMAT0004984
1862_	hsa-mir-941-2	MI0005764	na	na	hsa-miR-941-3p	MIMAT0004984
1863_	hsa-mir-941-3	MI0005765	na	na	hsa-miR-941-3p	MIMAT0004984
1864_	hsa-mir-941-4	MI0005766	na	na	hsa-miR-941-3p	MIMAT0004984
1865_	hsa-mir-942	MI0005767	hsa-miR-942-5p	MIMAT0004985	hsa-miR-942-3p	MIMAT0026734
1866_	hsa-mir-943	MI0005768	na	na	hsa-miR-943-3p	MIMAT0004986
1867_	hsa-mir-944	MI0005769	na	na	hsa-miR-944-3p	MIMAT0004987
1868_	hsa-mir-95	MI0000097	hsa-miR-95-5p	MIMAT0026473	hsa-miR-95-3p	MIMAT0000094
1869_	hsa-mir-96	MI0000098	hsa-miR-96-5p	MIMAT0000095	hsa-miR-96-3p	MIMAT0004510
1870_	hsa-mir-98	MI0000100	hsa-miR-98-5p	MIMAT0000096	hsa-miR-98-3p	MIMAT0022842
1871_	hsa-mir-99a	MI0000101	hsa-miR-99a-5p	MIMAT0000097	hsa-miR-99a-3p	MIMAT0004511
1872_	hsa-mir-99b	MI0000746	hsa-miR-99b-5p	MIMAT0000689	hsa-miR-99b-3p	MIMAT0004678

na, not applicable.

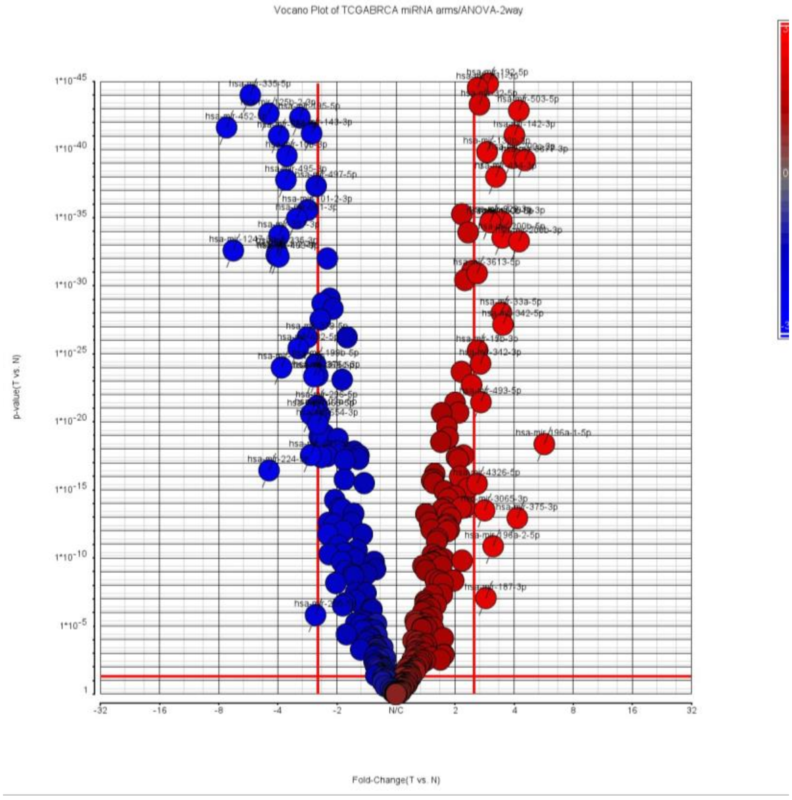

(a)

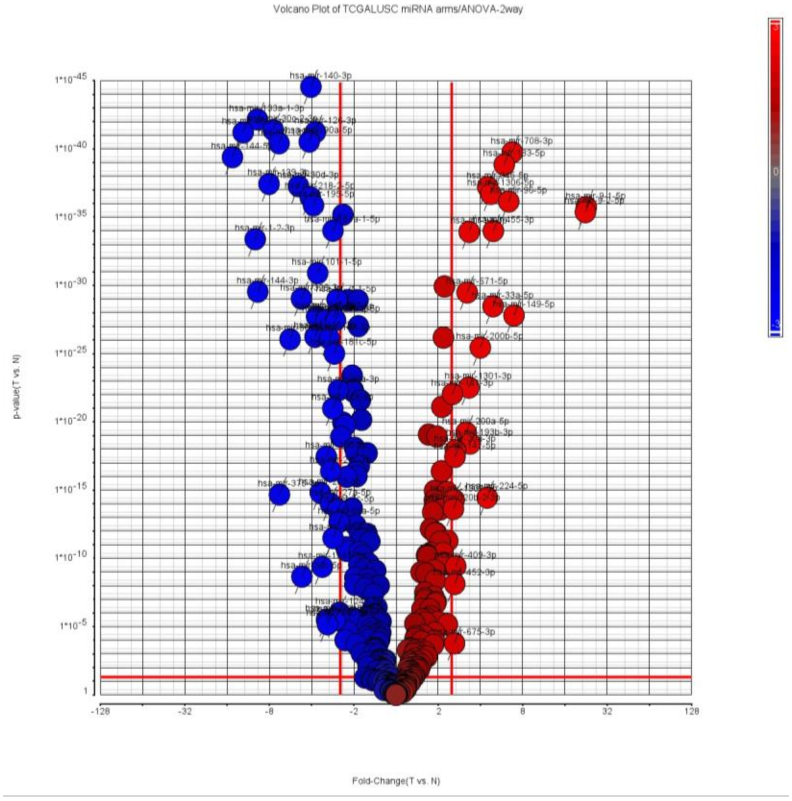

(b)

Figure S1. *Cont*.

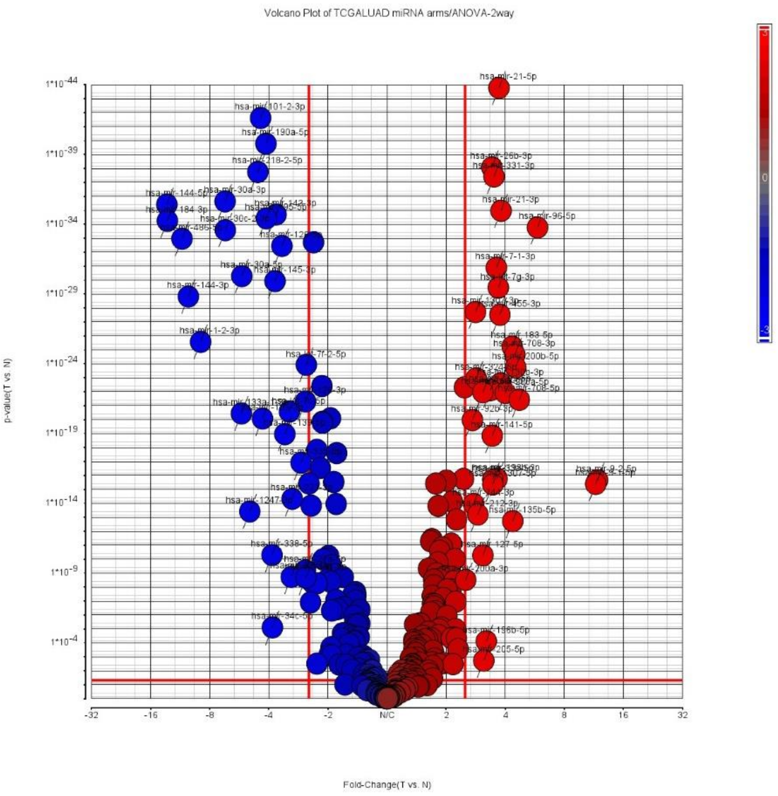

(c)

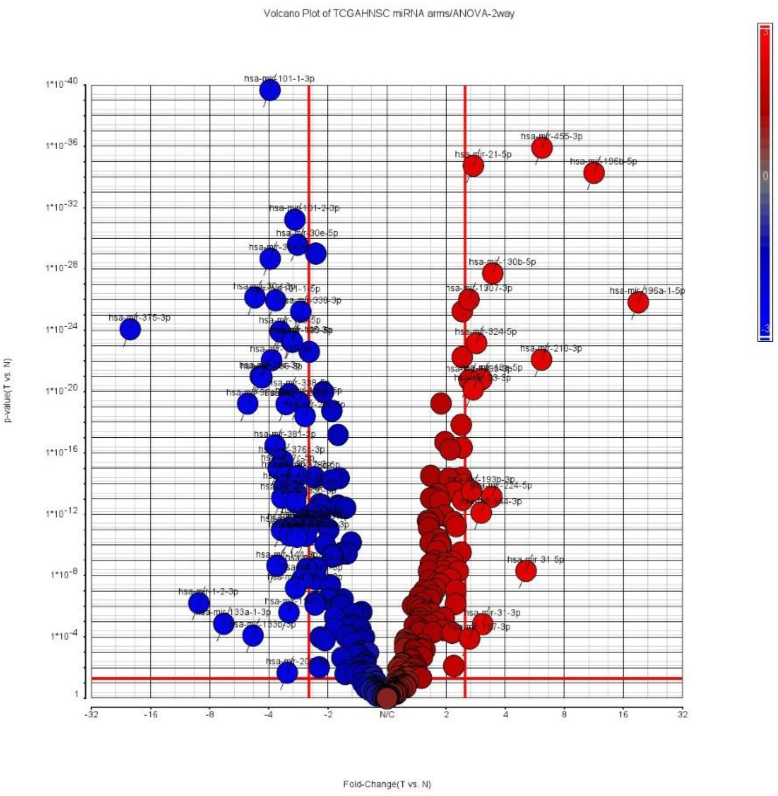

(d)

Figure S1. *Cont.*

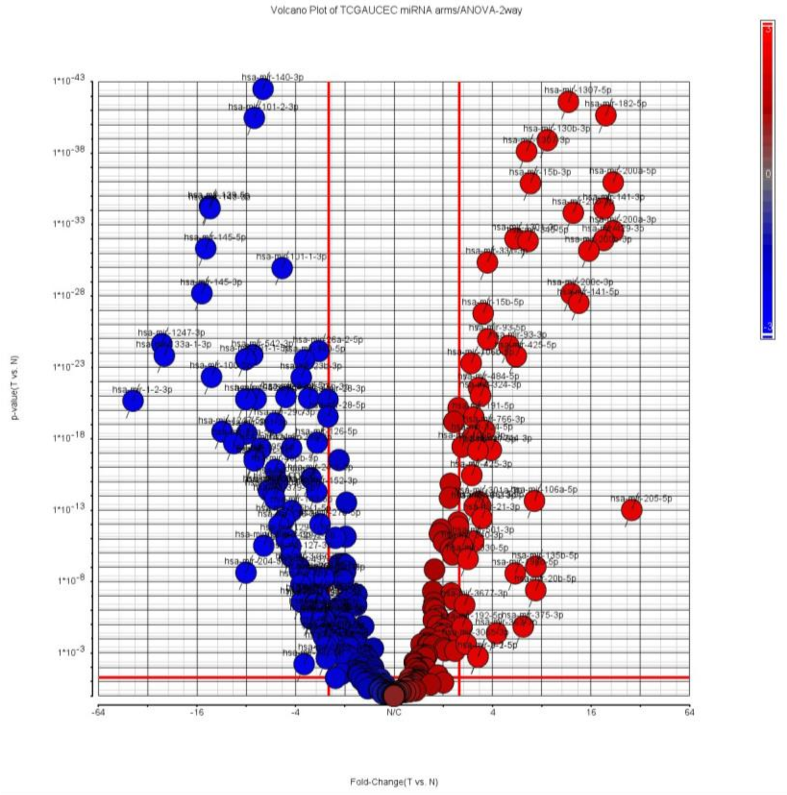

(**e**)

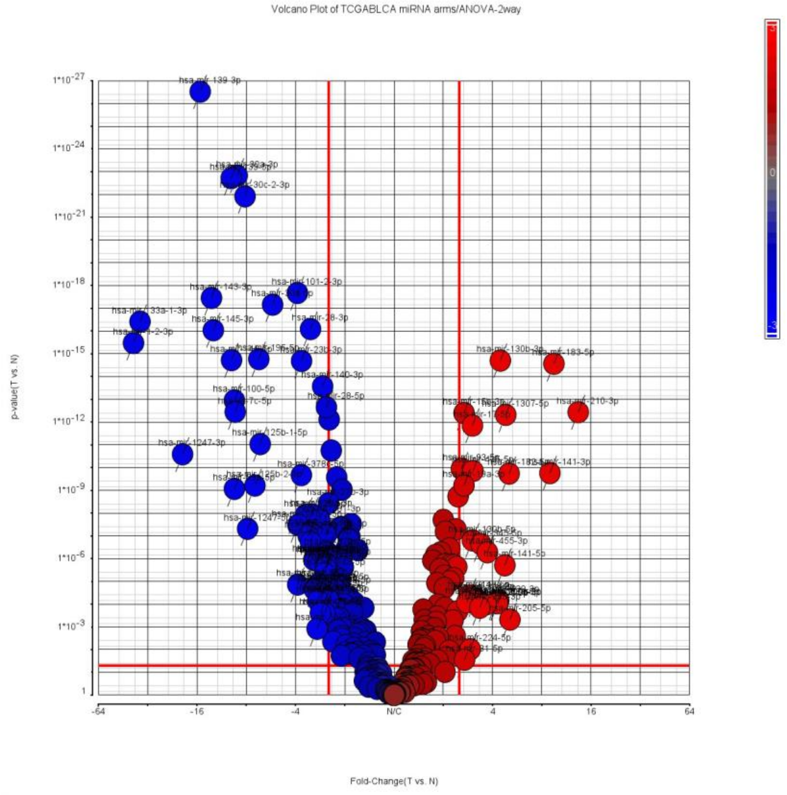

(**f**)

Figure S1. *Cont.*

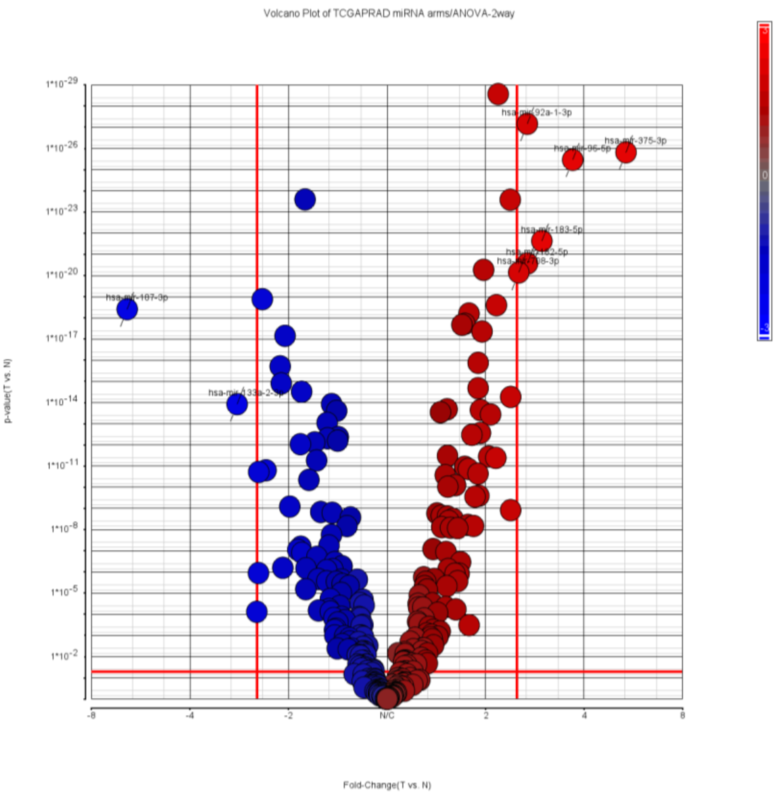

(g)

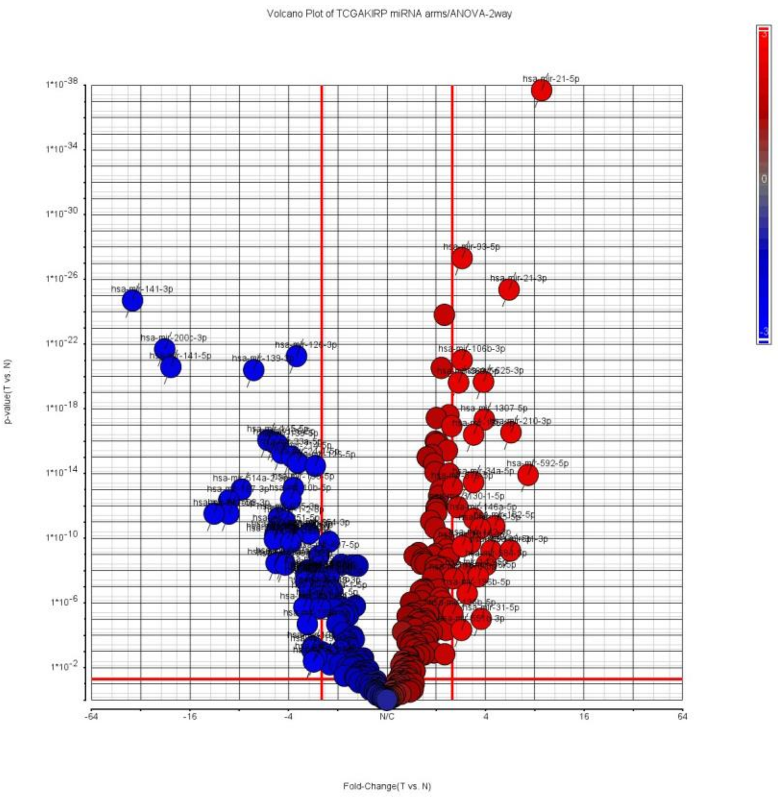

(h)

Figure S1. *Cont.*

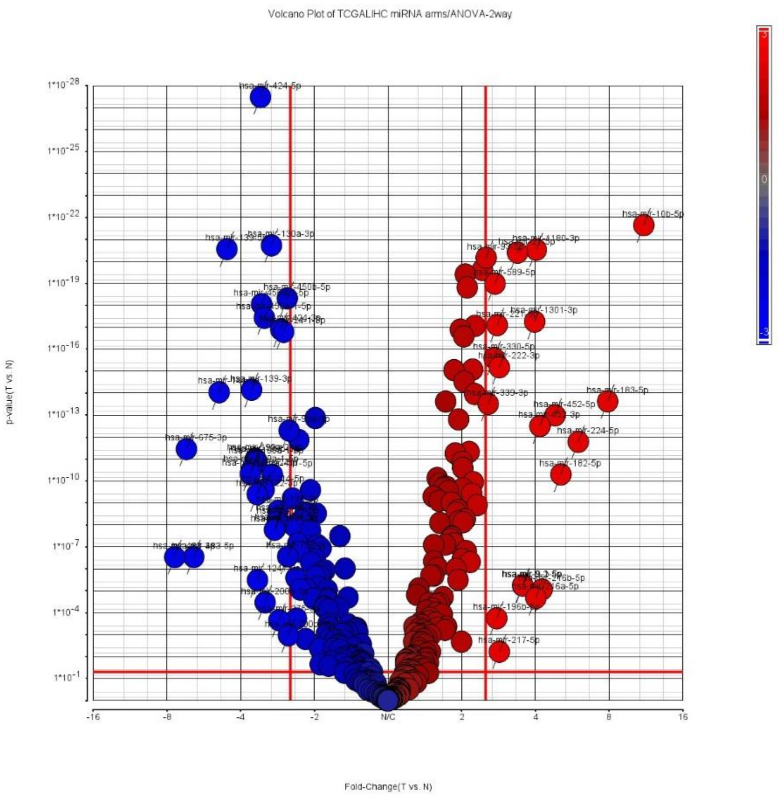

(i)

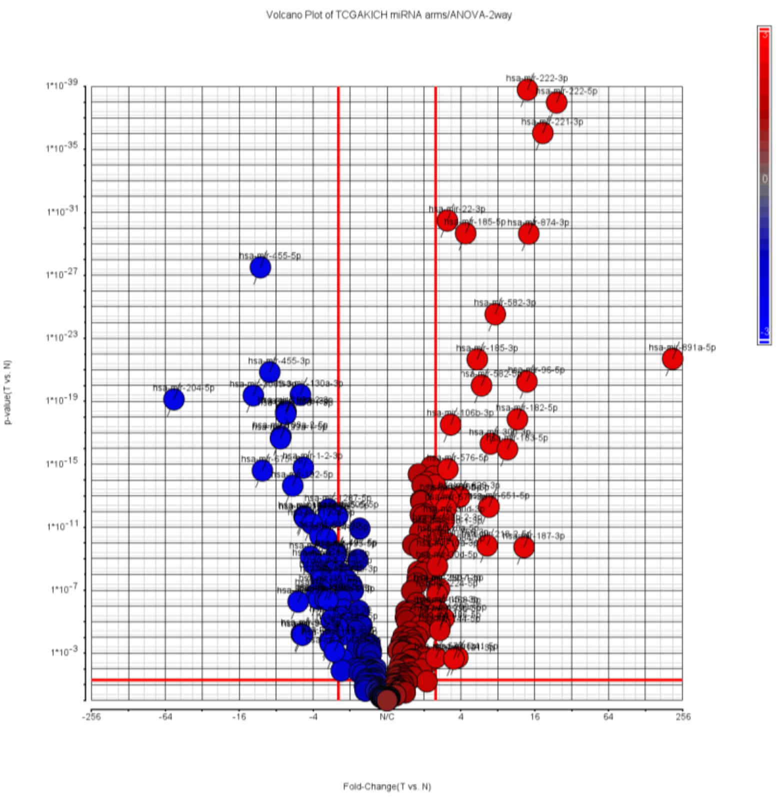

(j)

Figure S1. *Cont.*

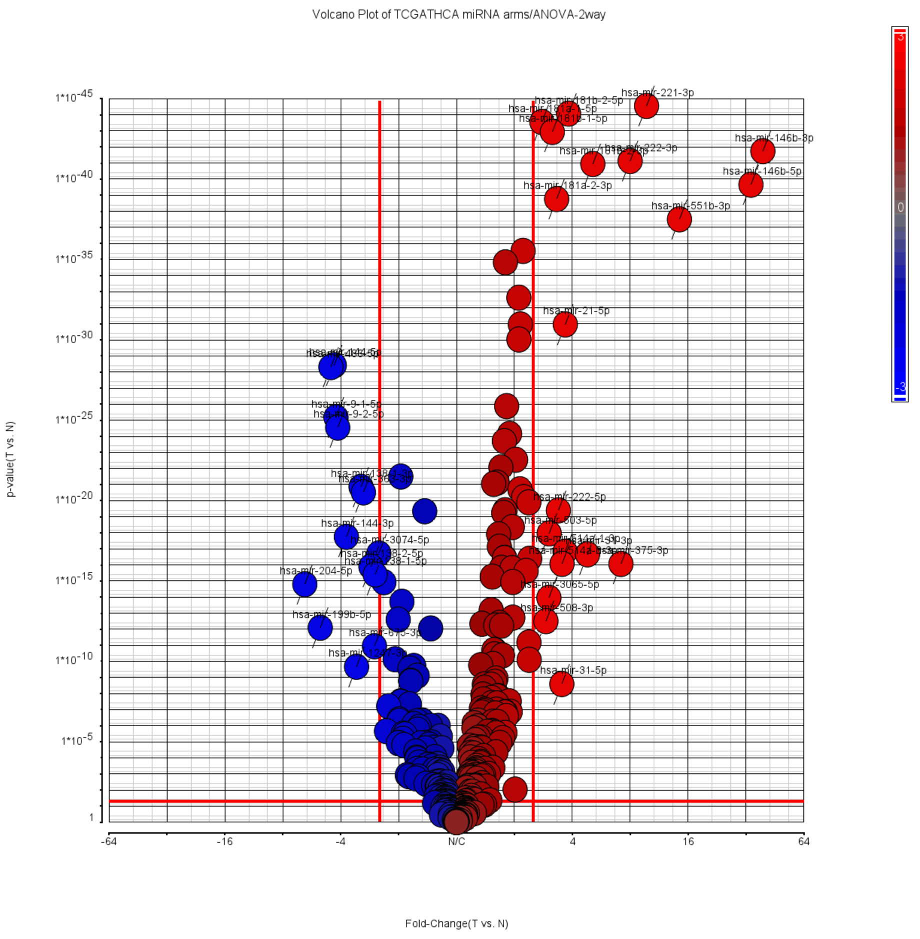

(k)

Figure S1. *Cont.*

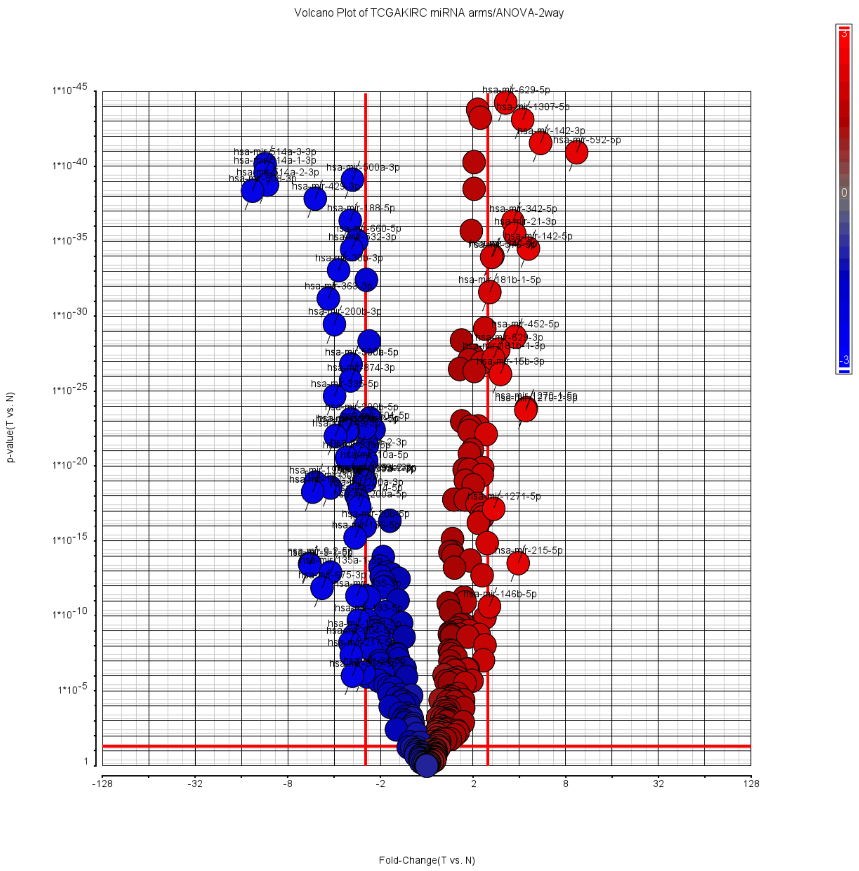

(**l**)

Figure S1. Volcano plots of separate 5p-arm miRNA and 3p-arm miRNA expression profiles in 12 TCGA (The Cancer Genome Atlas) cancer types. miRNA expression data of 12 TCGA cancers were retrieved and re-analyzed using the comprehensive arm feature annotated miRNA list. Following analysis of variance (ANOVA) in the Partek software package, the volcano plot is display for selecting differentially expressed 5p-arm and 3p-arm miRNAs (fold-change > 2.5 and *p*-value < 0.05). (**a**) BRCA, breast invasive carcinoma; (**b**) LUSC, lung squamous cell carcinoma; (**c**) LUAD, lung adenocarcinoma; (**d**) HNSC, head and neck squamous cell carcinoma; (**e**) UCEC, uterine corpus endometrial carcinoma; (**f**) BLCA, bladder urothelial carcinoma; (**g**) PRAD, prostate adenocarcinoma; (**h**) KIRP, kidney renal papillary cell carcinoma; (**i**) LIHC, liver hepatocellular carcinoma; (**j**) KICH, kidney chromophobe carcinoma; (**k**) THCA, thyroid carcinoma; (**l**) KIRC, kidney renal clear cell carcinoma.

© 2015 by the authors; licensee MDPI, Basel, Switzerland. This article is an open access article distributed under the terms and conditions of the Creative Commons Attribution license (http://creativecommons.org/licenses/by/4.0/).
